# Independent degradation in genes of the plastid *ndh* gene family in species of the orchid genus *Cymbidium* (Orchidaceae; Epidendroideae)

**DOI:** 10.1371/journal.pone.0187318

**Published:** 2017-11-15

**Authors:** Hyoung Tae Kim, Mark W. Chase

**Affiliations:** 1 College of Agriculture and Life Sciences, Kyungpook University, Daegu, Korea; 2 Jodrell Laboratory, Royal Botanic Gardens, Kew, Richmond, Surrey, United Kingdom; The National Orchid Conservation Center of China; The Orchid Conservation & Research Center of Shenzhen, CHINA

## Abstract

In this paper, we compare *ndh* genes in the plastid genome of many *Cymbidium* species and three closely related taxa in Orchidaceae looking for evidence of *ndh* gene degradation. Among the 11 *ndh* genes, there were frequently large deletions in directly repeated or AT-rich regions. Variation in these degraded *ndh* genes occurs between individual plants, apparently at population levels in these *Cymbidium* species. It is likely that *ndh* gene transfers from the plastome to mitochondrial genome (chondriome) occurred independently in Orchidaceae and that *ndh* genes in the chondriome were also relatively recently transferred between distantly related species in Orchidaceae. Four variants of the *ycf1-rpl32* region, which normally includes the *ndhF* genes in the plastome, were identified, and some *Cymbidium* species contained at least two copies of that region in their organellar genomes. The four *ycf1-rpl32* variants seem to have a clear pattern of close relationships. Patterns of *ndh* degradation between closely related taxa and translocation of *ndh* genes to the chondriome in *Cymbidium* suggest that there have been multiple bidirectional intracellular gene transfers between two organellar genomes, which have produced different levels of *ndh* gene degradation among even closely related species.

## Introduction

The first two plastid genomes (plastomes) sequenced included the entire *ndh* 11-gene family, which is analogous to complex I in the mitochondrial genome (chondriome) [1, 2]. Subsequently, the function of the *ndh* plastome genes has been described in many studies. The Ndh complex codes for an NADH-specific dehydrogenase with low levels of expression [3, 4], and the family is involved in cyclic electron flow and chlororespiration [4, 5]. Recently, Yamori et al. [6] investigated the function of Ndh complex in low light. However, in spite of this role, the Ndh complex is dispensable for plant growth under optimal conditions [4], and an alternative cyclic electron transport pathway has been reported [7, 8]. Therefore, it has been suggested that *ndh*-lacking species in which at least one of *ndh* genes is non-functional may be able to use the alternative pathway for cyclic electron transport [9].

When the loss of the 11 *ndh* genes in *Pinus thunbergii* was reported [10], this striking feature was considered unique because *ndhF* had been found to be present in all other major sequenced vascular plant clades [11]. However, losses of *ndh* gene function have subsequently been reported in various clades of land plants. In bryophytes, the 11 *ndh* genes in the parasitic liverwort, *Aneura mirabilis* (synonym, *Cryptothallis mirabilis*), were partially or completely deleted [12], and *ndhF* of the leafy liverwort, *Ptilidium pulcherrimum*, was found to be a pseudogene [13]. In the fern clade, some leptosporangiate ferns had internal stop codons in *ndh* genes, but this seemed to be related RNA editing [14–16]. In gymnosperms, *ndh* gene losses have been reported in Pinaceae [10, 17–19] and Gnetales [20, 21]. Parasitic angiosperms have lost the function of *ndh* genes as well as other photosynthesis-related genes [22–25], but some autotrophs also lack the *ndh* gene [26–29].

**Fig 1:**
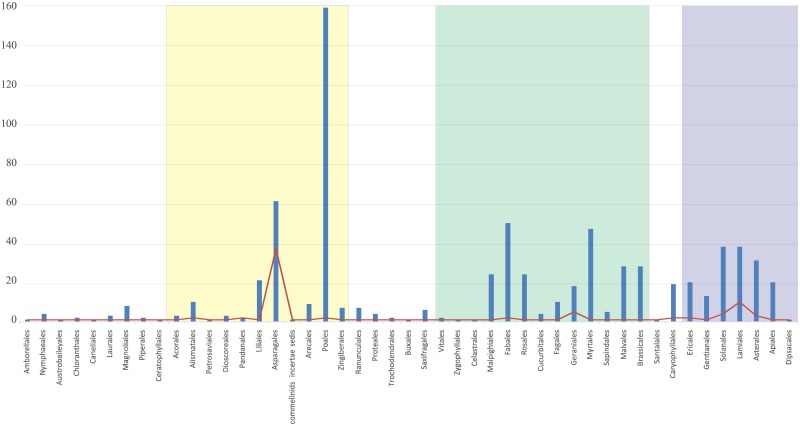
Degradation patterns for 11 *ndh* genes in 743 angiosperm plastomes. To compare the *ndh* gene degradation patterns, the state of *ndh* genes was scored as follows. 1: in-frame gene, 2: pseudogene due to presence of substitutions or short insertion-deletion mutations, 3: highly truncated gene, 4: completely deleted gene. A line refers to the percentage of different *ndh* degradation patterns and the bar refers to the percentage of plastome sequences in orders among the 743 plastomes. The yellow, green and blue boxes represent monocotyledons, rosids and asterids, respectively.

Degradation of *ndh* in Orchidaceae is noteworthy from the perspective of the 11 *ndh* genes found in 743 angiosperm plastomes (Fig 1) (S1 Table). All 11 *ndh* genes had been coded into four classes [30], and different coding *ndh* gene patterns have been in each order based on the extent to which *ndh* genes were variously degraded. Reported plastome sequences of rosids comprise 32.5% of the 743 plastid genomes, but only the rosid order Geraniales have degraded *ndh* genes [28, 31]. With the exception of internal stop codons caused by 1-bp insertions or deletions (indels) in Asterales [32, 33], *ndh* gene degradation in the asterids is restricted to parasitic taxa in Lamiales and Solanales [23, 24, 34–37]. In monocots, the number of sequenced Poales is 21.4% of angiosperms, but only *ndhA* in some species seems to be a pseudogene caused by short indels.

In contrast, among Asparagales, in which most of the sequenced species are orchids, *ndh* degradation patterns vary considerably. Even though many orchids have all 11 *ndh* genes intact in their plastomes [9, 30, 38], a number of degraded *ndh* genes in photosynthetic orchids have been reported [9, 30, 39–44] in addition to those in non-photosynthetic Orchidaceae [45–49]. This result demonstrates that more *ndh* genes in Orchidaceae have been independently modified than in any other family of angiosperms. Therefore, to understand better *ndh* gene degradation, we focus here on orchid plastomes.

Degradation of *ndh* genes among genera in Orchidaceae seems to be independent [9, 30], but the scale of variation among closely related species level has yet to be investigated. The plastomes of the two *Phalaenopsis* species sequenced had similar *ndh* gene degradation patterns [41], which was observed as well as in the plastome of *Phalaenopsis* hybrids [30]. Most *ndh* genes in the eight species of *Cymbidium* sequenced were full-length, although some of them had frame-shift mutations that render them functionless [43]. Degradation of *ndh* in subtribe Oncidiinae varied slightly among genera [40]. However, 15 of the reported Oncidiinae were complex hybrids, and it was difficult to determine the ancestral character status of *ndh* gene degradation among these. Comparative analysis of ten species of coralroot orchids [48] and two species of a distantly related genus, *Epipogium* [49], all of which are holomycoheterotrophic, indicated *ndh* genes had become pseudogenes or were completely deleted in each of their common ancestors. However, recently submitted plastome sequences of *Cymbidium* in GenBank showed different *ndh* gene deletions among individuals within species. Therefore, it seems that *ndh* genes in *Cymbidium* may be being actively degraded and that an investigation of *ndh* gene status will help us understand broader patterns of *ndh* gene degradation in Orchidaceae.

In this paper, 11 *ndh* loci among 23 *Cymbidium* species including hybrids and three closely related taxa are analyzed for *ndh* gene degradation. Except for *ndhF*, we tried to investigate all *ndh* genes. The *ndhF* gene was completely deleted in some species in *Cymbidium* or contained a number of internal homopolymer regions, which we assume indicates non-functional genes. Therefore, we confirmed only the presence of *ndhF* in each plastome. Additionally, we analyzed NGS data to determine if *ndh* genes had been translocated to the chondriome [9] because we found multiple copies of some *ndh* genes in *Cymbidium* species in our investigations.

## Results

### Ten *ndh* loci among 23 *Cymbidium* species and three closely related taxa

Four regions (*ndhB*, *ndhJ-K-C*, *ndhD*, *ndhE-G-I-A-H*) that included ten *ndh* genes from 23 *Cymbidium* species and three outgroups were amplified by PCR and sequenced (Table 1). However, some intergenic or coding regions could not be sequenced because they contained homopolymers and polyA/T-polyG/C or problematic secondary structure (inverted repeats). To identify indels in ten *ndh* genes among 23 *Cymbidium* species and three closely related taxa, the fully intact (functional) *ndh* genes of *Masdevallia coccinea* were used as reference sequence.

**Table 1:** Taxa list for this study.

Subgenus ^a^	Section ^b^	Species	DNA bank number or living collection number
*Cymbidium*	*Austrocymbidium*	*Cymbidium madidum*	O-1472
	*Bigibbarium*	*Cymbidium devonianum*	*
	*Cymbidium*	*Cymbidium atropurpureum*	O-1465
	*Cymbidium*	*Cymbidium finlaysonianum*	1954–41302 BEAK
	*Floribundum*	*Cymbidium floribundum*	O-1461
	*Floribundum*	*Cymbidium pumilum*	O-1469
	*Floribundum*	*Cymbidium suavissimum*	O-1467
	*Himantophyllum*	*Cymbidium dayanum*	O-1468
*Cyperorchis*	*Annamaea*	*Cymbidium erythrostylum*	O-1471
	*Cyperorchis*	*Cymbidium eburneum*	O-1505
	*Cyperorchis*	*Cymbidium elegans*	O-1479
	*Cyperorchis*	*C*. *eburneum* x *C*. *hookerianum*	O-1481
	*Cyperorchis*	*Cymbidium erythraeum*	O-1463
	*Cyperorchis*	*Cymbidium giganteum*	O-69
	*Cyperorchis*	*Cymbidium hookerianum*	O-1466
	*Cyperorchis*	*Cymbidium insigne*	O-1475
	*Cyperorchis*	*Cymbidium iridioides*	O-1462
	*Cyperorchis*	*Cymbidium lowianum*	O-1476
	*Cyperorchis*	*Cymbidium mastersii*	O-1506
	*Cyperorchis*	*Cymbidium sanderae*	O-1470
	*Cyperorchis*	*Cymbidium whiteae*	O-1473
	*Parishiella*	*Cymbidium tigrinum*	17717
*Jensoa*	*Jensoa*	*Cymbidium ensifolium* ^c^	O-1478
	*Jensoa*	*Cymbidium goeringii*	O-1477
	*Jensoa*	*Cymbidium kanran* ^c^	O-1499
	*Jensoa*	*Cymbidium lancifolium* ^c^	O-293
	*Jensoa*	*Cymbidium sinense* ^c^	O-1503
Outgroup		*Acriopsis* sp.	9060
		*Grammatophyllum speciosum*	1983–2947 BACR 450
		*Thecostele secunda*	O-406

^a^: Subgeneric delimitation of *Cymbidium* is based on Du Puy and Cribb [50]

^b^: Sectional delimitation of *Cymbidium* is based on Du Puy and Cribb [50]

^c^: The plastome sequence of these species have been reported by Yang *et al*. [43] and directly submitted by Kim *et al*. in NCBI. Therefore, these four species are used for confirming the location of *ndh* genes to mitochondrial genome.

*: Only fresh leaves were collected from Ratcliffe Orchids, Ltd. (Hampshire, UK)

Except for *C*. *tigrinum* in which only half of exon1 is present and *C*. *mastersii* in which the 5′ region failed to produce sequence, all *Cymbidium* species were documented to contain a full-length *ndhB* gene (S1A Fig). A 1-bp insertion at 37 bp downstream of the 5′ end of *ndhB* results in a frame-shift mutation in *ndhB* in reported plastome sequences of *Cymbidium*, and this was also identified in all *Cymbidium* species studied here and the closely related *Acriopsis* and *Thecostele* accessions (subtribe Cymbidiinae)[51]. A large deletion including exon1, intron and exon2 was detected in *ndhB* of *Acriopsis*.

The *ndhJ-K-C* region was more variable than that of *ndhB* (S1B Fig). A 12-bp direct repeat was distributed 63 bp downstream of the 5′ end of *ndhC* and 69~82 bp downstream of 3′ end of *ndhJ* in most *Cymbidium* species. However, the sequence between the direct repeats was only deleted in *C*. *goeringii*, a result that conflicts with the complete plastome sequence of same species in GenBank (NC_028524), but this was based on a different individual of that species. Deletions caused by direct repeat sequences were also found in the 5′ region of *ndhJ* in three *Cymbidium* species (*C*. *floribundum*, *C*. *erythrostylum*, and *C*. *tigrinum*), *Acriopsis* and *Thecostele*. Unexpectedly, two copies of *ndhJ-K-C* region were detected in *C*. *atropurpureum*. Type I was similar to other *Cymbidium* sequences, whereas type II contained a 87-bp insertion 39 bp downstream of the 5′ end of *ndhK*. This 87 bp insertion is not present in any other of the 743 angiosperm plastomes in GenBank. Only *C*. *madidum*, *C*. *finlaysonianum* and the mt copy of *ndhK* in all *Cymbidium* species contained sequences of this same type.

The *ndhD* regions of *Cymbidium* were relatively conserved (S2A Fig). Large deletions were located in the 3′ region of the gene. Some of these occurred between direct repeat sequences.

The largest deletion of *ndh* genes in *Cymbidium* was identified in the *ndhE-G-I-A-H* region (S2B Fig), the end points of which were commonly located in an extremely AT-rich region. In particular, deletion of *ndhA* exon1 and *ndhH* in *C*. *goeringii* corresponded to those occurring in the plastomes of *C*. *ensifolium*, *C*. *kanran*, *C*. *lancifolium* and *C*. *macrorhizon* even though the plastome of different individuals of *C*. *ensifolium* (NC_028525) and *C*. *goeringii* (NC_028524) contained full length pt-*ndhA* and *ndhH*.

### Different types of the *ycf1-rpl32* region in *Cymbidium*

**Fig 2:**
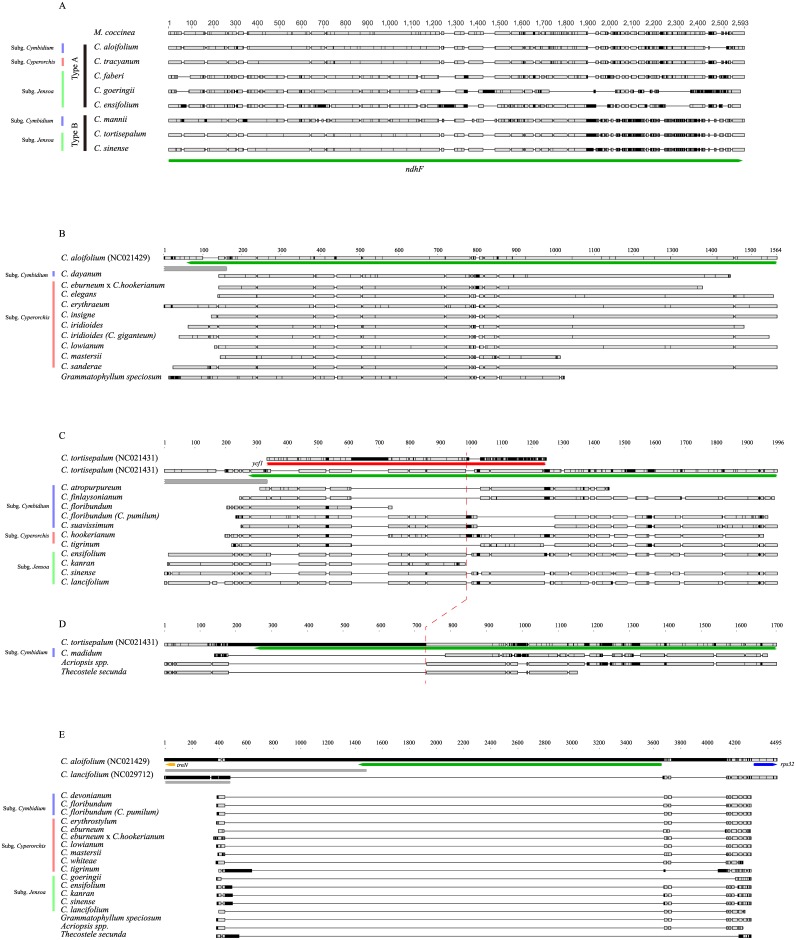
Four types of *ycf1-rpl32* regions in organellar genomes of *Cymbidum* and closely related taxa. The red dotted line refers to identical position of C) the end of replaced *ycf1* and D) the end of deletion in *Acriopsis* and *Thecostele*. A) The *ndhF* genes of currently sequenced plastomes are divided into two groups. Type A is similar to *ndhF* of *Masdevallia coccinea* whereas type B has 420 bp *ycf1*-like region at 3′ region of *ndhF*. B) Type A *ycf1-rpl32* region is more conserved than the others. C) Type B *ycf1-rpl32* regions have a number of deletions. D) The 3′ region of *ndhF* is deleted in the type C *ycf1-rpl32* region. E) Type D *ycf1-rpl32* region completely lacks *ndhF*.

The *ycf1-rpl32* region of the sequenced plastomes of *Cymbidium* was subdivided into two different types in comparison with that of *M*. *coccinea* (Fig 2A). Type A *ycf1-rpl32* was similar to the reference, whereas 420 bp of 3′ region of *ndhF* was replaced with *ycf1* sequence in type B *ycf1-rpl32*.

*Cymbidium dayanum* in subg. *Cymbidium* and nine species of subg. *Cyperorchis* contained type A *ycf1-rpl32*, which was highly conserved (Fig 2B). In contrast to type A *ycf1-rpl32*, type B *ycf1-rpl32* of *Cymbidium* had number of indels in 3′ region of *ndhF* (Fig 2C). The type B *ycf1-rpl32* of *C*. *sinense* sequenced in this paper was only 87% similar to that of *C*. *sinense* plastome owing to many indels. Type B *ycf1-rpl32* was also found in three *Cymbidium* species in which plastid *ndhF* was completely deleted. In comparison to type B *ycf1-rpl32*, type C *ycf1-rpl32* had large deletion in the 3′ region of *ndhF*, and the end point of the deletion corresponded to the end point of the replaced *ycf1* region (Fig 2C and 2D).

Type D *ycf1-rpl32* in which *ndhF* was completely deleted was found in half of the *Cymbidium* species examined and the three closely related taxa with a high level of similarity among them (Fig 2E). In comparison with type A *ycf1-rpl32*, two large deletions occurred in type D *ycf1-rpl32*; one was the complete deletion of *ndhF* and the other was an intergenic deletion between *ndhF* and *rpl32*.

### Multiple copies of *ndh* genes in Orchidaceae

The 38 *ndh* partial sequences were detected from 15 contigs using four sets of NGS data from Orchidaceae (Table 2). With the exception of one contig in *C*. *lancifolium*, the ratio of the depth of mt-*ndh* genes to the depth of plastome in 15 contigs was 5.5~14.5, and BLAST results confirmed that they were derived from the chondriome.

**Table 2:** The information of mt-*ndh* genes assembled from NGS data.

Taxa	Region	Accession	Length	Average depth of mt-*ndh* gene / Average depth of plastome	Reference
***Cymbidium macrorhizon*** **(4 contigs)**	*ndhA*	KX962303	2176	48.3 / 459.2	
	*ndhB*	KX962302	2036	25.5 / 459.2	
	*ndhC*	KX962305	356	39.9 / 459.2	
	*ndhD*	KX962303	236	43.9 / 459.2	
	*ndhE*	KX962303	284	57.4 / 459.2	
	*ndhF*	KX962304	1910	41.4 / 459.2	
	*ndhF*	KX962304	779	31.4 / 459.2	
	*ndhG*	KX962303	497	48.4 / 459.2	
	*ndhH*	KX962303	1127	39.5 / 459.2	
	*ndhI*	KX962303	464	48.0 / 459.2	
	*ndhJ*	KX962305	362	44.0 / 459.2	
	*ndhK*	KX962305	867	46.9 / 459.2	
***Cymbidium lancifolium*** **(6 contigs)**	*ndhA*	KX962298	2199	35.3 / 318.7	
	*ndhB*	KX962296	2047	25.8 / 318.7	
	*ndhC*	KX962301	356	13.1 / 318.7	
	*ndhD*	KX962297	773	23.0 / 318.7	
	*ndhD*	KX962298	236	20.8 / 318.7	
	*ndhE*	KX962298	284	40.2 / 318.7	
	*ndhF*	KX962300	2100	39.8 / 318.7	
	*ndhF*	KX962299	955	32.8 / 318.7	
	*ndhG*	KX962298	497	24.8 / 318.7	
	*ndhH*	KX962298	1127	29.4 / 318.7	
	*ndhI*	KX962298	464	22.3 / 318.7	
	*ndhJ*	KX962301	362	6.4 / 318.7	
	*ndhK*	KX962301	811	6.6 / 318.7	
***Dendrobium catenatum*** **(4 contigs)**	*ndhA*	KX962306	1537	779.9 / 7687.6	SRR2084072
	*ndhA*	KX962306	575	739.8 / 7687.6	SRR2084072
	*ndhC*	KX962309	355	671.5 / 7687.6	SRR2084072
	*ndhD*	KX962307	1323	751.0 / 7687.6	SRR2084072
	*ndhE*	KX962307	306	780.6 / 7687.6	SRR2084072
	*ndhF*	KX962308	1600	738.6 / 7687.6	SRR2084072
	*ndhG*	KX962307	212	1118.4 / 7687.6	SRR2084072
	*ndhH*	KX962306	1155	664.9 / 7687.6	SRR2084072
	*ndhI*	KX962306	501	731.5 / 7687.6	SRR2084072
	*ndhJ*	KX962309	472	626.7 / 7687.6	SRR2084072
	*ndhK*	KX962309	610	636.7 / 7687.6	SRR2084072
***Epipogium aphyllum*** **(1 contig)**	*ndhA*	KX962310	215	28.7 / 216.8	SRR1344939
	*ndhA*	KX962310	425	29.4 / 216.8	SRR1344939
	*ndhI*	KX962310	684	15.1 / 216.8	SRR1344939

The contig that contained the *ndhJ-K-C* region in *C*. *lancifolium* was present in relatively lower depth and did not contain a mitochondrial region, but there were only two SNPs and one indel that differed among the mt-*ndhJ-K-C* region in *C*. *lancifolium* and *C*. *macrorhizon*. Consequently, we concluded all 16 contigs have been translocated from the plastome to the chondriome.

#### Two *Cymbidium* species in section *Pachyrhizanthe*

**Fig 3:**
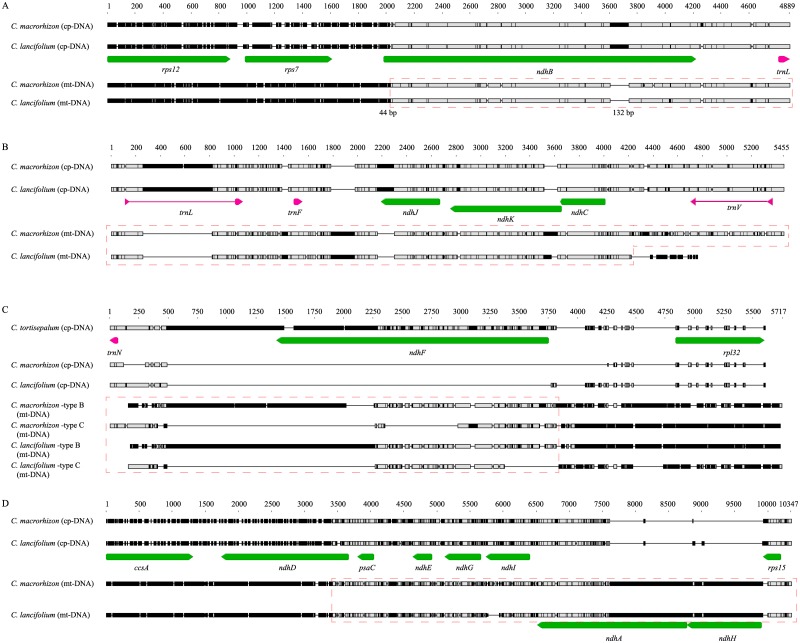
Alignment of *ndh* gene regions in both organellar genomes of *Cymbidium*. A red dotted-box indicates a plastome-like region in the chondriome. A) The plastid *ndhB* region from 44 bp downstream of 5′ end of gene was transferred to chondriome. At exon2 of mt-*ndhB*, a 132 bp deletion was found. B) The mt-*ndhJ-K-C* region contained a large deletion in and a large insertion in mt-*ndhK*. The length variation between two large insertions in mt-*ndhK* was caused by 28 bp tandem repeats. C) In contrast with deleted plastid *ndhF*, two types of mt-*ndhF* were found in both species. D) Both *ndhA* exon1 and *ndhH* were deleted in the plastome, whereas they were found in the chondriome of both species.

All 11 *ndh* genes have been found in the chondriome of two *Cymbidium* species, and most of them do not differ in these two species. The mt-*ndhB* gene lacked 44 bp of exon1 and contained a 132-bp deletion in exon2 (Fig 3A). Similarities of the *ndhB* genes in the same genome among different species were 99.0 and 99.5%. However, those in the genomes of two accessions of same species were only 91.1 and 91.9% similar. Mt-*ndhJ* and *ndhK* contained a large deletion and insertion, respectively (Fig 3B). The length variation of insertion in mt-*ndhK* between two *Cymbidium* species was due to tandem repeats of 28 bp sequence. Even though plastid *ndhF* was completely deleted, two copies of mt-*ndhF* were found in two *Cymbidium* species (Fig 3C). One copy of these was similar to *ndhF* in type B *ycf1-rpl32*, and the other was similar to *ndhF* in type C *ycf1-rpl32*. In comparison with their plastome sequence, mt-*ndhD* was truncated and mt-*ndhA* and *ndhH* genes were almost full length (Fig 3D). In addition, another mt-*ndhD* (773 bp) was found in *C*. *lancifolium*.

#### Dendrobium catenatum

The nine mt-*ndh* genes were found in four large contigs (Table 2). Among them, three contigs could form subgenomic circles [52]. Because a number of pt-*ndh* genes of *D*. *catenatum* have been deleted [53], we used a completely intact set of pt-*ndh* genes as a reference sequence, in this case *Sobralia*.

**Fig 4:**
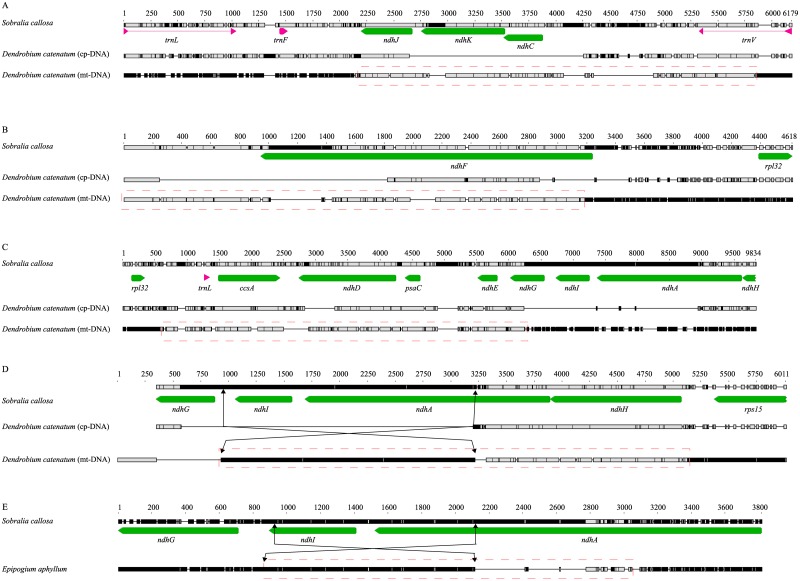
Alignment of *ndh* gene regions in *Dendrobium catenatum* and *Epipogium aphyllum*. A red dotted-box indicates a plastome-like chondriome region. The plastid *ndh* genes of *Sobralia callosa* were used as reference. A) In contrast with plastid *ndhJ-K-C* region of *D*. *catenatum*, mt-*ndhJ-K-C* region was similar to the reference in length with the exception of deletion in mt-*ndhK*. B) Both plastid and mt-*ndhF* of *D*. *catenatum* contained large deletions. C) The plastid region of *D*. *catenatum* from downstream of *rpl32* to downstream of 5′ end of *ndhG* was transferred to the chondriome. D) The *ndhI-A-H* region in the chondriome of *D*. *catenatum* has a mt-*ndhI-A* exon2 region that is inverted relative to the reference, whereas this region was completely deleted in the plastome. E) Plastome of *E*. *aphyllum* has completely deleted all 11 *ndh* genes, whereas its chondriome has retained an *ndhI-A* region; there was an inversion between 3′ region of *ndhI* and upstream of 5′ end *ndhA* exon2.

The region of mt-*ndhJ-K-C* was similar to the reference sequence in length with the exception of a large deletion in mt-*ndhK*, whereas pt-*ndhK* and *ndhC* were completely absent (Fig 4A). Mt-*ndhF* was longer than pt-*ndhF*, but both of them were highly truncated (Fig 4B). The regions between 194 bp downstream of *rpl32* and 317 bp downstream of the 5′ end of *ndhG* were relatively conserved between pt- and mt-*ndh* genes, but the 3′ region of *ndhD* had a large deletion in both genomes (Fig 4C). The regions with pt-*ndhI* and *ndhA* exon2 were deleted [53], whereas these genes were found in chondriome but with a large inversion upstream of 5′ end of *ndhG* and downstream of the 5′ end of *ndhA* (Fig 4D).

#### Epipogium aphyllum

We found mt-*ndhI* and *ndhA* genes in achlorophyllous (holomycotrophic) *E*. *aphyllum*, but all pt-*ndh* genes in this species were completely deleted [49]. Unexpectedly, there was also an inversion mutation like that found in mt-*ndhI-A* of *D*. *catenatum* (Fig 4E).

### Phylogenetic relationships between pt- and mt-*ndh* genes in Orchidaceae

In most *ndh*-gene trees (S3 Fig), the mt-*ndh* genes of *Cymbidium* formed a clade. It was noteworthy that the clustering of mt-*ndhD*, *ndhE* and *ndhG* from the NGS data and direct sequencing was strongly supported. However, the mt-*ndhH* genes of section *Pachyrhizanthe* formed a clade with the pt-*ndhH* genes of previously sequenced *Cymbidium* plastomes [43], whereas all pt-*ndhH* genes of *Cymbidium* sequenced in this study formed a strongly supported cluster. In addition, the *ndhJ*, *ndhK and ndhC* genes of *C*. *madidum*, *C*. *finlaysonianum* and type II *C*. *atropurpureum* formed a cluster with mt-*ndhJ*, *ndhK* and *ndhC* of section *Pachyrhizanthe*. The second copy of mt-*ndhD* in *C*. *lancifolium* clustered with the mt-*ndhD* of *Oncidium*, and they formed a strongly supported group with other orchid mt-*ndhD* genes. The clustering of the pt-*ndhG* of *C*. *ensifolium* (NC_028525) and mt-*ndhG* from other species of *Cymbidium* was strongly supported, whereas another pt-*ndhG* from *C*. *ensifolium* (KU179434) formed a group with pt-*ndhG* in *Cymbidium*.

Multiple copies of the mt-*ndh* genes from *Erycina pusilla* (subtribe Oncidiinae) formed a unique cluster with the exception of one copy of mt-*ndhD* (246 bp), which was relatively shorter than other mt-*ndhD* genes (480~1078 bp) in *E*. *pusilla*. Furthermore, these mt-*ndh* genes clustered with their pt-counterparts with the exception of pt-*ndhA*, *ndhI* and *ndhE*, which were truncated or missing from the plastome of *E*. *pusilla*.

The mt-*ndhA*, *ndhD*, *ndhE*, *ndhG*, *ndhH*, *ndhI* and *ndhJ* genes in *Masdevallia picturata* were most closely related to the pt-*ndh* genes of *Masdevallia*, and almost all mt-*ndh* genes in *Paphiopedilum* also formed clusters with the pt-*ndh* genes of these species.

## Discussion

### Patterns of *ndh* degradation in *Cymbidium*

Function of *ndh* genes has been independently lost in some orchid clades [9, 30]. With the exception of the directly sequenced plastomes of *Goodyera*, *ndh*-missing/non-intact species and *ndh*-intact species have not been so far found in same genus of Orchidaceae [41, 43, 48], in contrast to the situation in *Erodium* [27, 28]. Therefore, loss of function in the *ndh* complex seems to have occurred in the common ancestor of the *ndh*-missing/non-intact species within those genera rather than independently at the species level. The situation for *ndhB* in *Cymbidium* indirectly supports this scenario. With the exception of inverted repeat (IR)-deleted species, this gene is normally located in the IR, which position seems to play a role in its structural stability [54]. Substitution rates of the IR are also lower than those of single copy regions [55–59]. Therefore, *ndhB* is structurally more conserved than other *ndh* genes that are located in the single copy regions. In *Cymbidium* species, a 1-bp insertion at 37 bp downstream of the 5′ end of *ndhB* has been found in all species with the exception of the species that contain a truncated copy of *ndhB*. Therefore, at least, the ancestor of all *Cymbidium* species is likely to have lacked a functional *ndh* complex.

The first sequenced plastomes of *Cymbidium* [43] and directly uploaded sequences (NC_028525 and NC_028524) contained full-length *ndh* genes even though most of them were pseudogenes due to frameshift mutations. However, recently a sequenced plastome of *Cymbidium* lacked pt-*ndhF*, *ndhH* and *ndhA* exon1. As a result, there are two plastomes of *C*. *ensifolium* with different *ndh* gene content. With the exception of technical errors (misidentification at the time of collection or laboratory errors), which is difficult to determine in this study, our results support the hypothesis that *Cymbidium* species have undergone dynamic and recent *ndh* gene degradation. Because the common ancestor of all *Cymbidium* species seems to have lacked *ndh* function, many different substitutions and indels may have accumulated in the various species due to relaxed selection. The large deletions that caused *ndh* degradation should be shared between closely related taxa if *ndh* gene degradation had occurred in an ancestral pseudogene further in the past. However, most of the large deletions detected are unique in each accession.

In addition, one of the main factors involved in *ndh* gene degradation is likely to be intracellular recombination. A number of deletions have been found between direct repeat sequences or extremely AT-rich (homopolymer) regions. These patterns have been known to relate to intramolecular recombination [60, 61] and illegitimate recombination [62], respectively. These results suggest that the plastomes in *Cymbidium* species have undergone independent *ndh* gene degradation, probably after they speciated. The different levels of plastid *ndh* gene degradations in different individuals of *C*. *ensifolium* and *C*. *goeringii* also support a hypothesis of recent *ndh* gene degradation in *Cymbidium*.

However, we cannot suggest a clear explanation for why there appears to be a recent burst in this activity in the extant species of *Cymbidium*. In contrast, the *ndh*-lacking genera of photosynthetic orchids, i.e. *Phalaenopsis* [41], *Oncidium*, *Paphiopedilum* [30], *Dendrobium* and *Bletilla*, have retained similar *ndh* gene degradation patterns among their species. In general, with the exception of extremely reduced mycoheterotrophic orchids [45, 49], a number of pseudogenes have been retained in the plastomes of Orchidaceae [46–48]. In particular, the closely related green and non-green coralroot orchids (*Corallorhiza*), which have lost some *ndh* genes, are similar in plastid genome size [48]. Therefore, the plastome of Orchidaceae may be prone to retain its size due to some selective constraints.

Barrett *et al*. [47] hypothesised that non-functional genes in mycoheterotrophic plants may have undergone point mutations and frame-shift mutations under relaxed selective pressure over time, and large deletions occur rarely after purifying selection on non-functional genes ceases. Unlike other genera in Orchidaceae, the most recent common ancestor (MRCA) of *Cymbidium* seems to have been under selective genome size constraint even though *ndh* function had been lost. However, structural mutations like bidirectional homologous recombination between the two organellar genomes or gene conversion in *ndhF* after splitting of populations or speciation might have led the plastome to be under relaxed selective constraints. As a result, it is likely that dynamic *ndh* gene degradation has occurred among *Cymbidium* species, perhaps even among populations.

### Diverse *ndhF* genes result from gene conversion and indels

The first five *Cymbidium* species studied previously had full-length plastid *ndhF* genes [43], but *ndhF* deletions occurred in four recently submitted sequences. As we reported for the *ndhA-H* region, the deleted pt-*ndhF* genes of *C*. *lancifolium* and *C*. *macrorhizon* were transferred to chondriome (Fig 3C). As a result, *C*. *sinense* contains type B *ycf1-rpl32* in its plastome and type D *ycf1-rpl32* in its chondriome, whereas *C*. *kanran*, *C*. *ensifolium*, *C*. *macrorhizon* and *C*. *lancifolium* contain type D *ycf1-rpl32* in their plastomes and type B *ycf1-rpl32* in their chondriomes. Other *Cymbidium* species also contain different types of *ycf1-rpl32* in their organellar DNAs, but we do not know in which genomes these are located. Species that have the same type of the *ycf1-rpl32* region are not related to each other (i.e. they belong to different clades in the *Cymbidium* phylogenetic tree). Nevertheless, four types of the *ycf1-rpl32* region seem to be related each other.

Type A *ycf1-rpl32* is similar to that of other Orchidaceae, whereas 420 bp of the 3`region of *ndhF* in type B *ycf1-rpl32* is similar to the *ycf1* region and contained a number of indels. The *ndhF* sequence near IR_B_/SSC was replaced with *ycf1* near SSC/IR_A_. This replacement might result from IR expansion via gene conversion [63](S4 Fig). First, recombination was initiated within the IR. Then, a Holliday junction on the IR was moved to SSC, creating heteroduplex DNAs. These heteroduplex DNAs were repaired using the complementary strand as the model. Finally, base substitutions and indels occurred in the *ycf1* like region in *ndhF*. Significantly, an end point for deletion of *ndhF* in *Acriopsis* and *Thecostele* was identical to that of a *ycf1*-like region in *ndhF* of *C*. *tortisepalum* (Fig 2C and 2D). Therefore, it is possible that type C *ycf1-rpl32* was derived from type B *ycf1-rpl32* due to deletion of a chimeric region.

Kim et al. [30] described the important role of *ndhF* in the instability of the IR/SSC junction in Orchidaceae. Retention of full-length *ndhF* seems to be related to the selective constraints that maintain the IR/SSC boundary. The *ndhF* of the type B *ycf1-rpl32* region is similar to *ndhF* in type A *ycf1-rpl32* in length, but in its content is similar to the truncated version of *ndhF* due to the replacement of 3`end region of *ndhF*. As a result, it seems that gene conversion leads to relaxed selective constraint of the IR/SSC junction, after which truncated *ndhF* versions in type B and type C *ycf1-rpl32* may be followed by *ndhF* deletion as in type D *ycf1-rpl32*.

### Intracellular gene transfers between organellar DNA

Chang et al. [39] confirmed the in-frame sequences of *ndhA*, *ndhF* and *ndhH* that are completely deleted in the plastome of *Phalaenopsis aphrodite* and suggested that they were transferred to nuclear genome. However, in the recently published whole genome of *P*. *equestris* [64], it was shown that there was also no intact *ndh* gene [30]. Subsequently, mt-*ndh* genes were found in many unrelated clades of Orchidaceae [9], and we also found mt-*ndh* genes in several distantly related species. Therefore, intact *ndh* genes that are deleted from the plastome of *Phalaenopsis* are likely to be found in its chondriome. However, this is not surprising because such transfers are known to occur widely in seed plants [65–68].

To evaluate relationships between plastid and mitochondrial copies of *ndh* genes in Orchidaceae, we constructed gene trees (S3 Fig), which gave us information about *ndh* gene transfer, although some nodes are not well resolved. First, it is likely that the transfers of *ndh* genes from plastome to chondriome have usually occurred in the MRCA of the species in each genus. As there is limited *ndh* gene information at the species level, especially for mt-*ndh* genes, it is impossible to infer a time for these transfers. However, many of the pt- and mt-*ndh* genes from a given genus cluster together. For instance, mt-*ndhC*, *ndhD*, *ndhG*, *ndhH* and *ndhJ* of *Erycina pusilla* (subtribe Oncidiinae) were transferred after *Erycina* diverged from its common ancestor with *Oncidium* (subtribe Oncidiinae). The mt-*ndh* genes in *Masdevallia picturata* (subtribe Laeliinae, subfamily Epidendroideae) and *Paphiopedium* (subfamily Cypripedioideae) were also sister to pt-*ndh* genes of species within each genus, respectively.

In the *ndh* tree of *Cymbidium*, most mt-*ndh* genes are distantly located from their pt-*ndh* counterparts, and the entire mt-*ndhD-E-G-I-A-H* region can be assembled from NGS data for two species, which we confirmed by PCR of the mt-*ndhD-E-G* region in six *Cymbidium* species. These mt-*ndh* genes clustered uniquely with strong support. Although the combined *ndh* gene tree for ten species of *Cymbidium* had a different topology from that of combined ITS+*matK* [69], it is clear that the transfer of the *ndh* genes in the single-copy region dates back at least to the common ancestor of these *Cymbidium* species.

Secondly, transfers between the chondriome of photosynthetic orchids have occurred more than once. The mt-*ndhD* genes of *Cymbidium* (Cymbidiinae) and *Erycina* (Oncidiinae) were divided into two groups. The mt-*ndhD* genes (from mt-*ndhD-E-G* region) of *Cymbidium* and *Erycina* clustered with mt-*ndhD* genes in same genus. However, another copy of mt-*ndhD* gene in *C*. *lancifolium* and *Erycina* formed a strongly supported cluster with the mt-*ndhD* genes from *Oncidesa* Gower Ramsey (a complex hybrid between species in *Oncidium* and *Gomesa*, most likely with the plastid genome of the former) and a member of another subfamily *Goodyera fumata* (tribe Cranichidae, subfamily Orchidoideae). These four mt-*ndhD* genes clustered with mt-*ndhD* gene of *D*. *catenatum* (tribe Malaxidae, subfamily Epidendroidae), to which the plastid *ndhD* of *Dendrobium* was an outlier with moderate support. It is therefore likely that mt-*ndhD* of *Dendrobium* has been directly transferred independently to the other four species [70]. In addition, mt-*ndhE* of *Oncidesa* Gower Ramsey (subfamily Epidendroideae) and *V*. *planfolia* (subfamily Vanilloideae) are identical. Although the substitution rate of the chondriome is slower than in plastid DNA [52], it is unlikely that mt-*ndhE* of two species originated in their common ancestor because of the long time, before the end of the Cretaceous, since the members of these orchid subfamilies diverged [70]. Consequently, our results suggest a recent transfer of mt-*ndh* gene between distantly related taxa in Orchidaceae. Horizontal gene transfer (HGT) between photosynthetic orchids has not been reported so far. However, multiple mt-genes from different lineages have been transferred into the chondriome of Geraniaceae [71]. Because there is little information of the chondriome of Orchidaceae, it is difficult to figure out how and when this HGT might have occurred.

### Unidirectional vs bidirectional IGT

The most remarkable feature of *ndh* genes in *Cymbidium* is the presence of multiple copies in their organellar genomes. For example, *C*. *sinense* has a type B *ycf1-rpl32* in its plastome and type D *ycf1-rpl32* in its chondriome, whereas *C*. *kanran*, *C*. *ensifolium*, *C*. *macrorhizon* and *C*. *lancifolium* have type D *ycf1-rpl32* in their plastomes and type B *ycf1-rpl32* in their mt-DNA. Some species also have other types, e.g. *ycf1-rpl32* types A and D. It is highly perplexing that *Cymbidium* species can have different types of the *ycf1-rpl32* region in one genome (plastome or chondriome) and the same type of *ycf1-rpl32* region in different genomes. We have two hypotheses that could explain this phenomenon: *C*. *sinense* and *C*. *macrorhizon* represent non-functional *ndhF* (type A, B and C) and completely *ndhF*-deleted species (type D), respectively.

**Fig 5:**
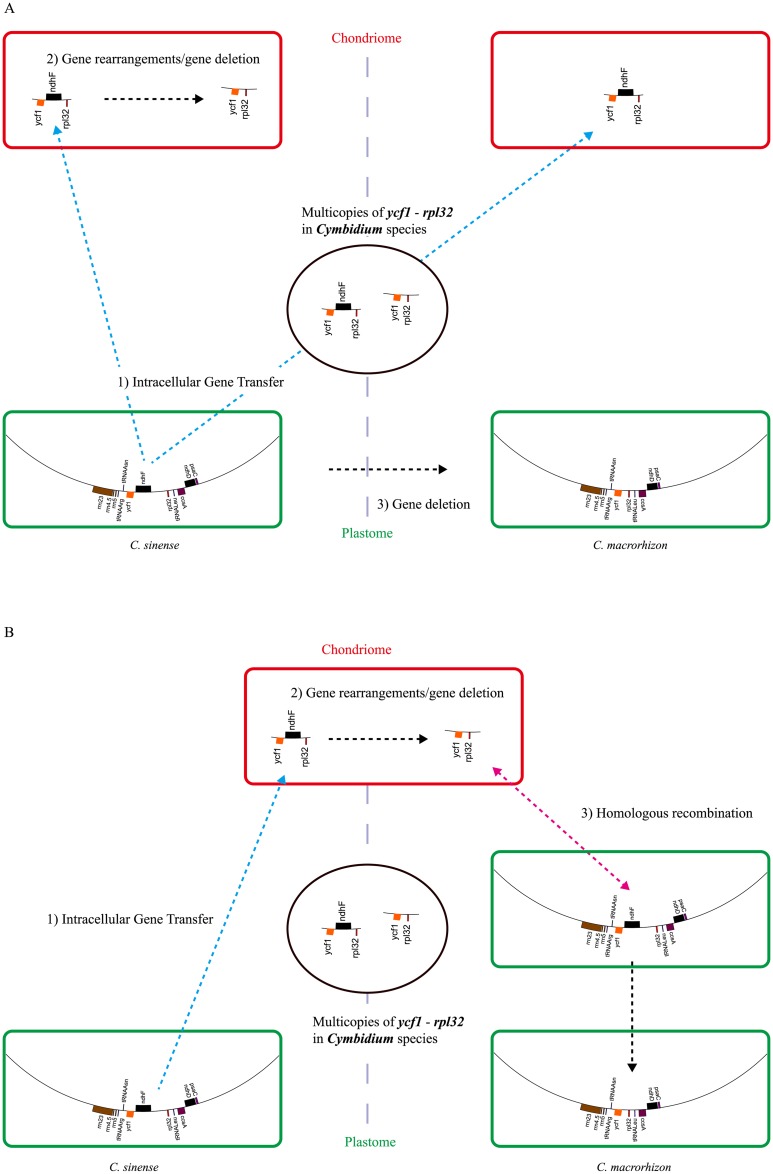
Two hypotheses for multiple copies of *ycf1-rpl32* region in *Cymbidium* species. *C*. *sinense* illustrates the *ndhF*-containing types (type A, B, C), and *C*. *macrorhizon* the *ndhF*-deleted type (type D) in plastome. Green and red boxes indicate plastome and chondriome, respectively. A) The *ycf1-rpl32* region containing the *ndhF* (ancestral type) was transferred to the chondriome, and then mt-*ndhF* (*C*. *sinense*) and plastid *ndhF* (*C*. *macrorhizon*) were independently deleted. B) The *ycf1-rpl32* region containing *ndhF* were transferred to chondriome in the ancestor of the extant species of *Cymbidium* and closely related genera. Then, the mt-*ndhF* was removed from *ycf1-rpl32* via gene rearrangements or gene deletion (*C*. *sinense*). In addition, homologous recombination between two *ycf1-rpl32* regions of the plastome and chondriome occurred in some taxa or populations. As a result, *ndhF* was found not in the plastome but in the chondriome (e.g. in *C*. *macrorhizon*).

The first hypothesis is unidirectional transfer (Fig 5A). The *ycf1-rpl32* region containing *ndhF* (ancestral type) was transferred to its chondriome. Subsequently, the mt-*ndhF* (*C*. *sinense*) and pt-*ndhF* (*C*. *macrorhizon*) were independently deleted. The second hypothesis is bidirectional transfer (Fig 5B). In this scenario, the *ycf1-rpl32* region containing plastid *ndhF* was transferred to chondriome in the ancestor of *Cymbidium* and closely related genera of subtribe Cymbidiinae. After this transfer, the mt-*ndhF* copy was eliminated by gene rearrangements or gene deletion (as in *C*. *sinense*). Some species then underwent homologous recombination between the two *ycf1-rpl32* copies in their plastomes and chondriomes (e.g. *C*. *macrorhizon*).

Type D *ycf1-rpl32* among *Cymbidium* and three closely related taxa is highly conserved and shares two large deletions (Fig 2). The first hypothesis therefore must assume that two deletions in *ycf1-rpl32* in both the plastome and chondriome have occurred at exactly the same position in all *Cymbidium* species and closely related taxa. However, the second hypothesis more easily explains this high level of similarity of the type D *ycf1-rpl32* region among these genera because it originated in their common ancestor and mt-DNA has low substitution rate [52]. Similarly, because the plastid *ndhH* genes of previously sequenced *Cymbidium* plastomes have been re-transferred from chondriome, it is likely that they should cluster with the mt-*ndhH* genes of *Cymbidium* section *Pachyrhizanthe*.

In relative terms, the plastid genome is ten times more abundant that the mitochondrial genome of *D*. *catenatum*. This means that plastid regions are easier to amplify than mt-region even if the mt-region had exactly the same primer binding sites as the plastid copy. With the exception of *C*. *atropurpureum*, only one PCR product of the plastid *ndhJ*-*K*-*C* region was produced from all *Cymbidium* species and three related species studied here, and the plastid copies of *ndhJ*, *ndhK* and *ndhC* all clustered as expected with the exception *C*. *finlaysonianum* and *C*. *madidum*, making it likely that the *ndhJ-K-C* region of these two species was from their plastome.

In contrast, the type II *ndhK* found in *C*. *atropurpureum* was in mitochondrial genome of *C*. *lancifolium* and *C*. *macrorhizon*, so it is likely that type II *ndhJ-K-C* region of *C*. *atropurpureum* was located in the chondriome. Considering the phylogenetic relationship between *C*. *atropurpureum* and *C*. *macrorhizon* [69, 72], the plastid *ndhJ-K-C* region might have been transferred to chondriome in the ancestor of *Cymbidium*. It also seems that the mt-*ndhJ-K-C* region of *C*. *finlaysonianum* and *C*. *madidum* was replaced with its plastid counterpart via recent homologous recombination. As a result, reimported plastid *ndh* genes are derived from the mt-*ndh* copies. The clustering of *ndhG* and *ndhH* among the two organellar genomes in some *Cymbidium* species also supports the hypothesis that their plastid *ndh* genes were relatively recently reimported from chondriome, probably via homologous recombination.

## Materials and methods

### DNA extraction, sequencing, annotation

Fresh leaves of *C*. *finlaysonianum*, *C*. *devonianum* and *Grammatophyllum speciosum* were collected from the orchid collection at the Royal Botanic Gardens, Kew, and Ratcliffe Orchids, Ltd. (Hampshire, UK). Total DNA was extracted by the CTAB method [73]. Except for these three, all other genomic DNAs were taken from DNA Bank at the Royal Botanic Gardens, Kew (Table 3; http://apps.kew.org/dnabank/introduction.html). Vouchers are deposited in the spirit collection at the Royal Botanic Gardens, Kew.

**Table 3:** PCR amplified *ndh* genes among 23 *Cymbidium* species including hybrids and three closely related taxa.

species	region	accession	species	region	accession
*Acriopsis* sp.	ndhB	KX962181	*Cymbidium whiteae*	ndhJKC	KX962256
*Cymbidium atropurpureum*	ndhB	KX962182	*Grammatophyllum speciosum*	ndhJKC	KX962257
*Cymbidium bicolor*	ndhB	KX962183	*Thecostele secunda*	ndhJKC	KX962258
*Cymbidium dayanum*	ndhB	KX962184	*Cymbidium atropurpureum*	ndhJKC TYEP I	KX962234
*Cymbidium devonianum*	ndhB	KX962185	*Cymbidium atropurpureum*	ndhJKC TYEP II	KX962233
*Cymbidium eburneum*	ndhB	KX962186	*Acriopsis* sp.	ndh genes in SSC region	KX962259
*Cymbidium elegans*	ndhB	KX962187	*Cymbidium atropurpureum*	ndh genes in SSC region	KX962260
*Cymbidium erythraeum*	ndhB	KX962188	*Cymbidium bicolor*	ndh genes in SSC region	KX962261
*Cymbidium erythrostylum*	ndhB	KX962189	*Cymbidium dayanum*	ndh genes in SSC region	KX962262
*Cymbidium finlaysonianum*	ndhB	KX962190	*Cymbidium eburneum*	ndh genes in SSC region	KX962263
*Cymbidium floribundum*	ndhB	KX962191	*Cymbidium elegans*	ndh genes in SSC region	KX962264
*Cymbidium giganteum*	ndhB	KX962192	*Cymbidium erythraeum*	ndh genes in SSC region	KX962265
*Cymbidium goeringii*	ndhB	KX962193	*Cymbidium erythrostylum*	ndh genes in SSC region	KX962266
*Cymbidium hookerianum*	ndhB	KX962194	*Cymbidium finlaysonianum*	ndh genes in SSC region	KX962267
*Cymbidium insigne*	ndhB	KX962195	*Cymbidium floribundum*	ndh genes in SSC region	KX962268
*Cymbidium iridioides*	ndhB	KX962196	*Cymbidium giganteum*	ndh genes in SSC region	KX962269
*Cymbidium lowianum*	ndhB	KX962197	*Cymbidium goeringii*	ndh genes in SSC region	KX962270
*Cymbidium madidum*	ndhB	KX962198	*Cymbidium hookerianum*	ndh genes in SSC region	KX962271
*Cymbidium mastersii*	ndhB	KX962199	*Cymbidium insigne*	ndh genes in SSC region	KX962272
*Cymbidium pumilum*	ndhB	KX962200	*Cymbidium iridioides*	ndh genes in SSC region	KX962273
*Cymbidium sanderae*	ndhB	KX962201	*Cymbidium lowianum*	ndh genes in SSC region	KX962274
*Cymbidium suavissimum*	ndhB	KX962202	*Cymbidium madidum*	ndh genes in SSC region	KX962275
*Cymbidium tigrinum*	ndhB	KX962203	*Cymbidium mastersii*	ndh genes in SSC region	KX962276
*Cymbidium whiteae*	ndhB	KX962204	*Cymbidium pumilum*	ndh genes in SSC region	KX962277
*Grammatophyllum speciosum*	ndhB	KX962205	*Cymbidium sanderae*	ndh genes in SSC region	KX962278
*Thecostele secunda*	ndhB	KX962206	*Cymbidium suavissimum*	ndh genes in SSC region	KX962279
*Acriopsis* sp.	ndhD	KX962207	*Cymbidium tigrinum*	ndh genes in SSC region	KX962280
*Cymbidium atropurpureum*	ndhD	KX962208	*Cymbidium whiteae*	ndh genes in SSC region	KX962281
*Cymbidium bicolor*	ndhD	KX962209	*Grammatophyllum speciosum*	ndh genes in SSC region	KX962282
*Cymbidium dayanum*	ndhD	KX962210	*Thecostele secunda*	ndh genes in SSC region	KX962283
*Cymbidium eburneum*	ndhD	KX962211	*Cymbidium bicolor*	ycf1-rpl32_tyep I	KY006886
*Cymbidium elegans*	ndhD	KX962212	*Cymbidium dayanum*	ycf1-rpl32_tyep I	KY006885
*Cymbidium erythraeum*	ndhD	KX962213	*Cymbidium elegans*	ycf1-rpl32_tyep I	KY006884
*Cymbidium erythrostylum*	ndhD	KX962214	*Cymbidium erythraeum*	ycf1-rpl32_tyep I	KY006878
*Cymbidium finlaysonianum*	ndhD	KX962215	*Cymbidium giganteum*	ycf1-rpl32_tyep I	KY006880
*Cymbidium floribundum*	ndhD	KX962216	*Cymbidium insigne*	ycf1-rpl32_tyep I	KY006881
*Cymbidium giganteum*	ndhD	KX962217	*Cymbidium iridioides*	ycf1-rpl32_tyep I	KY006883
*Cymbidium goeringii*	ndhD	KX962218	*Cymbidium lowianum*	ycf1-rpl32_tyep I	KY006882
*Cymbidium hookerianum*	ndhD	KX962219	*Cymbidium mastersii*	ycf1-rpl32_tyep I	KY006888
*Cymbidium insigne*	ndhD	KX962220	*Cymbidium sanderae*	ycf1-rpl32_tyep I	KY006879
*Cymbidium iridioides*	ndhD	KX962221	*Grammatophyllum speciosum*	ycf1-rpl32_tyep I	KY006887
*Cymbidium lowianum*	ndhD	KX962222	*Cymbidium atropurpureum*	ycf1-rpl32_tyep II	KY006898
*Cymbidium madidum*	ndhD	KX962223	*Cymbidium ensifolium*	ycf1-rpl32_tyep II	KY006890
*Cymbidium mastersii*	ndhD	KX962224	*Cymbidium finlaysonianum*	ycf1-rpl32_tyep II	KY006896
*Cymbidium pumilum*	ndhD	KX962225	*Cymbidium floribundum*	ycf1-rpl32_tyep II	KY006899
*Cymbidium sanderae*	ndhD	KX962226	*Cymbidium hookerianum*	ycf1-rpl32_tyep II	KY006892
*Cymbidium suavissimum*	ndhD	KX962227	*Cymbidium kanran*	ycf1-rpl32_tyep II	KY006897
*Cymbidium tigrinum*	ndhD	KX962228	*Cymbidium lancifolium*	ycf1-rpl32_tyep II	KY006889
*Cymbidium whiteae*	ndhD	KX962229	*Cymbidium pumilum*	ycf1-rpl32_tyep II	KY006894
*Grammatophyllum speciosum*	ndhD	KX962230	*Cymbidium sinense*	ycf1-rpl32_tyep II	KY006891
*Thecostele secunda*	ndhD	KX962231	*Cymbidium suavissimum*	ycf1-rpl32_tyep II	KY006893
*Acriopsis* sp.	ndhJKC	KX962232	*Cymbidium tigrinum*	ycf1-rpl32_tyep II	KY006895
*Cymbidium bicolor*	ndhJKC	KX962235	*Acriopsis* sp.	ycf1-rpl32_tyep III	KY006900
*Cymbidium dayanum*	ndhJKC	KX962236	*Cymbidium madidum*	ycf1-rpl32_tyep III	KY006901
*Cymbidium devonianum*	ndhJKC	KX962237	*Thecostele secunda*	ycf1-rpl32_tyep III	KY006902
*Cymbidium eburneum*	ndhJKC	KX962238	*Acriopsis* sp.	ycf1-rpl32_tyep IV	KY006918
*Cymbidium elegans*	ndhJKC	KX962239	*Cymbidium bicolor*	ycf1-rpl32_tyep IV	KY006905
*Cymbidium erythraeum*	ndhJKC	KX962240	*Cymbidium devonianum*	ycf1-rpl32_tyep IV	KY006913
*Cymbidium erythrostylum*	ndhJKC	KX962241	*Cymbidium eburneum*	ycf1-rpl32_tyep IV	KY006915
*Cymbidium finlaysonianum*	ndhJKC	KX962242	*Cymbidium ensifolium*	ycf1-rpl32_tyep IV	KY006904
*Cymbidium floribundum*	ndhJKC	KX962243	*Cymbidium erythrostylum*	ycf1-rpl32_tyep IV	KY006911
*Cymbidium giganteum*	ndhJKC	KX962244	*Cymbidium floribundum*	ycf1-rpl32_tyep IV	KY006908
*Cymbidium goeringii*	ndhJKC	KX962245	*Cymbidium goeringii*	ycf1-rpl32_tyep IV	KY006920
*Cymbidium hookerianum*	ndhJKC	KX962246	*Cymbidium kanran*	ycf1-rpl32_tyep IV	KY006907
*Cymbidium insigne*	ndhJKC	KX962247	*Cymbidium lancifolium*	ycf1-rpl32_tyep IV	KY006919
*Cymbidium iridioides*	ndhJKC	KX962248	*Cymbidium lowianum*	ycf1-rpl32_tyep IV	KY006909
*Cymbidium lowianum*	ndhJKC	KX962249	*Cymbidium mastersii*	ycf1-rpl32_tyep IV	KY006914
*Cymbidium madidum*	ndhJKC	KX962250	*Cymbidium pumilum*	ycf1-rpl32_tyep IV	KY006912
*Cymbidium mastersii*	ndhJKC	KX962251	*Cymbidium sinense*	ycf1-rpl32_tyep IV	KY006906
*Cymbidium pumilum*	ndhJKC	KX962252	*Cymbidium tigrinum*	ycf1-rpl32_tyep IV	KY006903
*Cymbidium sanderae*	ndhJKC	KX962253	*Cymbidium whiteae*	ycf1-rpl32_tyep IV	KY006917
*Cymbidium suavissimum*	ndhJKC	KX962254	*Grammatophyllum speciosum*	ycf1-rpl32_tyep IV	KY006910
*Cymbidium tigrinum*	ndhJKC	KX962255	*Thecostele secunda*	ycf1-rpl32_tyep IV	KY006916

Four regions including all 11 *ndh* genes (*ndhB*, *ndhJ-K-C*, *ndhF*, and *ndhD-E-G-I-A-H*) were assembled from the plastomes of *Cymbidium* [43]. Except for the *ndhF* region, primers were designed for three regions to sequence the full length of each region. In the *ndhF* region, there were a number of homopolymers near both ends. According to previous studies [43] and submitted sequences, this gene was completely deleted in some accessions of *Cymbidium*. Therefore, primers were designed just to confirm absence/presence of *ndhF* in each accession.

The four regions in each species sampled were amplified as follows: 95°C 5min, (95°C 30 sec—50~55°C 30sec– 65~72°C 2min) × 31 cycles, 65~72°C 2min using TaKaRa Premix Taq. PCR products were purified with Qiagen kits using the protocol of the manufacturer and were sequenced using Big-Dye chemistry on an ABI3730XL sequencer following the protocols of the manufacturer. All sequences were assembled by taxon and region using Geneious [74]. We annotated 11 *ndh* genes in each *Cymbidium* and three closely related taxa using complete sequenced plastome sequences in Orchidaceae.

### Detecting *ndh* genes in chondriome

**Fig 6:**
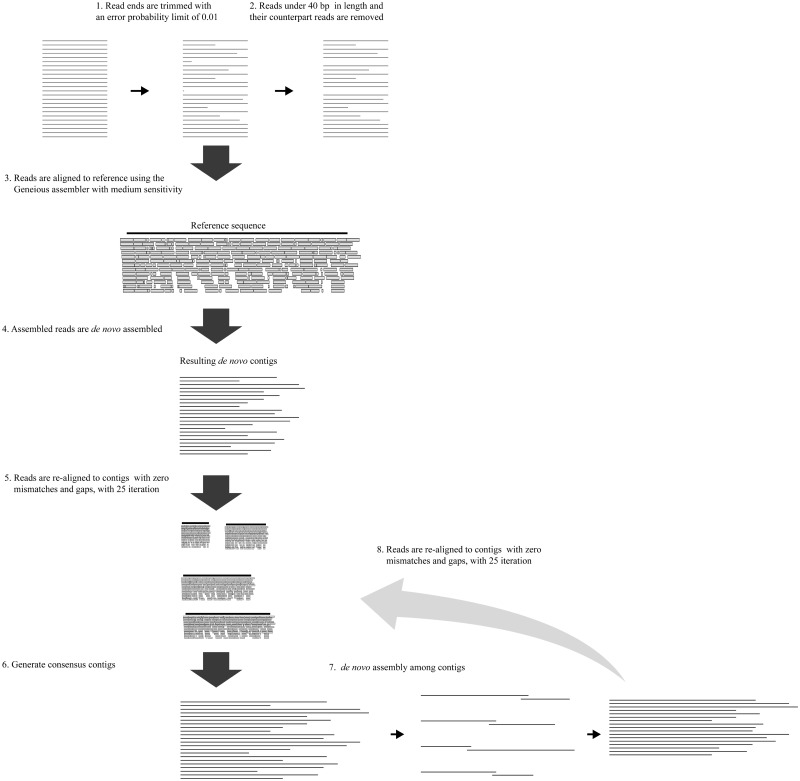
The process of NGS data assembly.

We used the data set from the Sequence Read Archive [75] and *Cymbidium* data generated by Kim (not published) to confirm if *ndh* genes had been translocated to the chondriome (Table 2). We slightly modified the assembly method of Kim et al. [30] (Fig 6). Read ends were trimmed with an error probability limit of 0.01, and then reads under 40 bp and their counterpart reads were removed from data set. Each data set was aligned to the chondriome sequence of *Phoenix dactylifera* [65] under the medium sensitivity option in Geneious [74]. Then, the reads assembled with the reference were extracted and re-assembled using *de novo* assembly in Geneious with zero mismatch and gaps [74]. Several contigs were generated, and reads were re-aligned to them with zero mismatch and gaps with 25 iterations. We generated consensus contigs and aligned them by *de novo* assembly. The resulting contigs were re-used as reference sequences.

Whenever this process was repeated, the number of contigs was reduced, and lengths of resulting contigs extended, and this cycle was repeated until the contigs produced were not extended. To prevent misassembled contigs, only paired reads that matched and upstream or downstream sequence were used throughout the assembly process.

All contigs were investigated for similarity to chondriome sequences using BLAST [76]. Thereafter, mitochondrial contigs were annotated in comparison with their own plastomes. To distinguish the location of genes, genes in the plastome are prefixed with pt- and those in chondriome are prefixed with mt-. Information on mt-*ndh* genes is described in Table 2.

### Phylogenetic analysis of *ndh* genes in both organellar genomes in Orchidaceae

The pt- and mt-*ndh* genes in *Cymbidium* and three closely related taxa were sequenced in this paper. In addition, 55 plastomes (S2 Table) and 38 chondriome sequences (S3 Table) were downloaded from NCBI. The three *Phalaenopsis* plastomes and *Vanilla planifolia* have a 76 ~ 83 bp inversion upstream of the 3′ end of *ndhB*. Each *ndh* gene set was aligned via MAFFT alignment [77].

The *ndhF* gene was excluded from phylogenetic analysis because many species contained two types of *ndhF* genes, and it was difficult to determine where they were located in the organellar genomes. Introns in *ndhA* and *ndhB* were also removed from data set. The best-fit substitution model for each data set was determined using jModeltest2 [78]. Bayesian analysis was performed using mrbayes 3.2.3 [79] as implemented in the CIPRES SCIENCE Gateway [80], under GTR + G model (ngen = 10000000, samplefreq = 1000, burninfrac = 0.25).

## Supporting information

S1 Fig Alignment of *ndh* genes of 23 *Cymbidium* species and three closely related genera. *Masdevallia coccinea ndh* genes were used as reference. A) *ndhB* region. B) *ndhJ-K-C* region. Grey and black in the alignment indicate agreement and disagreement with the consensus sequence, respectively. Red in the alignment indicates ambiguous sites. Black bars at the bottom of the alignment indicate coding regions. Blue arrows and numbers at the bottom of the alignment indicate direct repeat sequences and length of repeat sequence, respectively.
(EPS)

S2 Fig Alignment of *ndh* genes of 23 *Cymbidium* species and three closely related genera. *Masdevallia coccinea ndh* genes were used as reference. A) *ndhD* region. B) *ndhE-G-I-A-H* region Grey and black in the alignment indicate agreement and disagreement to consensus sequence, respectively. Red in the alignment indicates ambiguous sites. Black bars at the bottom of the alignment indicate coding regions. Blue arrows and numbers at the bottom of the alignment indicate direct repeat sequences and length of repeat sequence, respectively. Vertical red dotted lines indicate the end point of deletions. Green and blue lines at the bottom indicate AT- and GC-content of *C*. *elegans*.
(EPS)

S3 Fig Ten gene trees produced by the Bayesian analysis. (EPS)

S4 Fig Gene conversion in the plastid *ndhF* gene. (EPS)

S1 Table The *ndh* status of 743 plastomes. (DOCX)

S2 Table The 55 plastome sequences for phylogenetic study of *ndh* genes. (DOCX)

S3 Table The mt-*ndh* genes for phylogenetic study of *ndh* genes. (DOCX)
