# Supplementary material for: Independent degradation in genes of the plastid ndh gene family in species of the orchid genus Cymbidium (Orchidaceae; Epidendroideae)
Source: PLoS One. 2017 Nov 15;12(11):e0187318. doi: 10.1371/journal.pone.0187318 (PMC5695243; doi:10.1371/journal.pone.0187318)
Supplement: S1 Table — (DOCX) [file pone.0187318.s005.docx]

Table S1. The *ndh* status of 743 plastomes.

| Orders | Species | Accession | *ndhA* | *ndhB* | *ndhC* | *ndhD* | *ndhE* | *ndhF* | *ndhG* | *ndhH* | *ndhI* | *ndhJ* | *ndhK* |
| --- | --- | --- | --- | --- | --- | --- | --- | --- | --- | --- | --- | --- | --- |
| Amborellales | *Amborella trichopoda* | NC_005086 | 1 | 1 | 1 | 1 | 1 | 1 | 1 | 1 | 1 | 1 | 1 |
| Nymphaeales | *Nuphar advena* | NC_008788 | 1 | 1 | 1 | 1 | 1 | 1 | 1 | 1 | 1 | 1 | 1 |
| Nymphaeales | *Nymphaea alba* | NC_006050 | 1 | 1 | 1 | 1 | 1 | 1 | 1 | 1 | 1 | 1 | 1 |
| Nymphaeales | *Nymphaea mexicana* | NC_024542 | 1 | 1 | 1 | 1 | 1 | 1 | 1 | 1 | 1 | 1 | 1 |
| Nymphaeales | *Trithuria inconspicua* | NC_020372 | 1 | 1 | 1 | 1 | 1 | 1 | 1 | 1 | 1 | 1 | 1 |
| Austrobaileyales | *Illicium oligandrum* | NC_009600 | 1 | 1 | 1 | 1 | 1 | 1 | 1 | 1 | 1 | 1 | 1 |
| Chloranthales | *Chloranthus japonicus* | NC_026565 | 1 | 1 | 1 | 1 | 1 | 1 | 1 | 1 | 1 | 1 | 1 |
| Chloranthales | *Chloranthus spicatus* | NC_009598 | 1 | 1 | 1 | 1 | 1 | 1 | 1 | 1 | 1 | 1 | 1 |
| Canellales | *Drimys granadensis* | NC_008456 | 1 | 1 | 1 | 1 | 1 | 1 | 1 | 1 | 1 | 1 | 1 |
| Laurales | *Calycanthus floridus* var*. glaucus* | NC_004993 | 1 | 1 | 1 | 1 | 1 | 1 | 1 | 1 | 1 | 1 | 1 |
| Laurales | *Machilus balansae* | NC_028074 | 1 | 1 | 1 | 1 | 1 | 1 | 1 | 1 | 1 | 1 | 1 |
| Laurales | *Machilus yunnanensis* | NC_028073 | 1 | 1 | 1 | 1 | 1 | 1 | 1 | 1 | 1 | 1 | 1 |
| Magnoliales | *Liriodendron tulipifera* | NC_008326 | 1 | 1 | 1 | 1 | 1 | 1 | 1 | 1 | 1 | 1 | 1 |
| Magnoliales | *Magnolia denudata* | NC_018357 | 1 | 1 | 1 | 1 | 1 | 1 | 1 | 1 | 1 | 1 | 1 |
| Magnoliales | *Magnolia grandiflora* | NC_020318 | 1 | 1 | 1 | 1 | 1 | 1 | 1 | 1 | 1 | 1 | 1 |
| Magnoliales | *Magnolia kwangsiensis* | NC_015892 | 1 | 1 | 1 | 1 | 1 | 1 | 1 | 1 | 1 | 1 | 1 |
| Magnoliales | *Magnolia officinalis* | NC_020316 | 1 | 1 | 1 | 1 | 1 | 1 | 1 | 1 | 1 | 1 | 1 |
| Magnoliales | *Magnolia officinalis* subsp*. biloba* | NC_020317 | 1 | 1 | 1 | 1 | 1 | 1 | 1 | 1 | 1 | 1 | 1 |
| Magnoliales | *Magnolia tripetala* | NC_024027 | 1 | 1 | 1 | 1 | 1 | 1 | 1 | 1 | 1 | 1 | 1 |
| Magnoliales | *Magnolia yunnanensis* | NC_024545 | 1 | 1 | 1 | 1 | 1 | 1 | 1 | 1 | 1 | 1 | 1 |
| Piperales | *Piper cenocladum* | NC_008457 | 1 | 1 | 1 | 1 | 1 | 1 | 1 | 1 | 1 | 1 | 1 |
| Piperales | *Piper kadsura* | NC_027941 | 1 | 1 | 1 | 1 | 1 | 1 | 1 | 1 | 1 | 1 | 1 |
| Ceratophyllales | *Ceratophyllum demersum* | NC_009962 | 1 | 1 | 1 | 1 | 1 | 1 | 1 | 1 | 1 | 1 | 1 |
| Acorales | *Acorus americanus* | NC_010093 | 1 | 1 | 1 | 1 | 1 | 1 | 1 | 1 | 1 | 1 | 1 |
| Acorales | *Acorus calamus* | NC_007407 | 1 | 1 | 1 | 1 | 1 | 1 | 1 | 1 | 1 | 1 | 1 |
| Acorales | *Acorus gramineus* | NC_026299 | 1 | 1 | 1 | 1 | 1 | 1 | 1 | 1 | 1 | 1 | 1 |
| Alismatales | *Colocasia esculenta* | NC_016753 | 1 | 1 | 1 | 1 | 1 | 1 | 1 | 1 | 1 | 1 | 1 |
| Alismatales | *Dieffenbachia seguine* | NC_027272 | 1 | 1 | 1 | 1 | 1 | 1 | 1 | 1 | 1 | 1 | 1 |
| Alismatales | *Elodea canadensis* | NC_018541 | 1 | 1 | 1 | 1 | 1 | 1 | 1 | 1 | 1 | 1 | 1 |
| Alismatales | *Epipremnum aureum* | NC_027954 | 1 | 1 | 1 | 1 | 1 | 1 | 1 | 1 | 1 | 1 | 1 |
| Alismatales | *Lemna minor* | NC_010109 | 1 | 1 | 1 | 1 | 1 | 1 | 1 | 1 | 1 | 1 | 1 |
| Alismatales | *Najas flexilis* | NC_021936 | 4 | 2 | 2 | 3 | 2 | 4 | 4 | 3 | 4 | 4 | 4 |
| Alismatales | *Pinellia ternata* | NC_027681 | 1 | 1 | 1 | 1 | 1 | 1 | 1 | 1 | 1 | 1 | 1 |
| Alismatales | *Spirodela polyrhiza* | NC_015891 | 1 | 1 | 1 | 1 | 1 | 1 | 1 | 1 | 1 | 1 | 1 |
| Alismatales | *Wolffia australiana* | NC_015899 | 1 | 1 | 1 | 1 | 1 | 1 | 1 | 1 | 1 | 1 | 1 |
| Alismatales | *Wolffiella lingulata* | NC_015894 | 1 | 1 | 1 | 1 | 1 | 1 | 1 | 1 | 1 | 1 | 1 |
| Petrosaviales | *Petrosavia stellaris* | NC_023356 | 3 | 2 | 3 | 3 | 3 | 4 | 3 | 2 | 4 | 4 | 4 |
| Dioscoreales | *Dioscorea elephantipes* | NC_009601 | 1 | 1 | 1 | 1 | 1 | 1 | 1 | 1 | 1 | 1 | 1 |
| Dioscoreales | *Dioscorea rotundata* | NC_024170 | 1 | 1 | 1 | 1 | 1 | 1 | 1 | 1 | 1 | 1 | 1 |
| Dioscoreales | *Dioscorea zingiberensis* | NC_027090 | 1 | 1 | 1 | 1 | 1 | 1 | 1 | 1 | 1 | 1 | 1 |
| Pandanales | *Carludovica palmata* | NC_026786 | 1 | 1 | 1 | 1 | 1 | 1 | 1 | 1 | 1 | 1 | 1 |
| Pandanales | *Sciaphila densiflora* | NC_027659 | 4 | 4 | 4 | 4 | 4 | 4 | 4 | 4 | 4 | 4 | 4 |
| Liliales | *Alstroemeria aurea* | KC968976 | 1 | 1 | 1 | 1 | 1 | 1 | 1 | 1 | 1 | 1 | 1 |
| Liliales | *Bomarea edulis* | NC_025306 | 1 | 1 | 1 | 1 | 1 | 1 | 1 | 1 | 1 | 1 | 1 |
| Liliales | *Campynema lineare* | NC_026785 | 1 | 1 | 1 | 1 | 1 | 1 | 1 | 1 | 1 | 1 | 1 |
| Liliales | *Fritillaria cirrhosa* | NC_024728 | 1 | 1 | 1 | 1 | 1 | 1 | 1 | 1 | 1 | 1 | 1 |
| Liliales | *Fritillaria hupehensis* | NC_024736 | 1 | 1 | 1 | 1 | 1 | 1 | 1 | 1 | 1 | 1 | 1 |
| Liliales | *Fritillaria taipaiensis* | NC_023247 | 1 | 1 | 1 | 1 | 1 | 1 | 1 | 1 | 1 | 1 | 1 |
| Liliales | *Heloniopsis tubiflora* | NC_027159 | 1 | 1 | 1 | 1 | 1 | 1 | 1 | 1 | 1 | 1 | 1 |
| Liliales | *Lilium hansonii* | NC_027674 | 1 | 1 | 1 | 1 | 1 | 1 | 1 | 1 | 1 | 1 | 1 |
| Liliales | *Lilium longiflorum* | KC968977 | 1 | 1 | 1 | 1 | 1 | 1 | 1 | 1 | 1 | 1 | 1 |
| Liliales | *Lilium sp.* | NC_027679 | 1 | 1 | 1 | 1 | 1 | 1 | 1 | 1 | 1 | 1 | 1 |
| Liliales | *Lilium superbum* | NC_026787 | 1 | 1 | 1 | 1 | 1 | 1 | 1 | 1 | 1 | 1 | 1 |
| Liliales | *Lilium tsingtauense* | NC_027675 | 1 | 1 | 1 | 1 | 1 | 1 | 1 | 1 | 1 | 1 | 1 |
| Liliales | *Luzuriaga radicans* | NC_025333 | 1 | 1 | 1 | 1 | 1 | 1 | 1 | 1 | 1 | 1 | 1 |
| Liliales | *Paris verticillata* | NC_024560 | 1 | 1 | 1 | 1 | 1 | 1 | 1 | 1 | 1 | 1 | 1 |
| Liliales | *Smilax china* | HM536959 | 1 | 1 | 1 | 1 | 1 | 1 | 1 | 1 | 1 | 1 | 1 |
| Liliales | *Trillium cuneatum* | NC_027185 | 1 | 1 | 1 | 1 | 1 | 1 | 1 | 1 | 1 | 1 | 1 |
| Liliales | *Trillium decumbens* | NC_027282 | 1 | 1 | 1 | 1 | 1 | 1 | 1 | 1 | 1 | 1 | 1 |
| Liliales | *Trillium maculatum* | NC_027738 | 1 | 1 | 1 | 1 | 1 | 1 | 1 | 1 | 1 | 1 | 1 |
| Liliales | *Trillium tschonoskii* | NC_027739 | 1 | 1 | 1 | 1 | 1 | 1 | 1 | 1 | 1 | 1 | 1 |
| Liliales | *Veratrum patulum* | NC_022715 | 1 | 1 | 1 | 1 | 1 | 1 | 1 | 1 | 1 | 1 | 1 |
| Liliales | *Xerophyllum tenax* | NC_027158 | 1 | 1 | 1 | 1 | 1 | 1 | 1 | 1 | 1 | 1 | 1 |
| Asparagales | *Allium cepa* | NC_024813 | 1 | 1 | 1 | 1 | 1 | 1 | 1 | 1 | 1 | 1 | 1 |
| Asparagales | *Bletilla ochracea* | NC_029483 | 2 | 1 | 1 | 2 | 1 | 2 | 2 | 2 | 2 | 1 | 2 |
| Asparagales | *Bletilla striata* | NC_028422 | 2 | 3 | 3 | 2 | 1 | 2 | 1 | 3 | 2 | 1 | 3 |
| Asparagales | *Calanthe triplicata* | NC_024544 | 1 | 1 | 1 | 1 | 1 | 1 | 1 | 1 | 1 | 1 | 1 |
| Asparagales | *Cattleya crispata* | NC_026568 | 4 | 2 | 1 | 2 | 1 | 4 | 3 | 3 | 4 | 3 | 3 |
| Asparagales | *Corallorhiza bulbosa* | NC_025659 | 3 | 3 | 2 | 2 | 4 | 4 | 4 | 2 | 2 | 1 | 3 |
| Asparagales | *Corallorhiza macrantha* | NC_025660 | 3 | 3 | 2 | 2 | 4 | 4 | 4 | 2 | 2 | 2 | 2 |
| Asparagales | *Corallorhiza mertensiana* | NC_025661 | 3 | 3 | 2 | 2 | 4 | 4 | 3 | 3 | 2 | 2 | 2 |
| Asparagales | *Corallorhiza odontorhiza* | NC_025664 | 3 | 3 | 2 | 2 | 4 | 4 | 3 | 3 | 2 | 4 | 3 |
| Asparagales | *Corallorhiza striata* var*. vreelandii* | JX087681 | 3 | 2 | 2 | 3 | 3 | 4 | 2 | 4 | 2 | 2 | 3 |
| Asparagales | *Corallorhiza trifida* | NC_025662 | 3 | 2 | 2 | 3 | 4 | 4 | 3 | 2 | 3 | 3 | 3 |
| Asparagales | *Corallorhiza wisteriana* | NC_025663 | 3 | 2 | 2 | 3 | 4 | 4 | 3 | 3 | 2 | 4 | 3 |
| Asparagales | *Cymbidium aloifolium* | NC_021429 | 2 | 2 | 2 | 2 | 1 | 2 | 1 | 2 | 2 | 1 | 2 |
| Asparagales | *Cymbidium ensifolium* 1 | KU179434 | 3 | 2 | 1 | 2 | 1 | 4 | 2 | 4 | 1 | 4 | 2 |
| Asparagales | *Cymbidium ensifolium* 2 | NC_028525 | 1 | 2 | 1 | 2 | 1 | 2 | 2 | 2 | 1 | 1 | 2 |
| Asparagales | *Cymbidium faberi* | NC_027743 | 2 | 2 | 1 | 2 | 1 | 2 | 2 | 2 | 1 | 1 | 2 |
| Asparagales | *Cymbidium goeringii* | NC_028524 | 2 | 2 | 1 | 2 | 1 | 2 | 1 | 2 | 1 | 1 | 2 |
| Asparagales | *Cymbidium kanran* | KU179435 | 3 | 2 | 1 | 2 | 1 | 4 | 2 | 4 | 1 | 1 | 2 |
| Asparagales | *Cymbidium lancifolium* | KU179436 | 3 | 2 | 1 | 2 | 2 | 4 | 2 | 4 | 2 | 2 | 2 |
| Asparagales | *Cymbidium macrorhizon* | KU179437 | 3 | 2 | 1 | 2 | 2 | 4 | 2 | 4 | 2 | 2 | 2 |
| Asparagales | *Cymbidium mannii* | NC_021433 | 2 | 3 | 3 | 2 | 1 | 2 | 1 | 3 | 1 | 1 | 2 |
| Asparagales | *Cymbidium sinense* | NC_021430 | 2 | 2 | 1 | 2 | 1 | 2 | 2 | 2 | 1 | 1 | 2 |
| Asparagales | *Cymbidium tortisepalum* | NC_021431 | 2 | 2 | 1 | 2 | 1 | 2 | 2 | 2 | 1 | 1 | 2 |
| Asparagales | *Cymbidium tracyanum* | NC_021432 | 1 | 2 | 1 | 1 | 1 | 2 | 1 | 1 | 2 | 1 | 2 |
| Asparagales | *Cypripedium formosanum* | NC_026772 | 1 | 1 | 1 | 1 | 1 | 1 | 1 | 1 | 1 | 1 | 1 |
| Asparagales | *Cypripedium japonicum* | NC_027227 | 1 | 1 | 1 | 1 | 1 | 1 | 1 | 1 | 1 | 1 | 1 |
| Asparagales | *Cypripedium macranthos* | NC_024421 | 1 | 1 | 1 | 1 | 1 | 1 | 1 | 1 | 1 | 1 | 1 |
| Asparagales | *Dendrobium catenatum* | NC_024019 | 3 | 1 | 4 | 3 | 1 | 3 | 3 | 2 | 4 | 2 | 4 |
| Asparagales | *Dendrobium chrysotoxum* | NC_028549 | 3 | 1 | 4 | 3 | 1 | 3 | 3 | 1 | 4 | 1 | 4 |
| Asparagales | *Dendrobium huoshanense* | NC_028430 | 3 | 1 | 4 | 3 | 2 | 3 | 3 | 2 | 4 | 2 | 4 |
| Asparagales | *Dendrobium nobile* | NC_029456 | 3 | 1 | 4 | 3 | 2 | 3 | 3 | 2 | 4 | 2 | 4 |
| Asparagales | *Dendrobium pendulum* | NC_029705 | 3 | 1 | 4 | 3 | 1 | 3 | 3 | 1 | 4 | 1 | 3 |
| Asparagales | *Dendrobium strongylanthum* | NC_027691 | 3 | 1 | 4 | 3 | 1 | 3 | 3 | 1 | 4 | 2 | 3 |
| Asparagales | *Elleanthus sodiroi* | NC_027266 | 1 | 1 | 1 | 1 | 1 | 1 | 1 | 1 | 1 | 1 | 1 |
| Asparagales | *Epipogium aphyllum* | NC_026449 | 4 | 4 | 4 | 4 | 4 | 4 | 4 | 4 | 4 | 4 | 4 |
| Asparagales | *Epipogium roseum* | NC_026448 | 4 | 4 | 4 | 4 | 4 | 4 | 4 | 4 | 4 | 4 | 4 |
| Asparagales | *Erycina pusilla* | NC_018114 | 4 | 3 | 2 | 3 | 3 | 4 | 3 | 3 | 4 | 3 | 3 |
| Asparagales | *Eustrephus latifolius* | NC_025305 | 1 | 1 | 1 | 1 | 1 | 1 | 1 | 1 | 1 | 1 | 1 |
| Asparagales | *Goodyera fumata* | NC_026773 | 1 | 1 | 1 | 1 | 1 | 1 | 1 | 1 | 1 | 1 | 1 |
| Asparagales | *Goodyera procera* | NC_029363 | 1 | 1 | 1 | 1 | 1 | 2 | 1 | 1 | 1 | 1 | 1 |
| Asparagales | *Goodyera schlechtendaliana* | NC_029364 | 1 | 1 | 1 | 1 | 1 | 1 | 1 | 1 | 1 | 1 | 1 |
| Asparagales | *Goodyera velutina* | NC_029365 | 1 | 1 | 1 | 3 | 1 | 1 | 1 | 1 | 1 | 1 | 1 |
| Asparagales | *Habenaria pantlingiana* | NC_026775 | 1 | 1 | 1 | 1 | 1 | 1 | 1 | 1 | 1 | 1 | 1 |
| Asparagales | *Iris gatesii* | NC_024936 | 1 | 1 | 1 | 1 | 1 | 1 | 1 | 1 | 1 | 1 | 1 |

Supplementary table S1. Continue

| Order | Species | Accession | *ndhA* | *ndhB* | *ndhC* | *ndhD* | *ndhE* | *ndhF* | *ndhG* | *ndhH* | *ndhI* | *ndhJ* | *ndhK* |
| --- | --- | --- | --- | --- | --- | --- | --- | --- | --- | --- | --- | --- | --- |
| Asparagales | *Masdevallia coccinea* | NC_026541 | 1 | 1 | 1 | 1 | 1 | 1 | 1 | 1 | 1 | 1 | 1 |
| Asparagales | *Masdevallia picturata* | NC_026777 | 1 | 1 | 1 | 1 | 1 | 1 | 1 | 1 | 1 | 1 | 1 |
| Asparagales | *Neottia nidus-avis* | NC_016471 | 4 | 3 | 3 | 4 | 4 | 4 | 4 | 3 | 4 | 3 | 4 |
| Asparagales | *Oncidium* Gower Ramsey | NC_014056 | 3 | 2 | 2 | 3 | 1 | 4 | 2 | 3 | 3 | 3 | 3 |
| Asparagales | *Oncidium sphacelatum* | NC_028148 | 3 | 2 | 2 | 3 | 1 | 4 | 2 | 3 | 3 | 3 | 3 |
| Asparagales | *Paphiopedilum armeniacum* | NC_026779 | 4 | 2 | 2 | 3 | 4 | 4 | 4 | 4 | 4 | 2 | 2 |
| Asparagales | *Paphiopedilum niveum* | NC_026776 | 4 | 2 | 2 | 3 | 4 | 4 | 4 | 4 | 4 | 2 | 2 |
| Asparagales | *Phalaenopsis aphrodite* subsp*. formosana* | NC_007499 | 4 | 2 | 2 | 3 | 3 | 4 | 3 | 4 | 3 | 2 | 3 |
| Asparagales | *Phalaenopsis equestris* | NC_017609 | 4 | 2 | 2 | 3 | 4 | 4 | 3 | 4 | 3 | 2 | 3 |
| Asparagales | *Phalaenopsis* hybrid cultivar | NC_025593 | 4 | 2 | 2 | 3 | 3 | 4 | 3 | 4 | 3 | 2 | 3 |
| Asparagales | *Phragmipedium longifolium* | NC_028149 | 3 | 3 | 4 | 2 | 4 | 4 | 4 | 4 | 3 | 2 | 3 |
| Asparagales | *Polygonatum cyrtonema* | NC_028429 | 1 | 1 | 1 | 1 | 1 | 1 | 1 | 1 | 1 | 1 | 1 |
| Asparagales | *Polygonatum verticillatum* | NC_028523 | 1 | 1 | 1 | 1 | 1 | 1 | 1 | 1 | 1 | 1 | 1 |
| Asparagales | *Rhizanthella gardneri* | NC_014874 | 4 | 4 | 4 | 4 | 4 | 4 | 4 | 4 | 4 | 3 | 3 |
| Asparagales | *Sobralia aff. bouchei* | NC_028209 | 1 | 1 | 1 | 1 | 1 | 1 | 1 | 1 | 1 | 1 | 1 |
| Asparagales | *Sobralia callosa* | NC_028147 | 1 | 1 | 1 | 1 | 1 | 1 | 1 | 1 | 1 | 1 | 1 |
| Asparagales | *Vanilla planifolia* | NC_026778 | 4 | 3 | 4 | 4 | 4 | 4 | 4 | 4 | 4 | 4 | 4 |
| Commelinids incertae sedis | *Dasypogon bromeliifolius* | NC_020367 | 1 | 1 | 1 | 1 | 1 | 1 | 1 | 1 | 1 | 1 | 1 |
| Arecales | *Bismarckia nobilis* | NC_020366 | 1 | 1 | 1 | 1 | 1 | 1 | 1 | 1 | 1 | 1 | 1 |
| Arecales | *Calamus caryotoides* | NC_020365 | 1 | 1 | 1 | 1 | 1 | 1 | 1 | 1 | 1 | 1 | 1 |
| Arecales | *Cocos nucifera* | NC_022417 | 1 | 1 | 1 | 1 | 1 | 1 | 1 | 1 | 1 | 1 | 1 |
| Arecales | *Colpothrinax cookii* | NC_028026 | 1 | 1 | 1 | 1 | 1 | 1 | 1 | 1 | 1 | 1 | 1 |
| Arecales | *Elaeis guineensis* | NC_017602 | 1 | 1 | 1 | 1 | 1 | 1 | 1 | 1 | 1 | 1 | 1 |
| Arecales | *Phoenix dactylifera* | NC_013991 | 1 | 1 | 1 | 1 | 1 | 1 | 1 | 1 | 1 | 1 | 1 |
| Arecales | *Podococcus barteri* | NC_027276 | 1 | 1 | 1 | 1 | 1 | 1 | 1 | 1 | 1 | 1 | 1 |
| Arecales | *Pseudophoenix vinifera* | NC_020364 | 1 | 1 | 1 | 1 | 1 | 1 | 1 | 1 | 1 | 1 | 1 |
| Arecales | *Sabal domingensis* | NC_026444 | 1 | 1 | 1 | 1 | 1 | 1 | 1 | 1 | 1 | 1 | 1 |
| Poales | *Acidosasa purpurea* | NC_015820 | 1 | 1 | 1 | 1 | 1 | 1 | 1 | 1 | 1 | 1 | 1 |
| Poales | *Aegilops bicornis* | NC_024831 | 1 | 1 | 1 | 1 | 1 | 1 | 1 | 1 | 1 | 1 | 1 |
| Poales | *Aegilops cylindrica* | NC_023096 | 1 | 1 | 1 | 1 | 1 | 1 | 1 | 1 | 1 | 1 | 1 |
| Poales | *Aegilops geniculata* | NC_023097 | 1 | 1 | 1 | 1 | 1 | 1 | 1 | 1 | 1 | 1 | 1 |
| Poales | *Aegilops kotschyi* | NC_024832 | 1 | 1 | 1 | 1 | 1 | 1 | 1 | 1 | 1 | 1 | 1 |
| Poales | *Aegilops longissima* | NC_024830 | 1 | 1 | 1 | 1 | 1 | 1 | 1 | 1 | 1 | 1 | 1 |
| Poales | *Aegilops searsii* | NC_024815 | 1 | 1 | 1 | 1 | 1 | 1 | 1 | 1 | 1 | 1 | 1 |
| Poales | *Aegilops sharonensis* | NC_024816 | 1 | 1 | 1 | 1 | 1 | 1 | 1 | 1 | 1 | 1 | 1 |
| Poales | *Aegilops speltoides* | NC_022135 | 1 | 1 | 1 | 1 | 1 | 1 | 1 | 1 | 1 | 1 | 1 |
| Poales | *Aegilops tauschii* | NC_022133 | 1 | 1 | 1 | 1 | 1 | 1 | 1 | 1 | 1 | 1 | 1 |
| Poales | *Agrostis stolonifera* | NC_008591 | 1 | 1 | 1 | 1 | 1 | 1 | 1 | 1 | 1 | 1 | 1 |
| Poales | *Alloteropsis angusta* | NC_027951 | 1 | 1 | 1 | 1 | 1 | 1 | 1 | 1 | 1 | 1 | 1 |
| Poales | *Alloteropsis cimicina* | NC_027952 | 1 | 1 | 1 | 1 | 1 | 1 | 1 | 1 | 1 | 1 | 1 |
| Poales | *Alloteropsis semialata* | NC_027824 | 1 | 1 | 1 | 1 | 1 | 1 | 1 | 1 | 1 | 1 | 1 |
| Poales | *Ammophila breviligulata* | NC_027465 | 1 | 1 | 1 | 1 | 1 | 1 | 1 | 1 | 1 | 1 | 1 |
| Poales | *Ampelocalamus calcareus* | NC_024731 | 1 | 1 | 1 | 1 | 1 | 1 | 1 | 1 | 1 | 1 | 1 |
| Poales | *Ampelodesmos mauritanicus* | NC_027466 | 1 | 1 | 1 | 1 | 1 | 1 | 1 | 1 | 1 | 1 | 1 |
| Poales | *Ananas comosus* | NC_026220 | 1 | 1 | 1 | 1 | 1 | 1 | 1 | 1 | 1 | 1 | 1 |
| Poales | *Anomochloa marantoidea* | NC_014062 | 1 | 1 | 1 | 1 | 1 | 1 | 1 | 1 | 1 | 1 | 1 |
| Poales | *Anthoxanthum nitens* | NC_027475 | 1 | 1 | 1 | 1 | 1 | 1 | 1 | 1 | 1 | 1 | 1 |
| Poales | *Anthoxanthum odoratum* | NC_027467 | 1 | 1 | 1 | 1 | 1 | 1 | 1 | 1 | 1 | 1 | 1 |
| Poales | *Aristida purpurea* | NC_025228 | 1 | 1 | 1 | 1 | 1 | 1 | 1 | 1 | 1 | 1 | 1 |
| Poales | *Arundinaria appalachiana* | NC_023934 | 1 | 1 | 1 | 1 | 1 | 1 | 1 | 1 | 1 | 1 | 1 |
| Poales | *Arundinaria fargesii* | NC_024712 | 1 | 1 | 1 | 1 | 1 | 1 | 1 | 1 | 1 | 1 | 1 |
| Poales | *Arundinaria gigantea* | NC_020341 | 1 | 1 | 1 | 1 | 1 | 1 | 1 | 1 | 1 | 1 | 1 |
| Poales | *Arundinaria tecta* | NC_023935 | 1 | 1 | 1 | 1 | 1 | 1 | 1 | 1 | 1 | 1 | 1 |
| Poales | *Avena sativa* | NC_027468 | 1 | 1 | 1 | 1 | 1 | 1 | 1 | 1 | 1 | 1 | 1 |
| Poales | *Bambusa arnhemica* | NC_026958 | 1 | 1 | 1 | 1 | 1 | 1 | 1 | 1 | 1 | 1 | 1 |
| Poales | *Bambusa bambos* | NC_026957 | 1 | 1 | 1 | 1 | 1 | 1 | 1 | 1 | 1 | 1 | 1 |
| Poales | *Bambusa emeiensis* | NC_015830 | 1 | 1 | 1 | 1 | 1 | 1 | 1 | 1 | 1 | 1 | 1 |
| Poales | *Bambusa multiplex* | NC_024668 | 1 | 1 | 1 | 1 | 1 | 1 | 1 | 1 | 1 | 1 | 1 |
| Poales | *Brachyelytrum aristosum* | NC_027470 | 1 | 1 | 1 | 1 | 1 | 1 | 1 | 1 | 1 | 1 | 1 |
| Poales | *Brachypodium distachyon* | NC_011032 | 1 | 1 | 1 | 1 | 1 | 1 | 1 | 1 | 1 | 1 | 1 |
| Poales | *Briza maxima* | NC_027471 | 1 | 1 | 1 | 1 | 1 | 1 | 1 | 1 | 1 | 1 | 1 |
| Poales | *Bromus vulgaris* | NC_027472 | 1 | 1 | 1 | 1 | 1 | 1 | 1 | 1 | 1 | 1 | 1 |
| Poales | *Buergersiochloa bambusoides* | NC_026968 | 1 | 1 | 1 | 1 | 1 | 1 | 1 | 1 | 1 | 1 | 1 |
| Poales | *Carex siderosticta* | NC_027250 | 1 | 1 | 1 | 1 | 1 | 1 | 1 | 1 | 1 | 1 | 1 |
| Poales | *Cenchrus americanus* | NC_024171 | 1 | 1 | 1 | 1 | 1 | 1 | 1 | 1 | 1 | 1 | 1 |
| Poales | *Centotheca lappacea* | NC_025229 | 1 | 1 | 1 | 1 | 1 | 1 | 1 | 1 | 1 | 1 | 1 |
| Poales | *Chikusichloa aquatica* | NC_027184 | 1 | 1 | 1 | 1 | 1 | 1 | 1 | 1 | 1 | 1 | 1 |
| Poales | *Chimonocalamus longiusculus* | NC_024714 | 1 | 1 | 1 | 1 | 1 | 1 | 1 | 1 | 1 | 1 | 1 |
| Poales | *Chionochloa macra* | NC_025230 | 1 | 1 | 1 | 1 | 1 | 1 | 1 | 1 | 1 | 1 | 1 |
| Poales | *Chusquea circinata* | NC_027490 | 1 | 1 | 1 | 1 | 1 | 1 | 1 | 1 | 1 | 1 | 1 |
| Poales | *Chusquea liebmannii* | NC_026969 | 1 | 1 | 1 | 1 | 1 | 1 | 1 | 1 | 1 | 1 | 1 |
| Poales | *Chusquea spectabilis* | NC_026959 | 1 | 1 | 1 | 1 | 1 | 1 | 1 | 1 | 1 | 1 | 1 |
| Poales | *Coix lacryma-jobi* | NC_013273 | 2 | 1 | 1 | 1 | 1 | 1 | 1 | 1 | 1 | 1 | 1 |
| Poales | *Coleataenia prionitis* | NC_025231 | 1 | 1 | 1 | 1 | 1 | 1 | 1 | 1 | 1 | 1 | 1 |
| Poales | *Dactylis glomerata* | NC_027473 | 1 | 1 | 1 | 1 | 1 | 1 | 1 | 1 | 1 | 1 | 1 |
| Poales | *Danthonia californica* | NC_025232 | 1 | 1 | 1 | 1 | 1 | 1 | 1 | 1 | 1 | 1 | 1 |
| Poales | *Dendrocalamus latiflorus* | NC_013088 | 1 | 1 | 1 | 1 | 1 | 1 | 1 | 1 | 1 | 1 | 1 |
| Poales | *Deschampsia antarctica* | NC_023533 | 1 | 1 | 1 | 1 | 1 | 1 | 1 | 1 | 1 | 1 | 1 |
| Poales | *Diandrolyra* sp. | NC_026960 | 1 | 1 | 1 | 1 | 1 | 1 | 1 | 1 | 1 | 1 | 1 |
| Poales | *Diarrhena obovata* | NC_027474 | 1 | 1 | 1 | 1 | 1 | 1 | 1 | 1 | 1 | 1 | 1 |
| Poales | *Digitaria exilis* | NC_024176 | 1 | 1 | 1 | 1 | 1 | 1 | 1 | 1 | 1 | 1 | 1 |
| Poales | *Echinochloa crus-galli* | NC_028719 | 1 | 1 | 1 | 1 | 1 | 1 | 1 | 1 | 1 | 1 | 1 |
| Poales | *Echinochloa oryzicola* | NC_024643 | 1 | 1 | 1 | 1 | 1 | 1 | 1 | 1 | 1 | 1 | 1 |
| Poales | *Elytrophorus spicatus* | NC_025233 | 1 | 1 | 1 | 1 | 1 | 1 | 1 | 1 | 1 | 1 | 1 |
| Poales | *Eriachne stipacea* | NC_025234 | 1 | 1 | 1 | 1 | 1 | 1 | 1 | 1 | 1 | 1 | 1 |
| Poales | *Fargesia nitida* | NC_024715 | 1 | 1 | 1 | 1 | 1 | 1 | 1 | 1 | 1 | 1 | 1 |
| Poales | *Fargesia spathacea* | NC_024716 | 1 | 1 | 1 | 1 | 1 | 1 | 1 | 1 | 1 | 1 | 1 |
| Poales | *Fargesia yunnanensis* | NC_024717 | 1 | 1 | 1 | 1 | 1 | 1 | 1 | 1 | 1 | 1 | 1 |
| Poales | *Ferrocalamus rimosivaginus* | NC_015831 | 1 | 1 | 1 | 1 | 1 | 1 | 1 | 1 | 1 | 1 | 1 |
| Poales | *Festuca altissima* | NC_019648 | 1 | 1 | 1 | 1 | 1 | 1 | 1 | 1 | 1 | 1 | 1 |
| Poales | *Festuca arundinacea* | NC_011713 | 2 | 1 | 1 | 1 | 1 | 1 | 1 | 1 | 1 | 1 | 1 |
| Poales | *Festuca ovina* | NC_019649 | 1 | 1 | 1 | 1 | 1 | 1 | 1 | 1 | 1 | 1 | 1 |
| Poales | *Festuca pratensis* | NC_019650 | 1 | 1 | 1 | 1 | 1 | 1 | 1 | 1 | 1 | 1 | 1 |
| Poales | *Gaoligongshania megalothyrsa* | NC_024718 | 1 | 1 | 1 | 1 | 1 | 1 | 1 | 1 | 1 | 1 | 1 |
| Poales | *Gelidocalamus tessellatus* | NC_024719 | 1 | 1 | 1 | 1 | 1 | 1 | 1 | 1 | 1 | 1 | 1 |
| Poales | *Greslania* sp. | NC_026961 | 1 | 1 | 1 | 1 | 1 | 1 | 1 | 1 | 1 | 1 | 1 |
| Poales | *Guadua weberbaueri* | NC_026991 | 1 | 1 | 1 | 1 | 1 | 1 | 1 | 1 | 1 | 1 | 1 |
| Poales | *Hakonechloa macra* | NC_025235 | 1 | 1 | 1 | 1 | 1 | 1 | 1 | 1 | 1 | 1 | 1 |
| Poales | *Helictochloa hookeri* | NC_027469 | 1 | 1 | 1 | 1 | 1 | 1 | 1 | 1 | 1 | 1 | 1 |
| Poales | *Hickelia madagascariensis* | NC_026962 | 1 | 1 | 1 | 1 | 1 | 1 | 1 | 1 | 1 | 1 | 1 |
| Poales | *Hordeum jubatum* | NC_027476 | 1 | 1 | 1 | 1 | 1 | 1 | 1 | 1 | 1 | 1 | 1 |
| Poales | *Hordeum vulgare* subsp*. vulgare* | NC_008590 | 1 | 1 | 1 | 1 | 1 | 1 | 1 | 1 | 1 | 1 | 1 |
| Poales | *Indocalamus longiauritus* | NC_015803 | 1 | 1 | 1 | 1 | 1 | 1 | 1 | 1 | 1 | 1 | 1 |
| Poales | *Indocalamus wilsonii* | NC_024720 | 1 | 1 | 1 | 1 | 1 | 1 | 1 | 1 | 1 | 1 | 1 |
| Poales | *Indosasa sinica* | NC_024721 | 1 | 1 | 1 | 1 | 1 | 1 | 1 | 1 | 1 | 1 | 1 |
| Poales | *Isachne distichophylla* | NC_025236 | 1 | 1 | 1 | 1 | 1 | 1 | 1 | 1 | 1 | 1 | 1 |
| Poales | *Lecomtella madagascariensis* | NC_024106 | 1 | 1 | 1 | 1 | 1 | 1 | 1 | 1 | 1 | 1 | 1 |

Supplementary table S1. Continues

| Order | Species | Accession | *ndhA* | *ndhB* | *ndhC* | *ndhD* | *ndhE* | *ndhF* | *ndhG* | *ndhH* | *ndhI* | *ndhJ* | *ndhK* |
| --- | --- | --- | --- | --- | --- | --- | --- | --- | --- | --- | --- | --- | --- |
| Poales | *Leersia tisserantii* | NC_016677 | 1 | 1 | 1 | 1 | 1 | 1 | 1 | 1 | 1 | 1 | 1 |
| Poales | *Lithachne pauciflora* | NC_026970 | 1 | 1 | 1 | 1 | 1 | 1 | 1 | 1 | 1 | 1 | 1 |
| Poales | *Lolium multiflorum* | NC_019651 | 1 | 1 | 1 | 1 | 1 | 1 | 1 | 1 | 1 | 1 | 1 |
| Poales | *Lolium perenne* | NC_009950 | 1 | 1 | 1 | 1 | 1 | 1 | 1 | 1 | 1 | 1 | 1 |
| Poales | *Melica mutica* | NC_027477 | 1 | 1 | 1 | 1 | 1 | 1 | 1 | 1 | 1 | 1 | 1 |
| Poales | *Melica subulata* | NC_027478 | 1 | 1 | 1 | 1 | 1 | 1 | 1 | 1 | 1 | 1 | 1 |
| Poales | *Miscanthus sacchariflorus* | NC_028720 | 1 | 1 | 1 | 1 | 1 | 1 | 1 | 1 | 1 | 1 | 1 |
| Poales | *Miscanthus sinensis* | NC_028721 | 1 | 1 | 1 | 1 | 1 | 1 | 1 | 1 | 1 | 1 | 1 |
| Poales | *Monachather paradoxus* | NC_025237 | 1 | 1 | 1 | 1 | 1 | 1 | 1 | 1 | 1 | 1 | 1 |
| Poales | *Neohouzeaua* sp | NC_026963 | 1 | 1 | 1 | 1 | 1 | 1 | 1 | 1 | 1 | 1 | 1 |
| Poales | *Neololeba atra* | NC_026964 | 1 | 1 | 1 | 1 | 1 | 1 | 1 | 1 | 1 | 1 | 1 |
| Poales | *Neyraudia reynaudiana* | NC_024262 | 1 | 1 | 1 | 1 | 1 | 1 | 1 | 1 | 1 | 1 | 1 |
| Poales | *Oligostachyum shiuyingianum* | NC_024722 | 1 | 1 | 1 | 1 | 1 | 1 | 1 | 1 | 1 | 1 | 1 |
| Poales | *Olmeca reflexa* | NC_026965 | 1 | 1 | 1 | 1 | 1 | 1 | 1 | 1 | 1 | 1 | 1 |
| Poales | *Olyra latifolia* | NC_024165 | 1 | 1 | 1 | 1 | 1 | 1 | 1 | 1 | 1 | 1 | 1 |
| Poales | *Oryza australiensis* | NC_024608 | 1 | 1 | 1 | 1 | 1 | 1 | 1 | 1 | 1 | 1 | 1 |
| Poales | *Oryza barthii* | NC_027460 | 1 | 1 | 1 | 1 | 1 | 1 | 1 | 1 | 1 | 1 | 1 |
| Poales | *Oryza glaberrima* | NC_024175 | 1 | 1 | 1 | 1 | 1 | 1 | 1 | 1 | 1 | 1 | 1 |
| Poales | *Oryza glumipatula* | NC_027461 | 1 | 1 | 1 | 1 | 1 | 1 | 1 | 1 | 1 | 1 | 1 |
| Poales | *Oryza longistaminata* | NC_027462 | 1 | 1 | 1 | 1 | 1 | 1 | 1 | 1 | 1 | 1 | 1 |
| Poales | *Oryza meridionalis* | NC_016927 | 1 | 1 | 1 | 1 | 1 | 1 | 1 | 1 | 1 | 1 | 1 |
| Poales | *Oryza nivara* | NC_005973 | 1 | 1 | 1 | 1 | 1 | 1 | 1 | 1 | 1 | 1 | 1 |
| Poales | *Oryza officinalis* | NC_027463 | 1 | 1 | 1 | 1 | 1 | 1 | 1 | 1 | 1 | 1 | 1 |
| Poales | *Oryza punctata* | NC_027676 | 1 | 1 | 1 | 1 | 1 | 1 | 1 | 1 | 1 | 1 | 1 |
| Poales | *Oryza rufipogon* | NC_017835 | 1 | 1 | 1 | 1 | 1 | 1 | 1 | 1 | 1 | 1 | 1 |
| Poales | *Oryza sativa* Indica Group 1 | NC_027678 | 1 | 1 | 1 | 1 | 1 | 1 | 1 | 1 | 1 | 1 | 1 |
| Poales | *Oryza sativa* Indica Group 2 | NC_008155 | 1 | 1 | 1 | 1 | 1 | 1 | 1 | 1 | 1 | 1 | 1 |
| Poales | *Oryza sativa* Japonica Group | NC_001320 | 1 | 1 | 1 | 1 | 1 | 1 | 1 | 1 | 1 | 1 | 1 |
| Poales | *Oryzopsis asperifolia* | NC_027479 | 1 | 1 | 1 | 1 | 1 | 1 | 1 | 1 | 1 | 1 | 1 |
| Poales | *Otatea acuminata* | NC_026971 | 1 | 1 | 1 | 1 | 1 | 1 | 1 | 1 | 1 | 1 | 1 |
| Poales | *Otatea glauca* | NC_028631 | 1 | 1 | 1 | 1 | 1 | 1 | 1 | 1 | 1 | 1 | 1 |
| Poales | *Panicum virgatum* | NC_015990 | 1 | 1 | 1 | 1 | 1 | 1 | 1 | 1 | 1 | 1 | 1 |
| Poales | *Pariana campestris* | NC_027491 | 1 | 1 | 1 | 1 | 1 | 1 | 1 | 1 | 1 | 1 | 1 |
| Poales | *Pariana radiciflora* | NC_026972 | 1 | 1 | 1 | 1 | 1 | 1 | 1 | 1 | 1 | 1 | 1 |
| Poales | *Phaenosperma globosum* | NC_027480 | 1 | 1 | 1 | 1 | 1 | 1 | 1 | 1 | 1 | 1 | 1 |
| Poales | *Phalaris arundinacea* | NC_027481 | 1 | 1 | 1 | 1 | 1 | 1 | 1 | 1 | 1 | 1 | 1 |
| Poales | *Pharus lappulaceus* | NC_023245 | 1 | 1 | 1 | 1 | 1 | 1 | 1 | 1 | 1 | 1 | 1 |
| Poales | *Pharus latifolius* | NC_021372 | 1 | 1 | 1 | 1 | 1 | 1 | 1 | 1 | 1 | 1 | 1 |
| Poales | *Phleum alpinum* | NC_027482 | 1 | 1 | 1 | 1 | 1 | 1 | 1 | 1 | 1 | 1 | 1 |
| Poales | *Phragmites australis* | NC_022958 | 1 | 1 | 1 | 1 | 1 | 1 | 1 | 1 | 1 | 1 | 1 |
| Poales | *Phyllostachys edulis* | NC_015817 | 1 | 1 | 1 | 1 | 1 | 1 | 1 | 1 | 1 | 1 | 1 |
| Poales | *Phyllostachys nigra* var*. henonis* | NC_015826 | 1 | 1 | 1 | 1 | 1 | 1 | 1 | 1 | 1 | 1 | 1 |
| Poales | *Phyllostachys propinqua* | NC_016699 | 1 | 1 | 1 | 1 | 1 | 1 | 1 | 1 | 1 | 1 | 1 |
| Poales | *Phyllostachys sulphurea* | NC_024669 | 1 | 1 | 1 | 1 | 1 | 1 | 1 | 1 | 1 | 1 | 1 |
| Poales | *Piptochaetium avenaceum* | NC_027483 | 1 | 1 | 1 | 1 | 1 | 1 | 1 | 1 | 1 | 1 | 1 |
| Poales | *Pleioblastus maculatus* | NC_024723 | 1 | 1 | 1 | 1 | 1 | 1 | 1 | 1 | 1 | 1 | 1 |
| Poales | *Poa palustris* | NC_027484 | 1 | 1 | 1 | 1 | 1 | 1 | 1 | 1 | 1 | 1 | 1 |
| Poales | *Pseudosasa japonica* | NC_028328 | 1 | 1 | 1 | 1 | 1 | 1 | 1 | 1 | 1 | 1 | 1 |
| Poales | *Puccinellia nuttalliana* | NC_027485 | 1 | 1 | 1 | 1 | 1 | 1 | 1 | 1 | 1 | 1 | 1 |
| Poales | *Puelia olyriformis* | NC_023449 | 1 | 1 | 1 | 1 | 1 | 1 | 1 | 1 | 1 | 1 | 1 |
| Poales | *Raddia brasiliensis* | NC_026966 | 1 | 1 | 1 | 1 | 1 | 1 | 1 | 1 | 1 | 1 | 1 |
| Poales | *Rhynchoryza subulata* | NC_016718 | 1 | 1 | 1 | 1 | 1 | 1 | 1 | 1 | 1 | 1 | 1 |
| Poales | *Saccharum* hybrid cultivar NCo 310 | NC_006084 | 1 | 1 | 1 | 1 | 1 | 1 | 1 | 1 | 1 | 1 | 1 |
| Poales | *Saccharum* hybrid cultivar SP80-3280 | NC_005878 | 1 | 1 | 1 | 1 | 1 | 1 | 1 | 1 | 1 | 1 | 1 |
| Poales | *Sarocalamus faberi* | NC_024713 | 1 | 1 | 1 | 1 | 1 | 1 | 1 | 1 | 1 | 1 | 1 |
| Poales | *Sartidia dewinteri* | NC_027147 | 1 | 1 | 1 | 1 | 1 | 1 | 1 | 1 | 1 | 1 | 1 |
| Poales | *Sartidia perrieri* | NC_027146 | 1 | 1 | 1 | 1 | 1 | 1 | 1 | 1 | 1 | 1 | 1 |
| Poales | *Secale cereale* | NC_021761 | 1 | 1 | 1 | 1 | 1 | 1 | 1 | 1 | 1 | 1 | 1 |
| Poales | *Setaria italica* | NC_022850 | 1 | 1 | 1 | 1 | 1 | 1 | 1 | 1 | 1 | 1 | 1 |
| Poales | *Setaria viridis* | NC_028075 | 1 | 1 | 1 | 1 | 1 | 1 | 1 | 1 | 1 | 1 | 1 |
| Poales | *Sorghum bicolor* | NC_008602 | 1 | 1 | 1 | 1 | 1 | 1 | 1 | 1 | 1 | 1 | 1 |
| Poales | *Sorghum timorense* | NC_023800 | 1 | 1 | 1 | 1 | 1 | 1 | 1 | 1 | 1 | 1 | 1 |
| Poales | *Sporobolus maritimus* | NC_027650 | 1 | 1 | 1 | 1 | 1 | 1 | 1 | 1 | 1 | 1 | 1 |
| Poales | *Stipa hymenoides* | NC_027464 | 1 | 1 | 1 | 1 | 1 | 1 | 1 | 1 | 1 | 1 | 1 |
| Poales | *Stipa lipskyi* | NC_028444 | 1 | 1 | 1 | 1 | 1 | 1 | 1 | 1 | 1 | 1 | 1 |
| Poales | *Thamnocalamus spathiflorus* | NC_024724 | 1 | 1 | 1 | 1 | 1 | 1 | 1 | 1 | 1 | 1 | 1 |
| Poales | *Thysanolaena latifolia* | NC_025238 | 1 | 1 | 1 | 1 | 1 | 1 | 1 | 1 | 1 | 1 | 1 |
| Poales | *Torreyochloa pallida* | NC_027486 | 1 | 1 | 1 | 1 | 1 | 1 | 1 | 1 | 1 | 1 | 1 |
| Poales | *Trisetum cernuum* | NC_027487 | 1 | 1 | 1 | 1 | 1 | 1 | 1 | 1 | 1 | 1 | 1 |
| Poales | *Triticum aestivum* | NC_002762 | 1 | 1 | 1 | 1 | 1 | 1 | 1 | 1 | 1 | 1 | 1 |
| Poales | *Triticum macha* | NC_025955 | 1 | 1 | 1 | 1 | 1 | 1 | 1 | 1 | 1 | 1 | 1 |
| Poales | *Triticum monococcum* | NC_021760 | 1 | 1 | 1 | 1 | 1 | 1 | 1 | 1 | 1 | 1 | 1 |
| Poales | *Triticum timopheevii* | NC_024764 | 1 | 1 | 1 | 1 | 1 | 1 | 1 | 1 | 1 | 1 | 1 |
| Poales | *Triticum turgidum* | NC_024814 | 1 | 1 | 1 | 1 | 1 | 1 | 1 | 1 | 1 | 1 | 1 |
| Poales | *Triticum urartu* | NC_021762 | 1 | 1 | 1 | 1 | 1 | 1 | 1 | 1 | 1 | 1 | 1 |
| Poales | *Typha latifolia* | NC_013823 | 1 | 1 | 1 | 1 | 1 | 1 | 1 | 1 | 1 | 1 | 1 |
| Poales | *Yushania levigata* | NC_024725 | 1 | 1 | 1 | 1 | 1 | 1 | 1 | 1 | 1 | 1 | 1 |
| Poales | *Zea mays* | NC_001666 | 1 | 1 | 1 | 1 | 1 | 1 | 1 | 1 | 1 | 1 | 1 |
| Poales | *Zizania aquatica* | NC_026967 | 1 | 1 | 1 | 1 | 1 | 1 | 1 | 1 | 1 | 1 | 1 |
| Zingiberales | *Curcuma flaviflora* | NC_028729 | 1 | 1 | 1 | 1 | 1 | 1 | 1 | 1 | 1 | 1 | 1 |
| Zingiberales | *Curcuma roscoeana* | NC_022928 | 1 | 1 | 1 | 1 | 1 | 1 | 1 | 1 | 1 | 1 | 1 |
| Zingiberales | *Heliconia collinsiana* | NC_020362 | 1 | 1 | 1 | 1 | 1 | 1 | 1 | 1 | 1 | 1 | 1 |
| Zingiberales | *Musa balbisiana* | NC_028439 | 1 | 1 | 1 | 1 | 1 | 1 | 1 | 1 | 1 | 1 | 1 |
| Zingiberales | *Musa textilis* | NC_022926 | 1 | 1 | 1 | 1 | 1 | 1 | 1 | 1 | 1 | 1 | 1 |
| Zingiberales | *Ravenala madagascariensis* | NC_022927 | 1 | 1 | 1 | 1 | 1 | 1 | 1 | 1 | 1 | 1 | 1 |
| Zingiberales | *Zingiber spectabile* | NC_020363 | 1 | 1 | 1 | 1 | 1 | 1 | 1 | 1 | 1 | 1 | 1 |
| Ranunculales | *Berberis bealei* | NC_022457 | 1 | 1 | 1 | 1 | 1 | 1 | 1 | 1 | 1 | 1 | 1 |
| Ranunculales | *Clematis terniflora* | NC_028000 | 1 | 1 | 1 | 1 | 1 | 1 | 1 | 1 | 1 | 1 | 1 |
| Ranunculales | *Megaleranthis saniculifolia* | NC_012615 | 1 | 1 | 1 | 1 | 1 | 1 | 1 | 1 | 1 | 1 | 1 |
| Ranunculales | *Nandina domestica* | NC_008336 | 1 | 1 | 1 | 1 | 1 | 1 | 1 | 1 | 1 | 1 | 1 |
| Ranunculales | *Ranunculus macranthus* | NC_008796 | 1 | 1 | 1 | 1 | 1 | 1 | 1 | 1 | 1 | 1 | 1 |
| Ranunculales | *Sinopodophyllum hexandrum* | NC_027732 | 1 | 1 | 1 | 1 | 1 | 1 | 1 | 1 | 1 | 1 | 1 |
| Ranunculales | *Thalictrum coreanum* | NC_026103 | 1 | 1 | 1 | 1 | 1 | 1 | 1 | 1 | 1 | 1 | 1 |
| Proteales | *Macadamia integrifolia* | NC_025288 | 1 | 1 | 1 | 1 | 1 | 1 | 1 | 1 | 1 | 1 | 1 |
| Proteales | *Nelumbo lutea* | NC_015605 | 1 | 1 | 1 | 1 | 1 | 1 | 1 | 1 | 1 | 1 | 1 |
| Proteales | *Nelumbo nucifera* | NC_025339 | 1 | 1 | 1 | 1 | 1 | 1 | 1 | 1 | 1 | 1 | 1 |
| Proteales | *Platanus occidentalis* | NC_008335 | 1 | 1 | 1 | 1 | 1 | 1 | 1 | 1 | 1 | 1 | 1 |
| Trochodendrales | *Tetracentron sinense* | NC_021425 | 1 | 1 | 1 | 1 | 1 | 1 | 1 | 1 | 1 | 1 | 1 |
| Trochodendrales | *Trochodendron aralioides* | NC_021426 | 1 | 1 | 1 | 1 | 1 | 1 | 1 | 1 | 1 | 1 | 1 |
| Buxales | *Buxus microphylla* | NC_009599 | 1 | 1 | 1 | 1 | 1 | 1 | 1 | 1 | 1 | 1 | 1 |
| Saxifragales | *Liquidambar formosana* | NC_023092 | 1 | 1 | 1 | 1 | 1 | 1 | 1 | 1 | 1 | 1 | 1 |
| Saxifragales | *Paeonia obovata* | NC_026076 | 1 | 1 | 1 | 1 | 1 | 1 | 1 | 1 | 1 | 1 | 1 |
| Saxifragales | *Penthorum chinense* | NC_023086 | 1 | 1 | 1 | 1 | 1 | 1 | 1 | 1 | 1 | 1 | 1 |
| Saxifragales | *Sedum oryzifolium* | NC_027837 | 1 | 1 | 1 | 1 | 1 | 1 | 1 | 1 | 1 | 1 | 1 |
| Saxifragales | *Sedum sarmentosum* | NC_023085 | 1 | 1 | 1 | 1 | 1 | 1 | 1 | 1 | 1 | 1 | 1 |
| Saxifragales | *Sedum takesimense* | NC_026065 | 1 | 1 | 1 | 1 | 1 | 1 | 1 | 1 | 1 | 1 | 1 |
| Vitales | *Vitis rotundifolia* | NC_023790 | 1 | 1 | 1 | 1 | 1 | 1 | 1 | 1 | 1 | 1 | 1 |

Supplementary table S1. Continues

| Order | Species | Accession | *ndhA* | *ndhB* | *ndhC* | *ndhD* | *ndhE* | *ndhF* | *ndhG* | *ndhH* | *ndhI* | *ndhJ* | *ndhK* |
| --- | --- | --- | --- | --- | --- | --- | --- | --- | --- | --- | --- | --- | --- |
| Vitales | *Vitis vinifera* | NC_007957 | 1 | 1 | 1 | 1 | 1 | 1 | 1 | 1 | 1 | 1 | 1 |
| Zygophyllales | *Larrea tridentata* | NC_028023 | 1 | 1 | 1 | 1 | 1 | 1 | 1 | 1 | 1 | 1 | 1 |
| Celastrales | *Euonymus japonicus* | NC_028067 | 1 | 1 | 1 | 1 | 1 | 1 | 1 | 1 | 1 | 1 | 1 |
| Malpighiales | *Chrysobalanus icaco* | NC_024061 | 1 | 1 | 1 | 1 | 1 | 1 | 1 | 1 | 1 | 1 | 1 |
| Malpighiales | *Couepia guianensis* | NC_024063 | 1 | 1 | 1 | 1 | 1 | 1 | 1 | 1 | 1 | 1 | 1 |
| Malpighiales | *Hevea brasiliensis* | NC_015308 | 1 | 1 | 1 | 1 | 1 | 1 | 1 | 1 | 1 | 1 | 1 |
| Malpighiales | *Hirtella physophora* | NC_024066 | 1 | 1 | 1 | 1 | 1 | 1 | 1 | 1 | 1 | 1 | 1 |
| Malpighiales | *Hirtella racemosa* | NC_024060 | 1 | 1 | 1 | 1 | 1 | 1 | 1 | 1 | 1 | 1 | 1 |
| Malpighiales | *Jatropha curcas* | NC_012224 | 1 | 1 | 1 | 1 | 1 | 1 | 1 | 1 | 1 | 1 | 1 |
| Malpighiales | *Licania alba* | NC_024064 | 1 | 1 | 1 | 1 | 1 | 1 | 1 | 1 | 1 | 1 | 1 |
| Malpighiales | *Licania heteromorpha* | NC_024062 | 1 | 1 | 1 | 1 | 1 | 1 | 1 | 1 | 1 | 1 | 1 |
| Malpighiales | *Licania sprucei* | NC_024065 | 1 | 1 | 1 | 1 | 1 | 1 | 1 | 1 | 1 | 1 | 1 |
| Malpighiales | *Manihot esculenta* | NC_010433 | 1 | 1 | 1 | 1 | 1 | 1 | 1 | 1 | 1 | 1 | 1 |
| Malpighiales | *Parinari campestris* | NC_024067 | 1 | 1 | 1 | 1 | 1 | 1 | 1 | 1 | 1 | 1 | 1 |
| Malpighiales | *Populus alba* | NC_008235 | 1 | 1 | 1 | 1 | 1 | 1 | 1 | 1 | 1 | 1 | 1 |
| Malpighiales | *Populus balsamifera* | NC_024735 | 1 | 1 | 1 | 1 | 1 | 1 | 1 | 1 | 1 | 1 | 1 |
| Malpighiales | *Populus euphratica* | NC_024747 | 1 | 1 | 1 | 1 | 1 | 1 | 1 | 1 | 1 | 1 | 1 |
| Malpighiales | *Populus fremontii* | NC_024734 | 1 | 1 | 1 | 1 | 1 | 1 | 1 | 1 | 1 | 1 | 1 |
| Malpighiales | *Populus tremula* | NC_027425 | 1 | 1 | 1 | 1 | 1 | 1 | 1 | 1 | 1 | 1 | 1 |
| Malpighiales | *Populus tremula x Populus alba* | NC_028504 | 1 | 1 | 1 | 1 | 1 | 1 | 1 | 1 | 1 | 1 | 1 |
| Malpighiales | *Populus trichocarpa* | NC_009143 | 1 | 1 | 1 | 1 | 1 | 1 | 1 | 1 | 1 | 1 | 1 |
| Malpighiales | *Ricinus communis* | NC_016736 | 1 | 1 | 1 | 1 | 1 | 1 | 1 | 1 | 1 | 1 | 1 |
| Malpighiales | *Salix babylonica* | NC_028350 | 1 | 1 | 1 | 1 | 1 | 1 | 1 | 1 | 1 | 1 | 1 |
| Malpighiales | *Salix interior* | NC_024681 | 1 | 1 | 1 | 1 | 1 | 1 | 1 | 1 | 1 | 1 | 1 |
| Malpighiales | *Salix purpurea* | NC_026722 | 1 | 1 | 1 | 1 | 1 | 1 | 1 | 1 | 1 | 1 | 1 |
| Malpighiales | *Salix suchowensis* | NC_026462 | 1 | 1 | 1 | 1 | 1 | 1 | 1 | 1 | 1 | 1 | 1 |
| Malpighiales | *Viola seoulensis* | NC_026986 | 1 | 1 | 1 | 1 | 1 | 1 | 1 | 1 | 1 | 1 | 1 |
| Fabales | *Acacia ligulata* | NC_026134 | 1 | 1 | 1 | 1 | 1 | 1 | 1 | 1 | 1 | 1 | 1 |
| Fabales | *Astragalus nakaianus* | NC_028171 | 1 | 1 | 1 | 1 | 1 | 1 | 1 | 1 | 1 | 1 | 1 |
| Fabales | *Cicer arietinum* | NC_011163 | 1 | 1 | 1 | 1 | 1 | 1 | 1 | 1 | 1 | 1 | 1 |
| Fabales | *Glycine canescens* | NC_021647 | 1 | 1 | 1 | 1 | 1 | 1 | 1 | 1 | 1 | 1 | 1 |
| Fabales | *Glycine cyrtoloba* | NC_021645 | 1 | 1 | 1 | 1 | 1 | 1 | 1 | 1 | 1 | 1 | 1 |
| Fabales | *Glycine dolichocarpa* | NC_021648 | 1 | 1 | 1 | 1 | 1 | 1 | 1 | 1 | 1 | 1 | 1 |
| Fabales | *Glycine falcata* | NC_021649 | 1 | 1 | 1 | 1 | 1 | 1 | 1 | 1 | 1 | 1 | 1 |
| Fabales | *Glycine max* | NC_007942 | 1 | 1 | 1 | 1 | 1 | 1 | 1 | 1 | 1 | 1 | 1 |
| Fabales | *Glycine soja* | NC_022868 | 1 | 1 | 1 | 1 | 1 | 1 | 1 | 1 | 1 | 1 | 1 |
| Fabales | *Glycine stenophita* | NC_021646 | 1 | 1 | 1 | 1 | 1 | 1 | 1 | 1 | 1 | 1 | 1 |
| Fabales | *Glycine syndetika* | NC_021650 | 1 | 1 | 1 | 1 | 1 | 1 | 1 | 1 | 1 | 1 | 1 |
| Fabales | *Glycine tomentella* | NC_021636 | 1 | 1 | 1 | 1 | 1 | 1 | 1 | 1 | 1 | 1 | 1 |
| Fabales | *Glycyrrhiza glabra* | NC_024038 | 1 | 1 | 1 | 1 | 1 | 1 | 1 | 1 | 1 | 1 | 1 |
| Fabales | *Inga leiocalycina* | NC_028732 | 1 | 1 | 1 | 1 | 1 | 1 | 1 | 1 | 1 | 1 | 1 |
| Fabales | *Lathyrus clymenum* | NC_027148 | 1 | 1 | 1 | 1 | 1 | 1 | 1 | 1 | 1 | 1 | 1 |
| Fabales | *Lathyrus davidii* | NC_027073 | 1 | 1 | 1 | 1 | 1 | 1 | 1 | 1 | 1 | 1 | 1 |
| Fabales | *Lathyrus graminifolius* | NC_027074 | 1 | 1 | 1 | 1 | 1 | 1 | 1 | 1 | 1 | 1 | 1 |
| Fabales | *Lathyrus inconspicuus* | NC_027149 | 1 | 1 | 1 | 1 | 1 | 1 | 1 | 1 | 1 | 1 | 1 |
| Fabales | *Lathyrus japonicus* | NC_027075 | 1 | 1 | 1 | 1 | 1 | 1 | 1 | 1 | 1 | 1 | 1 |
| Fabales | *Lathyrus littoralis* | NC_027076 | 1 | 1 | 1 | 1 | 1 | 1 | 1 | 1 | 1 | 1 | 1 |
| Fabales | *Lathyrus ochroleucus* | NC_027077 | 1 | 1 | 1 | 1 | 1 | 1 | 1 | 1 | 1 | 1 | 1 |
| Fabales | *Lathyrus odoratus* | NC_027150 | 1 | 1 | 1 | 1 | 1 | 1 | 1 | 1 | 1 | 1 | 1 |
| Fabales | *Lathyrus palustris* | NC_027078 | 1 | 1 | 1 | 1 | 1 | 1 | 1 | 1 | 1 | 1 | 1 |
| Fabales | *Lathyrus pubescens* | NC_027079 | 1 | 1 | 1 | 1 | 1 | 1 | 1 | 1 | 1 | 1 | 1 |
| Fabales | *Lathyrus sativus* | NC_014063 | 1 | 1 | 1 | 1 | 1 | 1 | 1 | 1 | 1 | 1 | 1 |
| Fabales | *Lathyrus tingitanus* | NC_027151 | 1 | 1 | 1 | 1 | 1 | 1 | 1 | 1 | 1 | 1 | 1 |
| Fabales | *Lathyrus venosus* | NC_027080 | 1 | 1 | 1 | 1 | 1 | 1 | 1 | 1 | 1 | 1 | 1 |
| Fabales | *Lens culinaris* | NC_027152 | 1 | 1 | 1 | 1 | 1 | 1 | 1 | 1 | 1 | 1 | 1 |
| Fabales | *Leucaena trichandra* | NC_028733 | 1 | 1 | 1 | 1 | 1 | 1 | 1 | 1 | 1 | 1 | 1 |
| Fabales | *Lotus japonicus* | NC_002694 | 1 | 1 | 1 | 1 | 1 | 1 | 1 | 1 | 1 | 1 | 1 |
| Fabales | *Lupinus luteus* | NC_023090 | 1 | 1 | 1 | 1 | 1 | 1 | 1 | 1 | 1 | 1 | 1 |
| Fabales | *Medicago hybrida* | NC_027153 | 1 | 1 | 1 | 1 | 1 | 1 | 1 | 1 | 1 | 1 | 1 |
| Fabales | *Medicago papillosa* | NC_027154 | 1 | 1 | 1 | 2 | 1 | 1 | 1 | 1 | 1 | 1 | 1 |
| Fabales | *Medicago truncatula* | NC_003119 | 1 | 1 | 1 | 1 | 1 | 1 | 1 | 1 | 1 | 1 | 1 |
| Fabales | *Millettia pinnata* | NC_016708 | 1 | 1 | 1 | 1 | 1 | 1 | 1 | 1 | 1 | 1 | 1 |
| Fabales | *Phaseolus vulgaris* | NC_009259 | 1 | 1 | 1 | 1 | 1 | 1 | 1 | 1 | 1 | 1 | 1 |
| Fabales | *Pisum sativum* | NC_014057 | 1 | 1 | 1 | 1 | 1 | 1 | 1 | 1 | 1 | 1 | 1 |
| Fabales | *Trifolium aureum* | NC_024035 | 1 | 1 | 1 | 1 | 1 | 1 | 1 | 1 | 1 | 1 | 1 |
| Fabales | *Trifolium boissieri* | NC_025743 | 1 | 1 | 1 | 1 | 1 | 1 | 1 | 1 | 1 | 1 | 1 |
| Fabales | *Trifolium glanduliferum* | NC_025744 | 1 | 1 | 1 | 1 | 1 | 1 | 1 | 1 | 1 | 1 | 1 |
| Fabales | *Trifolium grandiflorum* | NC_024034 | 1 | 1 | 1 | 1 | 1 | 1 | 1 | 1 | 1 | 1 | 1 |
| Fabales | *Trifolium meduseum* | NC_024166 | 1 | 1 | 1 | 1 | 1 | 1 | 1 | 1 | 1 | 1 | 1 |
| Fabales | *Trifolium repens* | NC_024036 | 1 | 1 | 1 | 1 | 1 | 1 | 1 | 1 | 1 | 1 | 1 |
| Fabales | *Trifolium strictum* | NC_025745 | 1 | 1 | 1 | 1 | 1 | 1 | 1 | 1 | 1 | 1 | 1 |
| Fabales | *Trifolium subterraneum* | NC_011828 | 1 | 1 | 1 | 1 | 1 | 1 | 1 | 1 | 1 | 1 | 1 |
| Fabales | *Vicia sativa* | NC_027155 | 1 | 1 | 1 | 1 | 1 | 1 | 1 | 1 | 1 | 1 | 1 |
| Fabales | *Vigna angularis* | NC_021091 | 1 | 1 | 1 | 1 | 1 | 1 | 1 | 1 | 1 | 1 | 1 |
| Fabales | *Vigna radiata* | NC_013843 | 1 | 1 | 1 | 1 | 1 | 1 | 1 | 1 | 1 | 1 | 1 |
| Fabales | *Vigna unguiculata* | NC_018051 | 1 | 1 | 1 | 1 | 1 | 1 | 1 | 1 | 1 | 1 | 1 |
| Fabales | *Wisteria floribunda* | NC_027677 | 1 | 1 | 1 | 1 | 1 | 1 | 1 | 1 | 1 | 1 | 1 |
| Rosales | *Cannabis sativa* 1 | NC_027223 | 1 | 1 | 1 | 1 | 1 | 1 | 1 | 1 | 1 | 1 | 1 |
| Rosales | *Cannabis sativa* 2 | NC_026562 | 1 | 1 | 1 | 1 | 1 | 1 | 1 | 1 | 1 | 1 | 1 |
| Rosales | *Elaeagnus macrophylla* | NC_028066 | 1 | 1 | 1 | 1 | 1 | 1 | 1 | 1 | 1 | 1 | 1 |
| Rosales | *Ficus racemosa* | NC_028185 | 1 | 1 | 1 | 1 | 1 | 1 | 1 | 1 | 1 | 1 | 1 |
| Rosales | *Fragaria chiloensis* | NC_019601 | 1 | 1 | 1 | 1 | 1 | 1 | 1 | 1 | 1 | 1 | 1 |
| Rosales | *Fragaria iinumae* | NC_024258 | 1 | 1 | 1 | 1 | 1 | 1 | 1 | 1 | 1 | 1 | 1 |
| Rosales | *Fragaria mandshurica* | NC_018767 | 1 | 1 | 1 | 1 | 1 | 1 | 1 | 1 | 1 | 1 | 1 |
| Rosales | *Fragaria vesca* subsp*. bracteata* | NC_018766 | 1 | 1 | 1 | 1 | 1 | 1 | 1 | 1 | 1 | 1 | 1 |
| Rosales | *Fragaria vesca* subsp*. vesca* | NC_015206 | 1 | 1 | 1 | 1 | 1 | 1 | 1 | 1 | 1 | 1 | 1 |
| Rosales | *Fragaria virginiana* | NC_019602 | 1 | 1 | 1 | 1 | 1 | 1 | 1 | 1 | 1 | 1 | 1 |
| Rosales | *Humulus lupulus* | NC_028032 | 1 | 1 | 1 | 1 | 1 | 1 | 1 | 1 | 1 | 1 | 1 |
| Rosales | *Morus indica* | NC_008359 | 1 | 1 | 1 | 1 | 1 | 1 | 1 | 1 | 1 | 1 | 1 |
| Rosales | *Morus mongolica* | NC_025772 | 1 | 1 | 1 | 1 | 1 | 1 | 1 | 1 | 1 | 1 | 1 |
| Rosales | *Morus notabilis* | NC_027110 | 1 | 1 | 1 | 1 | 1 | 1 | 1 | 1 | 1 | 1 | 1 |
| Rosales | *Pentactina rupicola* | NC_016921 | 1 | 1 | 1 | 1 | 1 | 1 | 1 | 1 | 1 | 1 | 1 |
| Rosales | *Prinsepia utilis* | NC_021455 | 1 | 1 | 1 | 1 | 1 | 1 | 1 | 1 | 1 | 1 | 1 |
| Rosales | *Prunus kansuensis* | NC_023956 | 1 | 1 | 1 | 1 | 1 | 1 | 1 | 1 | 1 | 1 | 1 |
| Rosales | *Prunus maximowiczii* | NC_026981 | 1 | 1 | 1 | 1 | 1 | 1 | 1 | 1 | 1 | 1 | 1 |
| Rosales | *Prunus mume* | NC_023798 | 1 | 1 | 1 | 1 | 1 | 1 | 1 | 1 | 1 | 1 | 1 |
| Rosales | *Prunus padus* | NC_026982 | 1 | 1 | 1 | 1 | 1 | 1 | 1 | 1 | 1 | 1 | 1 |
| Rosales | *Prunus persica* | NC_014697 | 1 | 1 | 1 | 1 | 1 | 1 | 1 | 1 | 1 | 1 | 1 |
| Rosales | *Prunus yedoensis* | NC_026980 | 1 | 1 | 1 | 1 | 1 | 1 | 1 | 1 | 1 | 1 | 1 |
| Rosales | *Pyrus pyrifolia* | NC_015996 | 1 | 1 | 1 | 1 | 1 | 1 | 1 | 1 | 1 | 1 | 1 |
| Rosales | *Pyrus spinosa* | NC_023130 | 1 | 1 | 1 | 1 | 1 | 1 | 1 | 1 | 1 | 1 | 1 |
| Cucurbitales | *Corynocarpus laevigata* | NC_014807 | 1 | 1 | 1 | 1 | 1 | 1 | 1 | 1 | 1 | 1 | 1 |
| Cucurbitales | *Cucumis hystrix* | NC_023544 | 1 | 1 | 1 | 1 | 1 | 1 | 1 | 1 | 1 | 1 | 1 |
| Cucurbitales | *Cucumis melo* subsp*. melo* | NC_015983 | 1 | 1 | 1 | 1 | 1 | 1 | 1 | 1 | 1 | 1 | 1 |
| Cucurbitales | *Cucumis sativus* | NC_007144 | 1 | 1 | 1 | 1 | 1 | 1 | 1 | 1 | 1 | 1 | 1 |
| Fagales | *Castanea mollissima* | NC_014674 | 1 | 1 | 1 | 1 | 1 | 1 | 1 | 1 | 1 | 1 | 1 |
| Fagales | *Castanopsis echinocarpa* | NC_023801 | 1 | 1 | 1 | 1 | 1 | 1 | 1 | 1 | 1 | 1 | 1 |

Supplementary table S1. Continues

| Order | Species | Accession | *ndhA* | *ndhB* | *ndhC* | *ndhD* | *ndhE* | *ndhF* | *ndhG* | *ndhH* | *ndhI* | *ndhJ* | *ndhK* |
| --- | --- | --- | --- | --- | --- | --- | --- | --- | --- | --- | --- | --- | --- |
| Fagales | *Juglans regia* | NC_028617 | 1 | 1 | 1 | 1 | 1 | 1 | 1 | 1 | 1 | 1 | 1 |
| Fagales | *Lithocarpus balansae* | NC_026577 | 1 | 1 | 1 | 1 | 1 | 1 | 1 | 1 | 1 | 1 | 1 |
| Fagales | *Ostrya rehderiana* | NC_028349 | 1 | 1 | 1 | 1 | 1 | 1 | 1 | 1 | 1 | 1 | 1 |
| Fagales | *Quercus aliena* | NC_026790 | 1 | 1 | 1 | 1 | 1 | 1 | 1 | 1 | 1 | 1 | 1 |
| Fagales | *Quercus aquifolioides* | NC_026913 | 1 | 1 | 1 | 1 | 1 | 1 | 1 | 1 | 1 | 1 | 1 |
| Fagales | *Quercus rubra* | NC_020152 | 1 | 1 | 1 | 1 | 1 | 1 | 1 | 1 | 1 | 1 | 1 |
| Fagales | *Quercus spinosa* | NC_026907 | 1 | 1 | 1 | 1 | 1 | 1 | 1 | 1 | 1 | 1 | 1 |
| Fagales | *Trigonobalanus doichangensis* | NC_023959 | 1 | 1 | 1 | 1 | 1 | 1 | 1 | 1 | 1 | 1 | 1 |
| Geraniales | *Erodium absinthoides* | NC_026847 | 3 | 2 | 4 | 2 | 4 | 3 | 4 | 4 | 4 | 2 | 4 |
| Geraniales | *Erodium carvifolium* | NC_015083 | 1 | 1 | 1 | 1 | 1 | 1 | 1 | 1 | 1 | 1 | 1 |
| Geraniales | *Erodium chrysanthum* | NC_027065 | 3 | 2 | 4 | 2 | 4 | 3 | 4 | 4 | 4 | 2 | 4 |
| Geraniales | *Erodium crassifolium* | NC_025906 | 1 | 1 | 1 | 1 | 1 | 1 | 1 | 1 | 1 | 1 | 1 |
| Geraniales | *Erodium gruinum* | NC_025907 | 3 | 2 | 2 | 2 | 4 | 2 | 4 | 4 | 4 | 2 | 2 |
| Geraniales | *Erodium texanum* | NC_014569 | 1 | 1 | 1 | 1 | 1 | 1 | 1 | 1 | 1 | 1 | 1 |
| Geraniales | *Erodium trifolium* | NC_024635 | 1 | 1 | 1 | 1 | 1 | 1 | 1 | 1 | 1 | 1 | 1 |
| Geraniales | *Francoa sonchifolia* | NC_021101 | 1 | 1 | 1 | 1 | 1 | 1 | 2 | 1 | 1 | 1 | 1 |
| Geraniales | *Geranium palmatum* | NC_014573 | 1 | 1 | 1 | 1 | 1 | 1 | 1 | 1 | 1 | 1 | 1 |
| Geraniales | *Hypseocharis bilobata* | NC_023260 | 1 | 1 | 1 | 1 | 1 | 1 | 1 | 1 | 1 | 1 | 1 |
| Geraniales | *Melianthus villosus* | NC_023256 | 1 | 1 | 1 | 2 | 1 | 2 | 1 | 2 | 1 | 1 | 2 |
| Geraniales | *Monsonia speciosa* | NC_014582 | 1 | 1 | 1 | 1 | 1 | 1 | 1 | 1 | 1 | 1 | 1 |
| Geraniales | *Pelargonium alternans* | NC_023261 | 1 | 1 | 1 | 1 | 1 | 1 | 1 | 1 | 1 | 1 | 1 |
| Geraniales | *Pelargonium australe* | NC_028053 | 1 | 1 | 1 | 1 | 1 | 1 | 1 | 1 | 1 | 1 | 1 |
| Geraniales | *Pelargonium cotyledonis* | NC_028052 | 1 | 1 | 1 | 1 | 1 | 1 | 1 | 1 | 1 | 1 | 1 |
| Geraniales | *Pelargonium dichondrifolium* | NC_028051 | 1 | 1 | 1 | 1 | 1 | 1 | 1 | 1 | 1 | 1 | 1 |
| Geraniales | *Pelargonium x hortorum* | NC_008454 | 1 | 1 | 1 | 1 | 1 | 1 | 1 | 1 | 1 | 1 | 1 |
| Geraniales | *Viviania marifolia* | NC_023259 | 1 | 1 | 1 | 1 | 1 | 1 | 1 | 1 | 1 | 1 | 1 |
| Myrtales | *Allosyncarpia ternata* | NC_022413 | 1 | 1 | 1 | 1 | 1 | 1 | 1 | 1 | 1 | 1 | 1 |
| Myrtales | *Angophora costata* | NC_022412 | 1 | 1 | 1 | 1 | 1 | 1 | 1 | 1 | 1 | 1 | 1 |
| Myrtales | *Angophora floribunda* | NC_022411 | 1 | 1 | 1 | 1 | 1 | 1 | 1 | 1 | 1 | 1 | 1 |
| Myrtales | *Corymbia eximia* | NC_022409 | 1 | 1 | 1 | 1 | 1 | 1 | 1 | 1 | 1 | 1 | 1 |
| Myrtales | *Corymbia gummifera* | NC_022407 | 1 | 1 | 1 | 1 | 1 | 1 | 1 | 1 | 1 | 1 | 1 |
| Myrtales | *Corymbia henryi* | NC_028409 | 1 | 1 | 1 | 1 | 1 | 1 | 1 | 1 | 1 | 1 | 1 |
| Myrtales | *Corymbia maculata* | NC_022408 | 1 | 1 | 1 | 1 | 1 | 1 | 1 | 1 | 1 | 1 | 1 |
| Myrtales | *Corymbia tessellaris* | NC_022410 | 1 | 1 | 1 | 1 | 1 | 1 | 1 | 1 | 1 | 1 | 1 |
| Myrtales | *Corymbia torelliana* | NC_028410 | 1 | 1 | 1 | 1 | 1 | 1 | 1 | 1 | 1 | 1 | 1 |
| Myrtales | *Eucalyptus aromaphloia* | NC_022396 | 1 | 1 | 1 | 1 | 1 | 1 | 1 | 1 | 1 | 1 | 1 |
| Myrtales | *Eucalyptus baxteri* | NC_022382 | 1 | 1 | 1 | 1 | 1 | 1 | 1 | 1 | 1 | 1 | 1 |
| Myrtales | *Eucalyptus camaldulensis* | NC_022398 | 1 | 1 | 1 | 1 | 1 | 1 | 1 | 1 | 1 | 1 | 1 |
| Myrtales | *Eucalyptus cladocalyx* | NC_022394 | 1 | 1 | 1 | 1 | 1 | 1 | 1 | 1 | 1 | 1 | 1 |
| Myrtales | *Eucalyptus cloeziana* | NC_022388 | 1 | 1 | 1 | 1 | 1 | 1 | 1 | 1 | 1 | 1 | 1 |
| Myrtales | *Eucalyptus curtisii* | NC_022391 | 1 | 1 | 1 | 1 | 1 | 1 | 1 | 1 | 1 | 1 | 1 |
| Myrtales | *Eucalyptus deglupta* | NC_022399 | 1 | 1 | 1 | 1 | 1 | 1 | 1 | 1 | 1 | 1 | 1 |
| Myrtales | *Eucalyptus delegatensis* | NC_022380 | 1 | 1 | 1 | 1 | 1 | 1 | 1 | 1 | 1 | 1 | 1 |
| Myrtales | *Eucalyptus diversicolor* | NC_022402 | 1 | 1 | 1 | 1 | 1 | 1 | 1 | 1 | 1 | 1 | 1 |
| Myrtales | *Eucalyptus diversifolia* | NC_022383 | 1 | 1 | 1 | 1 | 1 | 1 | 1 | 1 | 1 | 1 | 1 |
| Myrtales | *Eucalyptus elata* | NC_022385 | 1 | 1 | 1 | 1 | 1 | 1 | 1 | 1 | 1 | 1 | 1 |
| Myrtales | *Eucalyptus erythrocorys* | NC_022406 | 1 | 1 | 1 | 1 | 1 | 1 | 1 | 1 | 1 | 1 | 1 |
| Myrtales | *Eucalyptus globulus* subsp*. globulus* | NC_008115 | 1 | 1 | 1 | 1 | 1 | 1 | 1 | 1 | 1 | 1 | 1 |
| Myrtales | *Eucalyptus grandis* | NC_014570 | 1 | 1 | 1 | 1 | 1 | 1 | 1 | 1 | 1 | 1 | 1 |
| Myrtales | *Eucalyptus guilfoylei* | NC_022405 | 1 | 1 | 1 | 1 | 1 | 1 | 1 | 1 | 1 | 1 | 1 |
| Myrtales | *Eucalyptus marginata* | NC_022390 | 1 | 1 | 1 | 1 | 1 | 1 | 1 | 1 | 1 | 1 | 1 |
| Myrtales | *Eucalyptus melliodora* | NC_022392 | 1 | 1 | 1 | 1 | 1 | 1 | 1 | 1 | 1 | 1 | 1 |
| Myrtales | *Eucalyptus microcorys* | NC_022404 | 1 | 1 | 1 | 1 | 1 | 1 | 1 | 1 | 1 | 1 | 1 |
| Myrtales | *Eucalyptus nitens* | NC_022395 | 1 | 1 | 1 | 1 | 1 | 1 | 1 | 1 | 1 | 1 | 1 |
| Myrtales | *Eucalyptus obliqua* | NC_022378 | 1 | 1 | 1 | 1 | 1 | 1 | 1 | 1 | 1 | 1 | 1 |
| Myrtales | *Eucalyptus patens* | NC_022389 | 1 | 1 | 1 | 1 | 1 | 1 | 1 | 1 | 1 | 1 | 1 |
| Myrtales | *Eucalyptus polybractea* | NC_022393 | 1 | 1 | 1 | 1 | 1 | 1 | 1 | 1 | 1 | 1 | 1 |
| Myrtales | *Eucalyptus radiata* | NC_022379 | 1 | 1 | 1 | 1 | 1 | 1 | 1 | 1 | 1 | 1 | 1 |
| Myrtales | *Eucalyptus regnans* | NC_022386 | 1 | 1 | 1 | 1 | 1 | 1 | 1 | 1 | 1 | 1 | 1 |
| Myrtales | *Eucalyptus saligna* | NC_022397 | 1 | 1 | 1 | 1 | 1 | 1 | 1 | 1 | 1 | 1 | 1 |
| Myrtales | *Eucalyptus salmonophloia* | NC_022403 | 1 | 1 | 1 | 1 | 1 | 1 | 1 | 1 | 1 | 1 | 1 |
| Myrtales | *Eucalyptus sieberi* | NC_022384 | 1 | 1 | 1 | 1 | 1 | 1 | 1 | 1 | 1 | 1 | 1 |
| Myrtales | *Eucalyptus spathulata* | NC_022400 | 1 | 1 | 1 | 1 | 1 | 1 | 1 | 1 | 1 | 1 | 1 |
| Myrtales | *Eucalyptus torquata* | NC_022401 | 1 | 1 | 1 | 1 | 1 | 1 | 1 | 1 | 1 | 1 | 1 |
| Myrtales | *Eucalyptus umbra* | NC_022387 | 1 | 1 | 1 | 1 | 1 | 1 | 1 | 1 | 1 | 1 | 1 |
| Myrtales | *Eucalyptus verrucata* | NC_022381 | 1 | 1 | 1 | 1 | 1 | 1 | 1 | 1 | 1 | 1 | 1 |
| Myrtales | *Eugenia uniflora* | NC_027744 | 1 | 1 | 1 | 1 | 1 | 1 | 1 | 1 | 1 | 1 | 1 |
| Myrtales | *Oenothera argillicola* | NC_010358 | 1 | 1 | 1 | 1 | 1 | 1 | 1 | 1 | 1 | 1 | 1 |
| Myrtales | *Oenothera biennis* | NC_010361 | 1 | 1 | 1 | 1 | 1 | 1 | 1 | 1 | 1 | 1 | 1 |
| Myrtales | *Oenothera elata* subsp*. hookeri* | NC_002693 | 1 | 1 | 1 | 1 | 1 | 1 | 1 | 1 | 1 | 1 | 1 |
| Myrtales | *Oenothera glazioviana* | NC_010360 | 1 | 1 | 1 | 1 | 1 | 1 | 1 | 1 | 1 | 1 | 1 |
| Myrtales | *Oenothera parviflora* | NC_010362 | 1 | 1 | 1 | 1 | 1 | 1 | 1 | 1 | 1 | 1 | 1 |
| Myrtales | *Stockwellia quadrifida* | NC_022414 | 1 | 1 | 1 | 1 | 1 | 1 | 1 | 1 | 1 | 1 | 1 |
| Sapindales | *Azadirachta indica* | NC_023792 | 1 | 1 | 1 | 1 | 1 | 1 | 1 | 1 | 1 | 1 | 1 |
| Sapindales | *Citrus aurantiifolia* | NC_024929 | 1 | 1 | 1 | 1 | 1 | 1 | 1 | 1 | 1 | 1 | 1 |
| Sapindales | *Citrus sinensis* | NC_008334 | 1 | 1 | 1 | 1 | 1 | 1 | 1 | 1 | 1 | 1 | 1 |
| Sapindales | *Sapindus mukorossi* | NC_025554 | 1 | 1 | 1 | 1 | 1 | 1 | 1 | 1 | 1 | 1 | 1 |
| Sapindales | *Zanthoxylum piperitum* | NC_027939 | 1 | 1 | 1 | 1 | 1 | 1 | 1 | 1 | 1 | 1 | 1 |
| Malvales | *Gossypium anomalum* | NC_023213 | 1 | 1 | 1 | 1 | 1 | 1 | 1 | 1 | 1 | 1 | 1 |
| Malvales | *Gossypium arboreum* | NC_016712 | 1 | 1 | 1 | 1 | 1 | 1 | 1 | 1 | 1 | 1 | 1 |
| Malvales | *Gossypium areysianum* | NC_018112 | 1 | 1 | 1 | 1 | 1 | 1 | 1 | 1 | 1 | 1 | 1 |
| Malvales | *Gossypium barbadense* | NC_008641 | 1 | 1 | 1 | 1 | 1 | 1 | 1 | 1 | 1 | 1 | 1 |
| Malvales | *Gossypium bickii* | NC_023214 | 1 | 1 | 1 | 1 | 1 | 1 | 1 | 1 | 1 | 1 | 1 |
| Malvales | *Gossypium capitis-viridis* | NC_018111 | 1 | 1 | 1 | 1 | 1 | 1 | 1 | 1 | 1 | 1 | 1 |
| Malvales | *Gossypium darwinii* | NC_016670 | 1 | 1 | 1 | 1 | 1 | 1 | 1 | 1 | 1 | 1 | 1 |
| Malvales | *Gossypium gossypioides* | NC_017894 | 1 | 1 | 1 | 1 | 1 | 1 | 1 | 1 | 1 | 1 | 1 |
| Malvales | *Gossypium herbaceum* | NC_023215 | 1 | 1 | 1 | 1 | 1 | 1 | 1 | 1 | 1 | 1 | 1 |
| Malvales | *Gossypium herbaceum* subsp*. africanum* | NC_016692 | 1 | 1 | 1 | 1 | 1 | 1 | 1 | 1 | 1 | 1 | 1 |
| Malvales | *Gossypium hirsutum* | NC_007944 | 1 | 1 | 1 | 1 | 1 | 1 | 1 | 1 | 1 | 1 | 1 |
| Malvales | *Gossypium incanum* | NC_018109 | 1 | 1 | 1 | 1 | 1 | 1 | 1 | 1 | 1 | 1 | 1 |
| Malvales | *Gossypium longicalyx* | NC_023216 | 1 | 1 | 1 | 1 | 1 | 1 | 1 | 1 | 1 | 1 | 1 |
| Malvales | *Gossypium mustelinum* | NC_016711 | 1 | 1 | 1 | 1 | 1 | 1 | 1 | 1 | 1 | 1 | 1 |
| Malvales | *Gossypium raimondii* | NC_016668 | 1 | 1 | 1 | 1 | 1 | 1 | 1 | 1 | 1 | 1 | 1 |
| Malvales | *Gossypium robinsonii* | NC_018113 | 1 | 1 | 1 | 1 | 1 | 1 | 1 | 1 | 1 | 1 | 1 |
| Malvales | *Gossypium somalense* | NC_018110 | 1 | 1 | 1 | 1 | 1 | 1 | 1 | 1 | 1 | 1 | 1 |
| Malvales | *Gossypium stocksii* | NC_023217 | 1 | 1 | 1 | 1 | 1 | 1 | 1 | 1 | 1 | 1 | 1 |
| Malvales | *Gossypium sturtianum* | NC_023218 | 1 | 1 | 1 | 1 | 1 | 1 | 1 | 1 | 1 | 1 | 1 |
| Malvales | *Gossypium thurberi* | NC_015204 | 1 | 1 | 1 | 1 | 1 | 1 | 1 | 1 | 1 | 1 | 1 |
| Malvales | *Gossypium tomentosum* | NC_016690 | 1 | 1 | 1 | 1 | 1 | 1 | 1 | 1 | 1 | 1 | 1 |
| Malvales | *Gossypium turneri* | NC_026835 | 1 | 1 | 1 | 1 | 1 | 1 | 1 | 1 | 1 | 1 | 1 |
| Malvales | *Hibiscus syriacus* | NC_026909 | 1 | 1 | 1 | 1 | 1 | 1 | 1 | 1 | 1 | 1 | 1 |
| Malvales | *Theobroma cacao* | NC_014676 | 1 | 1 | 1 | 1 | 1 | 1 | 1 | 1 | 1 | 1 | 1 |
| Malvales | *Tilia amurensis* | NC_028588 | 1 | 1 | 1 | 1 | 1 | 1 | 1 | 1 | 1 | 1 | 1 |
| Malvales | *Tilia mandshurica* | NC_028589 | 1 | 1 | 1 | 1 | 1 | 1 | 1 | 1 | 1 | 1 | 1 |
| Malvales | *Tilia oliveri* | NC_028590 | 1 | 1 | 1 | 1 | 1 | 1 | 1 | 1 | 1 | 1 | 1 |
| Malvales | *Tilia paucicostata* | NC_028591 | 1 | 1 | 1 | 1 | 1 | 1 | 1 | 1 | 1 | 1 | 1 |
| Brassicales | *Aethionema cordifolium* | NC_009265 | 1 | 1 | 1 | 1 | 1 | 1 | 1 | 1 | 1 | 1 | 1 |

Supplementary table S1. Continue

| Order | Species | Accession | *ndhA* | *ndhB* | *ndhC* | *ndhD* | *ndhE* | *ndhF* | *ndhG* | *ndhH* | *ndhI* | *ndhJ* | *ndhK* |
| --- | --- | --- | --- | --- | --- | --- | --- | --- | --- | --- | --- | --- | --- |
| Brassicales | *Aethionema grandiflorum* | NC_009266 | 1 | 1 | 1 | 1 | 1 | 1 | 1 | 1 | 1 | 1 | 1 |
| Brassicales | *Arabidopsis thaliana* | NC_000932 | 1 | 1 | 1 | 1 | 1 | 1 | 1 | 1 | 1 | 1 | 1 |
| Brassicales | *Arabis alpina* | NC_023367 | 1 | 1 | 1 | 1 | 1 | 1 | 1 | 1 | 1 | 1 | 1 |
| Brassicales | *Arabis hirsuta* | NC_009268 | 1 | 1 | 1 | 1 | 1 | 1 | 1 | 1 | 1 | 1 | 1 |
| Brassicales | *Barbarea verna* | NC_009269 | 1 | 1 | 1 | 1 | 1 | 1 | 1 | 1 | 1 | 1 | 1 |
| Brassicales | *Brassica juncea* | NC_028272 | 1 | 1 | 1 | 1 | 1 | 1 | 1 | 1 | 1 | 1 | 1 |
| Brassicales | *Brassica napus* | NC_016734 | 1 | 1 | 1 | 1 | 1 | 1 | 1 | 1 | 1 | 1 | 1 |
| Brassicales | *Capsella bursa-pastoris* | NC_009270 | 1 | 1 | 1 | 1 | 1 | 1 | 1 | 1 | 1 | 1 | 1 |
| Brassicales | *Capsella grandiflora* | NC_028517 | 1 | 1 | 1 | 1 | 1 | 1 | 1 | 1 | 1 | 1 | 1 |
| Brassicales | *Capsella rubella* | NC_027693 | 1 | 1 | 1 | 1 | 1 | 1 | 1 | 1 | 1 | 1 | 1 |
| Brassicales | *Cardamine impatiens* | NC_026445 | 1 | 1 | 1 | 1 | 1 | 1 | 1 | 1 | 1 | 1 | 1 |
| Brassicales | *Cardamine resedifolia* | NC_026446 | 1 | 1 | 1 | 1 | 1 | 1 | 1 | 1 | 1 | 1 | 1 |
| Brassicales | *Carica papaya* | NC_010323 | 1 | 1 | 1 | 1 | 1 | 1 | 1 | 1 | 1 | 1 | 1 |
| Brassicales | *Crucihimalaya wallichii* | NC_009271 | 1 | 1 | 1 | 1 | 1 | 1 | 1 | 1 | 1 | 1 | 1 |
| Brassicales | *Draba nemorosa* | NC_009272 | 1 | 1 | 1 | 1 | 1 | 1 | 1 | 1 | 1 | 1 | 1 |
| Brassicales | *Eutrema heterophyllum* | NC_028728 | 1 | 1 | 1 | 1 | 1 | 1 | 1 | 1 | 1 | 1 | 1 |
| Brassicales | *Eutrema salsugineum* | NC_028170 | 1 | 1 | 1 | 1 | 1 | 1 | 1 | 1 | 1 | 1 | 1 |
| Brassicales | *Eutrema yunnanense* | NC_028727 | 1 | 1 | 1 | 1 | 1 | 1 | 1 | 1 | 1 | 1 | 1 |
| Brassicales | *Isatis tinctoria* | NC_028415 | 1 | 1 | 1 | 1 | 1 | 1 | 1 | 1 | 1 | 1 | 1 |
| Brassicales | *Lepidium virginicum* | NC_009273 | 1 | 1 | 1 | 1 | 1 | 1 | 1 | 1 | 1 | 1 | 1 |
| Brassicales | *Lobularia maritima* | NC_009274 | 1 | 1 | 1 | 1 | 1 | 1 | 1 | 1 | 1 | 1 | 1 |
| Brassicales | *Nasturtium officinale* | NC_009275 | 1 | 1 | 1 | 1 | 1 | 1 | 1 | 1 | 1 | 1 | 1 |
| Brassicales | *Olimarabidopsis pumila* | NC_009267 | 1 | 1 | 1 | 1 | 1 | 1 | 1 | 1 | 1 | 1 | 1 |
| Brassicales | *Pachycladon cheesemanii* | NC_021102 | 1 | 1 | 1 | 1 | 1 | 1 | 1 | 1 | 1 | 1 | 1 |
| Brassicales | *Pachycladon enysii* | NC_018565 | 1 | 1 | 1 | 1 | 1 | 1 | 1 | 1 | 1 | 1 | 1 |
| Brassicales | *Raphanus sativus* | NC_024469 | 1 | 1 | 1 | 1 | 1 | 1 | 1 | 1 | 1 | 1 | 1 |
| Brassicales | *Schrenkiella parvula* | NC_028726 | 1 | 1 | 1 | 1 | 1 | 1 | 1 | 1 | 1 | 1 | 1 |
| Santalales | *Viscum minimum* | NC_027829 | 4 | 3 | 4 | 4 | 4 | 4 | 4 | 4 | 4 | 4 | 4 |
| Caryophyllales | *Agrostemma githago* | NC_023357 | 1 | 1 | 1 | 1 | 1 | 1 | 1 | 1 | 1 | 1 | 1 |
| Caryophyllales | *Carnegiea gigantea* | NC_027618 | 4 | 3 | 4 | 2 | 4 | 4 | 4 | 4 | 4 | 4 | 4 |
| Caryophyllales | *Colobanthus quitensis* | NC_028080 | 1 | 1 | 1 | 1 | 1 | 1 | 1 | 1 | 1 | 1 | 1 |
| Caryophyllales | *Fagopyrum esculentum* subsp*. ancestrale* | NC_010776 | 1 | 1 | 1 | 1 | 1 | 1 | 1 | 1 | 1 | 1 | 1 |
| Caryophyllales | *Fagopyrum tataricum* | NC_027161 | 1 | 1 | 1 | 1 | 1 | 1 | 1 | 1 | 1 | 1 | 1 |
| Caryophyllales | *Haloxylon ammodendron* | NC_027668 | 1 | 1 | 1 | 1 | 1 | 1 | 1 | 1 | 1 | 1 | 1 |
| Caryophyllales | *Haloxylon persicum* | NC_027669 | 1 | 1 | 1 | 1 | 1 | 1 | 1 | 1 | 1 | 1 | 1 |
| Caryophyllales | *Rheum palmatum* | NC_027728 | 1 | 1 | 1 | 1 | 1 | 1 | 1 | 1 | 1 | 1 | 1 |
| Caryophyllales | *Salicornia bigelovii* | NC_027226 | 1 | 1 | 1 | 1 | 1 | 1 | 1 | 1 | 1 | 1 | 1 |
| Caryophyllales | *Salicornia brachiata* | NC_027224 | 1 | 1 | 1 | 1 | 1 | 1 | 1 | 1 | 1 | 1 | 1 |
| Caryophyllales | *Salicornia europaea* | NC_027225 | 1 | 1 | 1 | 1 | 1 | 1 | 1 | 1 | 1 | 1 | 1 |
| Caryophyllales | *Silene chalcedonica* | NC_023359 | 1 | 1 | 1 | 1 | 1 | 1 | 1 | 1 | 1 | 1 | 1 |
| Caryophyllales | *Silene conica* | NC_016729 | 1 | 1 | 1 | 1 | 1 | 1 | 1 | 1 | 1 | 1 | 1 |
| Caryophyllales | *Silene conoidea* | NC_023358 | 1 | 1 | 1 | 1 | 1 | 1 | 1 | 1 | 1 | 1 | 1 |
| Caryophyllales | *Silene latifolia* | NC_016730 | 1 | 1 | 1 | 1 | 1 | 1 | 1 | 1 | 1 | 1 | 1 |
| Caryophyllales | *Silene noctiflora* | NC_016728 | 1 | 1 | 1 | 1 | 1 | 1 | 1 | 1 | 1 | 1 | 1 |
| Caryophyllales | *Silene paradoxa* | NC_023360 | 1 | 1 | 1 | 1 | 1 | 1 | 1 | 1 | 1 | 1 | 1 |
| Caryophyllales | *Silene vulgaris* | NC_016727 | 1 | 1 | 1 | 1 | 1 | 1 | 1 | 1 | 1 | 1 | 1 |
| Caryophyllales | *Spinacia oleracea* | NC_002202 | 1 | 1 | 1 | 1 | 1 | 1 | 1 | 1 | 1 | 1 | 1 |
| Ericales | *Actinidia chinensis* | NC_026690 | 1 | 1 | 1 | 1 | 1 | 1 | 1 | 1 | 1 | 1 | 1 |
| Ericales | *Actinidia deliciosa* | NC_026691 | 1 | 1 | 1 | 1 | 1 | 1 | 1 | 1 | 1 | 1 | 1 |
| Ericales | *Ardisia polysticta* | NC_021121 | 1 | 1 | 1 | 1 | 1 | 1 | 1 | 1 | 1 | 1 | 1 |
| Ericales | *Camellia crapnelliana* | NC_024541 | 1 | 1 | 1 | 1 | 1 | 1 | 1 | 1 | 1 | 1 | 1 |
| Ericales | *Camellia cuspidata* | NC_022459 | 1 | 1 | 1 | 1 | 1 | 1 | 1 | 1 | 1 | 1 | 1 |
| Ericales | *Camellia danzaiensis* | NC_022460 | 1 | 1 | 1 | 1 | 1 | 1 | 1 | 1 | 1 | 1 | 1 |
| Ericales | *Camellia grandibracteata* | NC_024659 | 1 | 1 | 1 | 1 | 1 | 1 | 1 | 1 | 1 | 1 | 1 |
| Ericales | *Camellia impressinervis* | NC_022461 | 1 | 1 | 1 | 1 | 1 | 1 | 1 | 1 | 1 | 1 | 1 |
| Ericales | *Camellia leptophylla* | NC_024660 | 1 | 1 | 1 | 1 | 1 | 1 | 1 | 1 | 1 | 1 | 1 |
| Ericales | *Camellia oleifera* | NC_023084 | 1 | 1 | 1 | 1 | 1 | 1 | 1 | 1 | 1 | 1 | 1 |
| Ericales | *Camellia petelotii* | NC_024661 | 1 | 1 | 1 | 1 | 1 | 1 | 1 | 1 | 1 | 1 | 1 |
| Ericales | *Camellia pitardii* | NC_022462 | 1 | 1 | 1 | 1 | 1 | 1 | 1 | 1 | 1 | 1 | 1 |
| Ericales | *Camellia pubicosta* | NC_024662 | 1 | 1 | 1 | 1 | 1 | 1 | 1 | 1 | 1 | 1 | 1 |
| Ericales | *Camellia reticulata* | NC_024663 | 1 | 1 | 1 | 1 | 1 | 1 | 1 | 1 | 1 | 1 | 1 |
| Ericales | *Camellia sinensis* | NC_020019 | 1 | 1 | 1 | 1 | 1 | 1 | 1 | 1 | 1 | 1 | 1 |
| Ericales | *Camellia taliensis* | NC_022264 | 1 | 1 | 1 | 1 | 1 | 1 | 1 | 1 | 1 | 1 | 1 |
| Ericales | *Camellia yunnanensis* | NC_022463 | 1 | 1 | 1 | 1 | 1 | 1 | 1 | 1 | 1 | 1 | 1 |
| Ericales | *Lysimachia coreana* | NC_026197 | 1 | 1 | 1 | 1 | 1 | 1 | 1 | 1 | 1 | 1 | 1 |
| Ericales | *Primula poissonii* | NC_024543 | 1 | 1 | 1 | 1 | 1 | 1 | 1 | 1 | 1 | 1 | 1 |
| Ericales | *Vaccinium macrocarpon* | NC_019616 | 1 | 1 | 1 | 1 | 1 | 1 | 2 | 1 | 1 | 1 | 2 |
| Gentianales | *Asclepias nivea* | NC_022431 | 1 | 1 | 1 | 1 | 1 | 1 | 1 | 1 | 1 | 1 | 1 |
| Gentianales | *Asclepias syriaca* | NC_022432 | 1 | 1 | 1 | 1 | 1 | 1 | 1 | 1 | 1 | 1 | 1 |
| Gentianales | *Catharanthus roseus* | NC_021423 | 1 | 1 | 1 | 1 | 1 | 1 | 1 | 1 | 1 | 1 | 1 |
| Gentianales | *Coffea arabica* | NC_008535 | 1 | 1 | 1 | 1 | 1 | 1 | 1 | 1 | 1 | 1 | 1 |
| Gentianales | *Echites umbellatus* | NC_025655 | 1 | 1 | 1 | 1 | 1 | 1 | 1 | 1 | 1 | 1 | 1 |
| Gentianales | *Gentiana crassicaulis* | NC_027442 | 1 | 1 | 1 | 1 | 1 | 1 | 1 | 1 | 1 | 1 | 1 |
| Gentianales | *Gentiana straminea* | NC_027441 | 1 | 1 | 1 | 1 | 1 | 1 | 1 | 1 | 1 | 1 | 1 |
| Gentianales | *Gynochthodes nanlingensis* | NC_028614 | 1 | 1 | 1 | 1 | 1 | 1 | 1 | 1 | 1 | 1 | 1 |
| Gentianales | *Gynochthodes officinalis* | NC_028009 | 1 | 1 | 1 | 1 | 1 | 1 | 1 | 1 | 1 | 1 | 1 |
| Gentianales | *Nerium oleander* | NC_025656 | 1 | 1 | 1 | 1 | 1 | 1 | 1 | 1 | 1 | 1 | 1 |
| Gentianales | *Oncinotis tenuiloba* | NC_025657 | 1 | 1 | 1 | 1 | 1 | 1 | 1 | 1 | 1 | 1 | 1 |
| Gentianales | *Pentalinon luteum* | NC_025658 | 1 | 1 | 1 | 1 | 1 | 1 | 1 | 1 | 1 | 1 | 1 |
| Gentianales | *Rhazya stricta* | NC_024292 | 1 | 1 | 1 | 1 | 1 | 1 | 1 | 1 | 1 | 1 | 1 |
| Solanales | *Atropa belladonna* | NC_004561 | 1 | 1 | 1 | 1 | 1 | 1 | 1 | 1 | 1 | 1 | 1 |
| Solanales | *Capsicum annuum* | NC_018552 | 1 | 1 | 1 | 1 | 1 | 1 | 1 | 1 | 1 | 1 | 1 |
| Solanales | *Capsicum frutescens* | NC_028007 | 1 | 1 | 1 | 1 | 1 | 1 | 1 | 1 | 1 | 1 | 1 |
| Solanales | *Capsicum lycianthoides* | NC_026551 | 1 | 1 | 1 | 1 | 1 | 1 | 1 | 1 | 1 | 1 | 1 |
| Solanales | *Cuscuta exaltata* | NC_009963 | 4 | 3 | 4 | 4 | 4 | 4 | 4 | 4 | 4 | 4 | 4 |
| Solanales | *Cuscuta gronovii* | NC_009765 | 4 | 4 | 4 | 4 | 4 | 4 | 4 | 4 | 4 | 4 | 4 |
| Solanales | *Cuscuta obtusiflora* | NC_009949 | 4 | 4 | 4 | 4 | 4 | 4 | 4 | 4 | 4 | 4 | 4 |
| Solanales | *Cuscuta reflexa* | NC_009766 | 4 | 3 | 4 | 4 | 4 | 4 | 4 | 4 | 4 | 3 | 4 |
| Solanales | *Datura stramonium* | NC_018117 | 1 | 1 | 1 | 1 | 1 | 1 | 1 | 1 | 1 | 1 | 1 |
| Solanales | *Dunalia brachyacantha* | NC_026906 | 1 | 1 | 1 | 1 | 1 | 1 | 1 | 1 | 1 | 1 | 1 |
| Solanales | *Dunalia obovata* | NC_026563 | 1 | 1 | 1 | 1 | 1 | 1 | 1 | 1 | 1 | 1 | 1 |
| Solanales | *Dunalia solanacea* | NC_027099 | 1 | 1 | 1 | 1 | 1 | 1 | 1 | 1 | 1 | 1 | 1 |
| Solanales | *Hyoscyamus niger* | NC_024261 | 1 | 1 | 1 | 1 | 1 | 1 | 1 | 1 | 1 | 1 | 1 |
| Solanales | *Iochroma loxense* | NC_026726 | 1 | 1 | 1 | 1 | 1 | 1 | 1 | 1 | 1 | 1 | 1 |
| Solanales | *Iochroma nitidum* | NC_026567 | 1 | 1 | 1 | 1 | 1 | 1 | 1 | 1 | 1 | 1 | 1 |
| Solanales | *Iochroma stenanthum* | NC_026574 | 1 | 1 | 1 | 1 | 1 | 1 | 1 | 1 | 1 | 1 | 1 |
| Solanales | *Iochroma tingoanum* | NC_027177 | 1 | 1 | 1 | 1 | 1 | 1 | 1 | 1 | 1 | 1 | 1 |
| Solanales | *Ipomoea batatas* | NC_026703 | 1 | 1 | 1 | 1 | 1 | 1 | 1 | 1 | 1 | 1 | 1 |
| Solanales | *Ipomoea purpurea* | NC_009808 | 1 | 1 | 1 | 1 | 1 | 1 | 1 | 1 | 1 | 1 | 1 |
| Solanales | *Nicotiana sylvestris* | NC_007500 | 1 | 1 | 1 | 1 | 1 | 1 | 1 | 1 | 1 | 1 | 1 |
| Solanales | *Nicotiana tabacum* | NC_001879 | 1 | 1 | 1 | 1 | 1 | 1 | 1 | 1 | 1 | 1 | 1 |
| Solanales | *Nicotiana tomentosiformis* | NC_007602 | 1 | 1 | 1 | 1 | 1 | 1 | 1 | 1 | 1 | 1 | 1 |
| Solanales | *Nicotiana undulata* | NC_016068 | 1 | 1 | 1 | 1 | 1 | 1 | 1 | 1 | 1 | 1 | 1 |
| Solanales | *Physalis peruviana* | NC_026570 | 1 | 1 | 1 | 1 | 1 | 1 | 1 | 1 | 1 | 1 | 1 |
| Solanales | *Saracha punctata* | NC_026694 | 1 | 1 | 1 | 1 | 1 | 1 | 1 | 1 | 1 | 1 | 1 |
| Solanales | *Solanum bulbocastanum* | NC_007943 | 1 | 1 | 1 | 1 | 1 | 1 | 1 | 1 | 1 | 1 | 1 |
| Solanales | *Solanum cheesmaniae* | NC_026876 | 1 | 1 | 1 | 1 | 1 | 1 | 1 | 1 | 1 | 1 | 1 |

Supplementary table S1. Continue

| Order | Species | Accession | *ndhA* | *ndhB* | *ndhC* | *ndhD* | *ndhE* | *ndhF* | *ndhG* | *ndhH* | *ndhI* | *ndhJ* | *ndhK* |
| --- | --- | --- | --- | --- | --- | --- | --- | --- | --- | --- | --- | --- | --- |
| Solanales | *Solanum chilense* | NC_026877 | 1 | 1 | 1 | 1 | 1 | 1 | 1 | 1 | 1 | 1 | 1 |
| Solanales | *Solanum commersonii* | NC_028069 | 1 | 1 | 1 | 1 | 1 | 1 | 1 | 1 | 1 | 1 | 1 |
| Solanales | *Solanum galapagense* | NC_026878 | 1 | 1 | 1 | 1 | 1 | 1 | 1 | 1 | 1 | 1 | 1 |
| Solanales | *Solanum habrochaites* | NC_026879 | 1 | 1 | 1 | 1 | 1 | 1 | 1 | 1 | 1 | 1 | 1 |
| Solanales | *Solanum lycopersicum* 1 | NC_007898 | 1 | 1 | 1 | 1 | 1 | 1 | 1 | 1 | 1 | 1 | 1 |
| Solanales | *Solanum lycopersicum* 2 | AC_000188 | 1 | 1 | 1 | 1 | 1 | 1 | 1 | 1 | 1 | 1 | 1 |
| Solanales | *Solanum neorickii* | NC_026880 | 1 | 1 | 1 | 1 | 1 | 1 | 1 | 1 | 1 | 1 | 1 |
| Solanales | *Solanum nigrum* | NC_028070 | 1 | 1 | 1 | 1 | 1 | 1 | 1 | 1 | 1 | 1 | 1 |
| Solanales | *Solanum peruvianum* | NC_026881 | 1 | 1 | 1 | 1 | 1 | 1 | 1 | 1 | 1 | 1 | 1 |
| Solanales | *Solanum pimpinellifolium* | NC_026882 | 1 | 1 | 1 | 1 | 1 | 1 | 1 | 1 | 1 | 1 | 1 |
| Solanales | *Solanum tuberosum* | NC_008096 | 1 | 1 | 1 | 1 | 1 | 1 | 1 | 1 | 1 | 1 | 1 |
| Lamiales | *Ajuga reptans* | NC_023102 | 1 | 1 | 1 | 1 | 1 | 1 | 1 | 1 | 1 | 1 | 1 |
| Lamiales | *Andrographis paniculata* | NC_022451 | 1 | 1 | 1 | 1 | 1 | 1 | 1 | 1 | 1 | 1 | 1 |
| Lamiales | *Boea hygrometrica* | NC_016468 | 1 | 1 | 1 | 1 | 1 | 1 | 1 | 1 | 1 | 1 | 1 |
| Lamiales | *Boulardia latisquama* | NC_025641 | 4 | 2 | 4 | 4 | 4 | 4 | 4 | 4 | 4 | 4 | 4 |
| Lamiales | *Cistanche deserticola* | NC_021111 | 4 | 3 | 4 | 4 | 4 | 3 | 4 | 3 | 4 | 4 | 4 |
| Lamiales | *Cistanche phelypaea* | NC_025642 | 4 | 2 | 4 | 4 | 4 | 4 | 4 | 4 | 4 | 4 | 4 |
| Lamiales | *Conopholis americana* | NC_023131 | 4 | 3 | 4 | 4 | 4 | 4 | 4 | 4 | 4 | 4 | 4 |
| Lamiales | *Epifagus virginiana* | NC_001568 | 4 | 3 | 4 | 4 | 4 | 4 | 4 | 4 | 4 | 4 | 4 |
| Lamiales | *Genlisea margaretae* | NC_025652 | 4 | 2 | 4 | 4 | 3 | 4 | 4 | 4 | 2 | 4 | 4 |
| Lamiales | *Hesperelaea palmeri* | NC_025787 | 1 | 1 | 1 | 1 | 1 | 1 | 1 | 1 | 1 | 1 | 1 |
| Lamiales | *Jasminum nudiflorum* | NC_008407 | 1 | 1 | 1 | 1 | 1 | 1 | 1 | 1 | 1 | 1 | 1 |
| Lamiales | *Lathraea squamaria* | NC_027838 | 2 | 2 | 1 | 3 | 1 | 2 | 2 | 2 | 2 | 2 | 2 |
| Lamiales | *Lindenbergia philippensis* | NC_022859 | 1 | 1 | 1 | 1 | 1 | 1 | 1 | 1 | 1 | 1 | 1 |
| Lamiales | *Olea europaea* | NC_013707 | 1 | 1 | 1 | 1 | 1 | 1 | 1 | 1 | 1 | 1 | 1 |
| Lamiales | *Olea europaea* subsp*. cuspidata* | NC_015604 | 1 | 1 | 1 | 1 | 1 | 1 | 1 | 1 | 1 | 1 | 1 |
| Lamiales | *Olea europaea* subsp*. europaea* | NC_015401 | 1 | 1 | 1 | 1 | 1 | 1 | 1 | 1 | 1 | 1 | 1 |
| Lamiales | *Olea europaea* subsp*. maroccana* | NC_015623 | 1 | 1 | 1 | 1 | 1 | 1 | 1 | 1 | 1 | 1 | 1 |
| Lamiales | *Olea woodiana* subsp*. woodiana* | NC_015608 | 1 | 1 | 1 | 1 | 1 | 1 | 1 | 1 | 1 | 1 | 1 |
| Lamiales | *Orobanche californica* | NC_025651 | 4 | 1 | 4 | 4 | 2 | 4 | 3 | 4 | 4 | 2 | 3 |
| Lamiales | *Orobanche crenata* | NC_024845 | 4 | 3 | 4 | 4 | 4 | 4 | 4 | 4 | 4 | 4 | 4 |
| Lamiales | *Orobanche gracilis* | NC_023464 | 4 | 3 | 4 | 4 | 4 | 4 | 4 | 4 | 4 | 4 | 4 |
| Lamiales | *Orobanche purpurea* | NC_023132 | 4 | 3 | 4 | 4 | 4 | 4 | 4 | 4 | 4 | 4 | 4 |
| Lamiales | *Orobanche ramosa* | NC_023465 | 4 | 4 | 4 | 4 | 4 | 4 | 4 | 4 | 4 | 4 | 4 |
| Lamiales | *Pinguicula ehlersiae* | NC_023463 | 3 | 1 | 4 | 3 | 2 | 4 | 2 | 2 | 2 | 2 | 3 |
| Lamiales | *Plantago maritima* | NC_028519 | 1 | 1 | 1 | 1 | 1 | 1 | 1 | 1 | 1 | 1 | 1 |
| Lamiales | *Plantago media* | NC_028520 | 1 | 1 | 1 | 1 | 1 | 1 | 1 | 1 | 1 | 1 | 1 |
| Lamiales | *Premna microphylla* | NC_026291 | 1 | 1 | 1 | 1 | 1 | 1 | 1 | 1 | 1 | 1 | 1 |
| Lamiales | *Rosmarinus officinalis* | NC_027259 | 1 | 1 | 1 | 1 | 1 | 1 | 1 | 1 | 1 | 1 | 1 |
| Lamiales | *Salvia miltiorrhiza* | NC_020431 | 1 | 1 | 1 | 1 | 1 | 1 | 1 | 1 | 1 | 1 | 1 |
| Lamiales | *Schwalbea americana* | NC_023115 | 3 | 1 | 1 | 2 | 1 | 3 | 2 | 1 | 3 | 2 | 1 |
| Lamiales | *Scrophularia takesimensis* | NC_026202 | 1 | 1 | 1 | 1 | 1 | 1 | 1 | 1 | 1 | 1 | 1 |
| Lamiales | *Scutellaria baicalensis* | NC_027262 | 1 | 1 | 1 | 1 | 1 | 1 | 1 | 1 | 1 | 1 | 1 |
| Lamiales | *Scutellaria insignis* | NC_028533 | 1 | 1 | 1 | 1 | 1 | 1 | 1 | 1 | 1 | 1 | 1 |
| Lamiales | *Sesamum indicum* | NC_016433 | 1 | 1 | 1 | 1 | 1 | 1 | 1 | 1 | 1 | 1 | 1 |
| Lamiales | *Tanaecium tetragonolobum* | NC_027955 | 1 | 1 | 1 | 1 | 1 | 1 | 1 | 1 | 1 | 1 | 1 |
| Lamiales | *Tectona grandis* | NC_020098 | 1 | 1 | 1 | 1 | 1 | 1 | 1 | 1 | 1 | 1 | 1 |
| Lamiales | *Utricularia gibba* | NC_021449 | 1 | 1 | 1 | 1 | 1 | 1 | 1 | 1 | 1 | 1 | 1 |
| Lamiales | *Utricularia macrorhiza* | NC_025653 | 1 | 1 | 1 | 1 | 1 | 1 | 1 | 1 | 1 | 1 | 1 |
| Asterales | *Adenophora remotiflora* | NC_026999 | 1 | 2 | 1 | 1 | 1 | 1 | 1 | 1 | 1 | 1 | 1 |
| Asterales | *Ageratina adenophora* | NC_015621 | 1 | 1 | 1 | 1 | 1 | 1 | 1 | 1 | 1 | 1 | 1 |
| Asterales | *Artemisia frigida* | NC_020607 | 1 | 1 | 1 | 1 | 1 | 1 | 1 | 1 | 1 | 1 | 1 |
| Asterales | *Artemisia montana* | NC_025910 | 1 | 1 | 1 | 1 | 1 | 1 | 1 | 1 | 1 | 1 | 1 |
| Asterales | *Aster spathulifolius* | NC_027434 | 1 | 1 | 1 | 1 | 1 | 1 | 1 | 1 | 1 | 1 | 1 |
| Asterales | *Brighamia insignis* | NC_028633 | 1 | 1 | 1 | 1 | 1 | 1 | 1 | 1 | 1 | 1 | 1 |
| Asterales | *Campanula takesimana* | NC_026203 | 1 | 1 | 1 | 1 | 1 | 1 | 1 | 1 | 1 | 1 | 1 |
| Asterales | *Centaurea diffusa* | NC_024286 | 1 | 1 | 1 | 1 | 1 | 1 | 1 | 1 | 1 | 1 | 1 |
| Asterales | *Chrysanthemum indicum* | NC_020320 | 1 | 1 | 1 | 1 | 1 | 1 | 1 | 1 | 1 | 1 | 1 |
| Asterales | *Chrysanthemum x morifolium* | NC_020092 | 1 | 1 | 1 | 1 | 1 | 1 | 1 | 1 | 1 | 1 | 1 |
| Asterales | *Cynara baetica* | NC_028005 | 1 | 1 | 1 | 1 | 1 | 1 | 1 | 1 | 1 | 1 | 1 |
| Asterales | *Cynara cornigera* | NC_028006 | 1 | 1 | 1 | 1 | 1 | 1 | 1 | 1 | 1 | 1 | 1 |
| Asterales | *Cynara humilis* | NC_027113 | 1 | 1 | 1 | 1 | 1 | 1 | 1 | 1 | 1 | 1 | 1 |
| Asterales | *Guizotia abyssinica* | NC_010601 | 1 | 1 | 1 | 1 | 1 | 1 | 1 | 1 | 1 | 1 | 1 |
| Asterales | *Hanabusaya asiatica* | NC_024732 | 1 | 2 | 1 | 1 | 1 | 1 | 1 | 1 | 1 | 1 | 1 |
| Asterales | *Helianthus annuus* | NC_007977 | 1 | 1 | 1 | 1 | 1 | 1 | 1 | 1 | 1 | 1 | 1 |
| Asterales | *Helianthus decapetalus* | NC_023110 | 1 | 1 | 1 | 1 | 1 | 1 | 1 | 1 | 1 | 1 | 1 |
| Asterales | *Helianthus divaricatus* | NC_023109 | 1 | 1 | 1 | 1 | 1 | 1 | 1 | 1 | 1 | 1 | 1 |
| Asterales | *Helianthus giganteus* | NC_023107 | 1 | 1 | 1 | 1 | 1 | 1 | 1 | 1 | 1 | 1 | 1 |
| Asterales | *Helianthus grosseserratus* | NC_023108 | 1 | 1 | 1 | 1 | 1 | 1 | 1 | 1 | 1 | 1 | 1 |
| Asterales | *Helianthus hirsutus* | NC_023111 | 1 | 1 | 1 | 1 | 1 | 1 | 1 | 1 | 1 | 1 | 1 |
| Asterales | *Helianthus maximiliani* | NC_023114 | 1 | 1 | 1 | 1 | 1 | 1 | 1 | 1 | 1 | 1 | 1 |
| Asterales | *Helianthus strumosus* | NC_023113 | 1 | 1 | 1 | 1 | 1 | 1 | 1 | 1 | 1 | 1 | 1 |
| Asterales | *Helianthus tuberosus* | NC_023112 | 1 | 1 | 1 | 1 | 1 | 1 | 1 | 1 | 1 | 1 | 1 |
| Asterales | *Jacobaea vulgaris* | NC_015543 | 1 | 1 | 1 | 1 | 1 | 1 | 1 | 1 | 1 | 1 | 1 |
| Asterales | *Lactuca sativa* | NC_007578 | 1 | 1 | 1 | 1 | 1 | 1 | 1 | 1 | 1 | 1 | 1 |
| Asterales | *Leontopodium leiolepis* | NC_027835 | 1 | 1 | 1 | 1 | 1 | 1 | 1 | 1 | 1 | 1 | 1 |
| Asterales | *Parthenium argentatum* | NC_013553 | 2 | 1 | 1 | 1 | 1 | 2 | 1 | 1 | 1 | 1 | 1 |
| Asterales | *Praxelis clematidea* | NC_023833 | 1 | 1 | 1 | 1 | 1 | 1 | 1 | 1 | 1 | 1 | 1 |
| Asterales | *Silybum marianum* | NC_028027 | 1 | 1 | 1 | 1 | 1 | 1 | 1 | 1 | 1 | 1 | 1 |
| Asterales | *Trachelium caeruleum* | NC_010442 | 1 | 1 | 1 | 1 | 1 | 1 | 1 | 1 | 1 | 1 | 1 |
| Apiales | *Anthriscus cerefolium* | NC_015113 | 1 | 1 | 1 | 1 | 1 | 1 | 1 | 1 | 1 | 1 | 1 |
| Apiales | *Aralia undulata* | NC_022810 | 1 | 1 | 1 | 1 | 1 | 1 | 1 | 1 | 1 | 1 | 1 |
| Apiales | *Brassaiopsis hainla* | NC_022811 | 1 | 1 | 1 | 1 | 1 | 1 | 1 | 1 | 1 | 1 | 1 |
| Apiales | *Bupleurum falcatum* | NC_027834 | 1 | 1 | 1 | 1 | 1 | 1 | 1 | 1 | 1 | 1 | 1 |
| Apiales | *Daucus carota* | NC_008325 | 1 | 1 | 1 | 1 | 1 | 1 | 1 | 1 | 1 | 1 | 1 |
| Apiales | *Dendropanax dentiger* | NC_026546 | 1 | 1 | 1 | 1 | 1 | 1 | 1 | 1 | 1 | 1 | 1 |
| Apiales | *Dendropanax morbifer* | NC_027607 | 1 | 1 | 1 | 1 | 1 | 1 | 1 | 1 | 1 | 1 | 1 |
| Apiales | *Eleutherococcus senticosus* | NC_016430 | 1 | 1 | 1 | 1 | 1 | 1 | 1 | 1 | 1 | 1 | 1 |
| Apiales | *Fatsia japonica* | NC_027685 | 1 | 1 | 1 | 1 | 1 | 1 | 1 | 1 | 1 | 1 | 1 |
| Apiales | *Kalopanax septemlobus* | NC_022814 | 1 | 1 | 1 | 1 | 1 | 1 | 1 | 1 | 1 | 1 | 1 |
| Apiales | *Metapanax delavayi* | NC_022812 | 1 | 1 | 1 | 1 | 1 | 1 | 1 | 1 | 1 | 1 | 1 |
| Apiales | *Ostericum grosseserratum* | NC_028618 | 1 | 1 | 1 | 1 | 1 | 1 | 1 | 1 | 1 | 1 | 1 |
| Apiales | *Panax ginseng* | NC_006290 | 1 | 1 | 1 | 1 | 1 | 1 | 1 | 1 | 1 | 1 | 1 |
| Apiales | *Panax japonicus* | NC_028703 | 1 | 1 | 1 | 1 | 1 | 1 | 1 | 1 | 1 | 1 | 1 |
| Apiales | *Panax notoginseng* | NC_026447 | 1 | 1 | 1 | 1 | 1 | 1 | 1 | 1 | 1 | 1 | 1 |
| Apiales | *Panax quinquefolius* | NC_027456 | 1 | 1 | 1 | 1 | 1 | 1 | 1 | 1 | 1 | 1 | 1 |
| Apiales | *Panax vietnamensis* | NC_028704 | 1 | 1 | 1 | 1 | 1 | 1 | 1 | 1 | 1 | 1 | 1 |
| Apiales | *Pastinaca pimpinellifolia* | NC_027450 | 1 | 1 | 1 | 1 | 1 | 1 | 1 | 1 | 1 | 1 | 1 |
| Apiales | *Schefflera delavayi* | NC_022813 | 1 | 1 | 1 | 1 | 1 | 1 | 1 | 1 | 1 | 1 | 1 |
| Apiales | *Seseli montanum* | NC_027451 | 1 | 1 | 1 | 1 | 1 | 1 | 1 | 1 | 1 | 1 | 1 |
| Dipsacales | *Lonicera japonica* | NC_026839 | 1 | 1 | 1 | 1 | 1 | 1 | 1 | 1 | 1 | 1 | 1 |
