# Supplementary material for: Independent degradation in genes of the plastid ndh gene family in species of the orchid genus Cymbidium (Orchidaceae; Epidendroideae)
Source: PLoS One. 2017 Nov 15;12(11):e0187318. doi: 10.1371/journal.pone.0187318 (PMC5695243; doi:10.1371/journal.pone.0187318)
Supplement: S2 Table — (DOCX) [file pone.0187318.s006.docx]

Table S2. The 55 plastome sequences for phylogenetic study of *ndh* genes.

| Subfamily | Species | Accession |
| --- | --- | --- |
| Vanilloideae | *Vanilla planifolia* | NC_026778 |
| Cypripedioideae | *Cypripedium japonicum* | NC_027227 |
|  | *Paphiopedilum armeniacum* | KT388109 |
|  | *Paphiopedilum armeniacum* | NC_026779 |
|  | *Paphiopedilum niveum* | NC_026776 |
|  | *Phragmipedium longifolium* | NC_028149 |
| Orchidoideae | *Goodyera fumata* | NC_026773 |
|  | *Goodyera procera* | NC_029363 |
|  | *Goodyera schlechtendaliana* | NC_029364 |
|  | *Goodyera velutina* | NC_029365 |
|  | *Habenaria pantlingiana* | NC_026775 |
| Epidendroideae | *Bletilla ochracea* | NC_029483 |
|  | *Bletilla striata* | NC_028422 |
|  | *Calanthe triplicata* | NC_024544 |
|  | *Cymbidium aloifolium* | NC_021429 |
|  | *Cymbidium ensifolium* 1 | KU179434 |
|  | *Cymbidium ensifolium* 2 | NC_028525 |
|  | *Cymbidium faberi* | NC_027743 |
|  | *Cymbidium goeringii* | NC_028524 |
|  | *Cymbidium kanran* | NC_029711 |
|  | *Cymbidium lancifolium* | NC_029712 |
|  | *Cymbidium macrorhizon* | NC_029713 |
|  | *Cymbidium mannii* | NC_021433 |
|  | *Cymbidium sinense* | NC_021430 |
|  | *Cymbidium tortisepalum* | NC_021431 |
|  | *Cymbidium tracyanum* | NC_021432 |
|  | *Erycina pusilla* | NC_018114 |
|  | *Oncidium hybrid cultivar* | NC_014056 |
|  | *Oncidium sphacelatum* | NC_028148 |
|  | *Corallorhiza bulbosa* | NC_025659 |
|  | *Corallorhiza macrantha* | NC_025660 |
|  | *Corallorhiza mertensiana* | NC_025661 |
|  | *Corallorhiza odontorhiza* | NC_025664 |
|  | *Corallorhiza striata* var*. vreelandii* | JX087681 |
|  | *Corallorhiza trifida* | NC_025662 |
|  | *Corallorhiza wisteriana* | NC_025663 |
|  | *Cattleya crispata* | NC_026568 |
|  | *Masdevallia coccinea* | NC_026541 |
|  | *Masdevallia picturata* | NC_026777 |
|  | *Dendrobium catenatum* | NC_024019 |
|  | *Dendrobium chrysotoxum* | NC_028549 |
|  | *Dendrobium huoshanense* | NC_028430 |
|  | *Dendrobium nobile* | NC_029456 |
|  | *Dendrobium pendulum* | NC_029705 |
|  | *Dendrobium strongylanthum* | NC_027691 |
|  | *Elleanthus sodiroi* | NC_027266 |
|  | *Sobralia* aff*. bouchei* | NC_028209 |
|  | *Sobralia callosa* | NC_028147 |
|  | *Phalaenopsis aphrodite* subsp*. formosana* | NC_007499 |
|  | *Phalaenopsis equestris* | NC_017609 |
|  | *Phalaenopsis* hybrid cultivar | NC_025593 |
| Outgroup | *Allium cepa* | NC_024813 |
|  | *Cypripedium macranthos* (*Hosta* sp.) | NC_024421 |
|  | *Eustrephus latifolius* | NC_025305 |
|  | *Iris gatesii* | NC_024936 |
