# Supplementary material for: Independent degradation in genes of the plastid ndh gene family in species of the orchid genus Cymbidium (Orchidaceae; Epidendroideae)
Source: PLoS One. 2017 Nov 15;12(11):e0187318. doi: 10.1371/journal.pone.0187318 (PMC5695243; doi:10.1371/journal.pone.0187318)
Supplement: S3 Table — (DOCX) [file pone.0187318.s007.docx]

Table S3. The mt-*ndh* genes for phylogenetic study of *ndh* genes.

| Taxon | Gene | Accession | Reference | Taxon | Gene | Accession | Reference | Taxon | Gene | Accession | Reference |
| --- | --- | --- | --- | --- | --- | --- | --- | --- | --- | --- | --- |
| *Cypripedium formosanum* | *ndhC* | KJ501922 | Lin et al. (2015) | *Masdevallia picturata* | *ndhK* | KJ501929 | Lin et al. (2015) | *Cymbidium lancifolium* | *ndhI* | KX962298 | this paper |
| *Cypripedium formosanum* | *ndhF* | KJ501956 | Lin et al. (2015) | *Masdevallia picturata* | *ndhJ* | KJ501929 | Lin et al. (2015) | *Cymbidium lancifolium* | *ndhA* | KX962298 | this paper |
| *Erycina pusilla* | *ndhC* | KJ501950 | Lin et al. (2015) | *Masdevallia picturata* | *ndhC* | KJ501930 | Lin et al. (2015) | *Cymbidium lancifolium* | *ndhH* | KX962298 | this paper |
| *Erycina pusilla* | *ndK* | KJ501950 | Lin et al. (2015) | *Masdevallia picturata* | *ndhD* | KJ501946 | Lin et al. (2015) | *Cymbidium lancifolium* | *ndhC* | KX962301 | this paper |
| *Erycina pusilla* | *ndhJ* | KJ501950 | Lin et al. (2015) | *Masdevallia picturata* | *ndhE* | KJ501946 | Lin et al. (2015) | *Cymbidium lancifolium* | *ndhK* | KX962301 | this paper |
| *Erycina pusilla* | *ndhD* | KJ501958 | Lin et al. (2015) | *Masdevallia picturata* | *ndhG* | KJ501946 | Lin et al. (2015) | *Cymbidium lancifolium* | *ndhJ* | KX962301 | this paper |
| *Erycina pusilla* | *ndhE* | KJ501958 | Lin et al. (2015) | *Masdevallia picturata* | *ndhI* | KJ501946 | Lin et al. (2015) | *Cymbidium lancifolium* | *ndhD* | KX962297 | this paper |
| *Erycina pusilla* | *ndhG* | KJ501958 | Lin et al. (2015) | *Masdevallia picturata* | *ndhA* | KJ501946 | Lin et al. (2015) | *Cymbidium lancifolium* | *ndhD* | KX962298 | this paper |
| *Erycina pusilla* | *ndhI* | KJ501958 | Lin et al. (2015) | *Masdevallia picturata* | *ndhH* | KJ501946 | Lin et al. (2015) | *Cymbidium lancifolium* | *ndhF* | KX962299 | this paper |
| *Erycina pusilla* | *ndhA* | KJ501958 | Lin et al. (2015) | *Masdevallia picturata* | *ndhB* | KJ501947 | Lin et al. (2015) | *Cymbidium lancifolium* | *ndhF* | KX962300 | this paper |
| *Erycina pusilla* | *ndhH* | KJ501958 | Lin et al. (2015) | *Oncidium Gower Ramsey* | *ndhF* | KJ501953 | Lin et al. (2015) | *Dendrobium catenatum* | *ndhF* | KX962308 | this paper |
| *Erycina pusilla* | *ndhF* | KJ501966 | Lin et al. (2015) | *Oncidium Gower Ramsey* | *ndhD* | KJ501925 | Lin et al. (2015) | *Dendrobium catenatum* | *ndhA* | KX962306 | this paper |
| *Erycina pusilla* | *ndhD* | KJ501966 | Lin et al. (2015) | *Oncidium Gower Ramsey* | *ndhE* | KJ501925 | Lin et al. (2015) | *Dendrobium catenatum* | *ndhI* | KX962306 | this paper |
| *Erycina pusilla* | *ndhC* | KJ501968 | Lin et al. (2015) | *Oncidium Gower Ramsey* | *ndhB* | KJ501920 | Lin et al. (2015) | *Dendrobium catenatum* | *ndhA* | KX962306 | this paper |
| *Erycina pusilla* | *ndhK* | KJ501968 | Lin et al. (2015) | *Paphiopedium armeniacum* | *ndhD* | KJ501961 | Lin et al. (2015) | *Dendrobium catenatum* | *ndhH* | KX962306 | this paper |
| *Erycina pusilla* | *ndhJ* | KJ501968 | Lin et al. (2015) | *Paphiopedium armeniacum* | *ndhC* | KJ501961 | Lin et al. (2015) | *Dendrobium catenatum* | *ndhD* | KX962307 | this paper |
| *Erycina pusilla* | *ndhD* | KJ501969 | Lin et al. (2015) | *Paphiopedium armeniacum* | *ndhA* | KJ501962 | Lin et al. (2015) | *Dendrobium catenatum* | *ndhE* | KX962307 | this paper |
| *Erycina pusilla* | *ndhE* | KJ501969 | Lin et al. (2015) | *Paphiopedium armeniacum* | *ndhH* | KJ501963 | Lin et al. (2015) | *Dendrobium catenatum* | *ndhG* | KX962307 | this paper |
| *Erycina pusilla* | *ndhG* | KJ501969 | Lin et al. (2015) | *Paphiopedium armeniacum* | *ndhB* | KJ501951 | Lin et al. (2015) | *Dendrobium catenatum* | *ndhJ* | KX962309 | this paper |
| *Erycina pusilla* | *ndhI* | KJ501969 | Lin et al. (2015) | *Paphiopedium niveum* | *ndhD* | KJ501945 | Lin et al. (2015) | *Dendrobium catenatum* | *ndhK* | KX962309 | this paper |
| *Erycina pusilla* | *ndhA* | KJ501969 | Lin et al. (2015) | *Paphiopedium niveum* | *ndhC* | KJ501944 | Lin et al. (2015) | *Dendrobium catenatum* | *ndhC* | KX962309 | this paper |
| *Erycina pusilla* | *ndhH* | KJ501969 | Lin et al. (2015) | *Paphiopedium niveum* | *ndhK* | KJ501944 | Lin et al. (2015) | *Epipogium aphyllum* | *ndhI* | KX962310 | this paper |
| *Erycina pusilla* | *ndhD* | KJ501975 | Lin et al. (2015) | *Paphiopedium niveum* | *ndhJ* | KJ501944 | Lin et al. (2015) | *Epipogium aphyllum* | *ndhA* | KX962310 | this paper |
| *Erycina pusilla* | *ndhD* | KJ501978 | Lin et al. (2015) | *Vanilla planifolia* | *ndhJ* | KJ501948 | Lin et al. (2015) | *Epipogium aphyllum* | *ndhA* | KX962310 | this paper |
| *Erycina pusilla* | *ndhE* | KJ501978 | Lin et al. (2015) | *Vanilla planifolia* | *ndhK* | KJ501957 | Lin et al. (2015) | *Cymbidium devonianum* | *ndhD* | KX962295 | this paper |
| *Erycina pusilla* | *ndhG* | KJ501978 | Lin et al. (2015) | *Vanilla planifolia* | *ndhC* | KJ501923 | Lin et al. (2015) | *Cymbidium devonianum* | *ndhE* | KX962294 | this paper |
| *Erycina pusilla* | *ndhI* | KJ501978 | Lin et al. (2015) | *Vanilla planifolia* | *ndhE* | KJ501959 | Lin et al. (2015) | *Cymbidium devonianum* | *ndhG* | KX962294 | this paper |
| *Erycina pusilla* | *ndhA* | KJ501978 | Lin et al. (2015) | *Goodyera fumata* | *ndhJ* | KJ501949 | Lin et al. (2015) | *Cymbidium ensifolium* | *ndhD* | KX962291 | this paper |
| *Erycina pusilla* | *ndhC* | KJ501991 | Lin et al. (2015) | *Goodyera fumata* | *ndhC* | KJ501924 | Lin et al. (2015) | *Cymbidium ensifolium* | *ndhE* | KX962290 | this paper |
| *Erycina pusilla* | *ndhK* | KJ501991 | Lin et al. (2015) | *Cymbidium macrorhizon* | *ndhB* | KX962302 | this paper | *Cymbidium ensifolium* | *ndhG* | KX962290 | this paper |
| *Erycina pusilla* | *ndhJ* | KJ501991 | Lin et al. (2015) | *Cymbidium macrorhizon* | *ndhC* | KX962305 | this paper | *Cymbidium finlaysonianum* | *ndhD* | KX962293 | this paper |
| *Erycina pusilla* | *ndhB* | KJ501994 | Lin et al. (2015) | *Cymbidium macrorhizon* | *ndhK* | KX962305 | this paper | *Cymbidium finlaysonianum* | *ndhE* | KX962292 | this paper |
| *Erycina pusilla* | *ndhC* | KJ501996 | Lin et al. (2015) | *Cymbidium macrorhizon* | *ndhJ* | KX962305 | this paper | *Cymbidium finlaysonianum* | *ndhG* | KX962292 | this paper |
| *Goodyera fumata* | *ndhH* | KJ501955 | Lin et al. (2015) | *Cymbidium macrorhizon* | *ndhD* | KX962303 | this paper | *Cymbidium kanran* | *ndhD* | KX962288 | this paper |
| *Goodyera fumata* | *ndhA* | KJ501963 | Lin et al. (2015) | *Cymbidium macrorhizon* | *ndhE* | KX962303 | this paper | *Cymbidium kanran* | *ndhE* | KX962289 | this paper |
| *Goodyera fumata* | *ndhI* | KJ501963 | Lin et al. (2015) | *Cymbidium macrorhizon* | *ndhG* | KX962303 | this paper | *Cymbidium kanran* | *ndhG* | KX962289 | this paper |
| *Goodyera fumata* | *ndhE* | KJ501963 | Lin et al. (2015) | *Cymbidium macrorhizon* | *ndhI* | KX962303 | this paper | *Cymbidium lancifolium* | *ndhD* | KX962285 | this paper |
| *Goodyera fumata* | *ndhD* | KJ501927 | Lin et al. (2015) | *Cymbidium macrorhizon* | *ndhA* | KX962303 | this paper | *Cymbidium lancifolium* | *ndhE* | KX962284 | this paper |
| *Goodyera fumata* | *ndhB* | KJ501921 | Lin et al. (2015) | *Cymbidium macrorhizon* | *ndhH* | KX962303 | this paper | *Cymbidium lancifolium* | *ndhG* | KX962284 | this paper |
| *Habenaria longidenticulata* | *ndhF* | KJ501954 | Lin et al. (2015) | *Cymbidium macrorhizon* | *ndhF* | KX962304 | this paper | *Cymbidium sinense* | *ndhD* | KX962286 | this paper |
| *Habenaria longidenticulata* | *ndhD* | KJ501926 | Lin et al. (2015) | *Cymbidium macrorhizon* | *ndhF* | KX962304 | this paper | *Cymbidium sinense* | *ndhE* | KX962287 | this paper |
| *Habenaria longidenticulata* | *ndhE* | KJ501926 | Lin et al. (2015) | *Cymbidium lancifolium* | *ndhB* | KX962296 | this paper | *Cymbidium sinense* | *ndhG* | KX962287 | this paper |
| *Habenaria longidenticulata* | *ndhA* | KJ501960 | Lin et al. (2015) | *Cymbidium lancifolium* | *ndhE* | KX962298 | this paper |  |  |  |  |
| *Masdevallia picturata* | *ndhF* | KJ501928 | Lin et al. (2015) | *Cymbidium lancifolium* | *ndhG* | KX962298 | this paper |  |  |  |  |
